# Significance of the Stability of Fusarium Head Blight Resistance in the Variety Registration, Breeding, and Genetic Research of Winter Wheat Using Disease Index, Fusarium-Damaged Kernels, and Deoxynivalenol Contamination

**DOI:** 10.3390/toxins17060288

**Published:** 2025-06-06

**Authors:** Ákos Mesterhazy, Beata Tóth, Attila Berényi, Katalin Ács, Tamas Meszlényi

**Affiliations:** Cereal Research Non-Profit Ltd., P.O. Box 391, H-6701 Szeged, Hungary; beata.cseuz.toth@gmail.com (B.T.); katalin.acs@gabonakutato.hu (K.Á.); tamas.meszlenyi@gabonakutato.hu (T.M.)

**Keywords:** Fusarium head blight, stability of resistance, *F. graminearum*, *F. culmorum*, disease index, Fusarium-damaged kernels, deoxynivalenol, adaptation to FHB, variety registration, reliable testing of resistance

## Abstract

Fusarium head blight is one of the greatest threats to global wheat production. Despite the special attention paid by researchers to resistance genetics, the stability of resistance and the expression of its epidemiological relationships have not been tested in depth. As most studies only present data on visual symptoms, in this study, we present data from four experiments. Here, 15–40 genotypes were tested with four and eight isolates (inocula) in 3–4-year experiments, with 32, 24, 36, and 12 epidemic situations used to determine the disease index (DI), Fusarium-damaged kernels (FDKs), and DON. All genotypes were tested for stability by the variance across epidemics, and the b value of the linear function was considered. Both indices were suitable for measuring stability/instability, but the variance results were more closely correlated with the experimental data than the b value, known as the stability index (SI). The use of variance is recommended due to its simplicity and reliability. In the first test, the rate of maximum/minimum variance for DI, FDK, and DON differed 15-, 20-, and 120-fold, respectively. In the second test, the same rates were 200, 400, and over 4000, with the other tests exhibiting similar tendencies. The traits differ, the epidemics vary, and a dependence on resistance level can be proven. The genotype ranking varies strongly in different epidemics, with approximately 50% of the correlations between variety responses being insignificant. Therefore, many epidemics are needed to obtain a reliable picture of the adaptation ability of the resistance traits and their stability. Approximately 25% of the genotypes tested belong to the most stable group. About 35% were discarded, and in the 40% medium, we observed both highly unstable and moderately stable genotypes. Principal component analysis (PCA) of the three traits in the experiments showed a confirmatory, nearly uniform distribution of genotypes, with a different footprint or “identity card” present for each genotype. The genotypes for the traits belong to one or two groups, although sometimes individual genotypes seem to be independent. No strict rule was found. This underlines the necessity of considering the plant’s traits (Di, FDK, and DON) in resistance testing. Highly resistant winter wheat lines could also be bred with very low variance and SI values and very high stability (SI values lower than 0.3). Of the traits, DON is the most important. With this methodology, variety registration also becomes possible. The epidemiological aspect has a decisive role in resistance studies, and without identifying stability in FHB resistance, no food safety estimates can be made.

## 1. Introduction

Fusarium head blight (FHB) is one of the most significant wheat diseases and is responsible for causing some of the most severe losses in wheat production [1]. According to existing data released by the FAO [2,3], approximately 25% of harvested grain, including wheat, is contaminated with toxins. The global grain yield in 2018 was 2250 million tons; of this, 800 million tons was wheat, and similar yields have been seen since then, with increasing tendency. Of this yield, 200 million tons is estimated to be lost due to toxins. According to Mesterhazy et al. [4], 80 million metric tons (10%) of wheat suffers contamination before harvest and storage. An additional 120 million tons deteriorates in storage facilities. Following uncontrolled mixing after harvest, the losses can be significantly higher. Therefore, resistance to disease and toxin accumulation is a central problem for which research is only in the initial stages of finding solutions.

The presence of FHB resistance has been proven on a wide scale, but no immunity was found until the researchers in [1,5,6,7,8,9] described it in terms of visible symptoms, DON, and yield reduction. The first highly resistant spring wheat variety was Sumai 3, registered in China in 1973 [10], and its resistance has remained stable globally since that time. *Fusarium*-damaged kernels have been known about for the past 100 years [11]. However, scabby grains were mentioned as a possible resistance trait in 1963 [12], and 30 years later, this trait was introduced into the breeding methodology [13,14]. DON was first detected in wheat in 1983 [15]. It is not an accident that its role in plant breeding became significant only years later.

FHB is caused by a high number of *Fusarium* spp. and produce a high number of mycotoxins [16,17]. We do not have enough data to prove that the resistance in wheat to the different *Fusarium* spp. is the same against all species, except for in *F. graminearum* and *F. culmorum* [13,14,18]. The significance of deoxynivalenol (DON) is that it occurs worldwide and has been identified in biological samples from humans and animals as a component of multi-toxin mixes [19,20,21]. The multi-toxin problem can be addressed in the future, but in this study, only DON will be analyzed.

The only condition that is generally accepted is that the susceptibility of the varieties is the ultimate cause of epidemics, and DON contamination often significantly surpasses the limit values. The conclusion is that we need more resistant cultivars, as this increases the efficacy of fungicide treatment [22]. Resistance is race-non-specific, but the relationship between DON content and visual symptoms is variable and therefore needs experimental proof [18,23]. The data have often shown genotype–isolate and isolate–year interactions, which influence the ranking and estimation of resistance levels [13,14,24]. Fuentes et al. [25] are among the few researchers to report on stability by comparing 14 cultivars, with colonization of the spike, FDK, and DON being chosen. The researchers concluded that the stability of variety responses could be described, but that “using more than three replications or scoring more than 10 spikes per plot had little practical value in characterizing FHB reaction”. For the stability analyses, four different tests were used, including the Eberhart and Russel [26] method.

Many studies discuss FHB resistance (traditional and molecular genetics), but only a minority (10%) of the screened studies present FDK or DON data [27,28,29], and even fewer focus on both. Therefore, in terms of breeding, the relationship between resistance and toxin regulation remains unknown [12,13]; in addition to these problems, new problems also arise that need to be solved [30]. As the data originate from only a few samples [28,29], they are not reliable enough for the sufficient evaluation of stability and adaptation ability. The idea of whether many thousands of DON datasets from different laboratories could be used has been discussed, but while they are useful for trade, they are not comparable and no infection data beyond these are known. Our conclusion is that useful data concerning resistance to disease and resistance to toxin accumulation can only be gained from well-planned artificial inoculation studies. This is the only way to compare resistance to toxin accumulation and disease resistance and identify toxin overproduction, relative DON resistance, toxin contamination, and many other factors. These studies would be able to show the functions of resistance QTLs in toxin regulation, and only in this way will we be able to detect QTLs that regulate DI, FDK, and DON alone or in combination [27,31].

The stability tests in variety testing are normally used to demonstrate yielding and adaptation ability. In genotype testing, yielding ability is the most widely screened trait. In this study, we adopted the method described by Eberhart and Russel [26]. These authors compared the mean performance of a variety across years and locations, and the individual varieties were compared to this mean performance. When the b value (stability index, SI) is lower than 1 in the linear function y = a + bx, this means increasing stability from close to 1 to close to 0 (the best case), and values higher than 1 indicate increasing instability. The first application of this stability test for FHB was published in 1995 [13], and then in later studies [14,24]. The data prove that varieties and genotypes strongly differ in their stability indices. In recent decades, this aspect of FHB research has seldom been mentioned. However, it has since become a significant problem for several reasons. For example, Fuentes et al. [25] demonstrated that the standard one inoculum over 2–3 years does not provide enough reliable data to correctly calculate the stability index. Variance is generally used in statistics, but has not been used to estimate stability. As it is easy to calculate, we opted to test it using one-way ANOVA.

Mesterhazy et al. [32] reported on the stability of fungicide responses; the most effective approach was found to be the combination of moderate resistance and fungicide. At greater resistance, the efficacy of the fungicide control is better. Lana et al. [33] found that more resistant maize hybrids exhibited a more stable DON response. Over a period of 20 years, Gaire et al. [34] identified slow development in FHB resistance, considering visible symptoms, FDK, and DON; however, the use of known QTLs has seen very little change. During screening, genotypes with better resistance and without known QTLs have been identified, including in Hungary [35]. Yan et al. [36] tested Chinese wheat genotypes in the Yangtze valley and several higher-resistance genotypes were identified for both DON and nivalenol. These results are important evidence supporting the argument for variety registration for FHB resistance and DON, as valuable genotypes can be detected, susceptible DON producers can be excluded from production, and the necessity of high stability can be stressed.

In disease resistance against rusts, the stability for the most effective resistance genes (less than 10% of the identified genes) providing immunity is 100%, at least until a new race emerges. The popularity of these genes in breeding remains very high. However, many of the specific monogenes induce partial resistance of varying degrees [37,38,39,40,41], which enables disease development of varying rates. Non-specific QTLs were identified as working against yellow and leaf rust [42,43,44,45,46]. For these, stability information would be necessary. This is similar to the issue we have in Fusarium. No physiological specialization is known, and no immunity has been identified until now. Chemical control was found to be much less successful against Fusarium than in the control of rusts. Resistance to FHB is polygenic; even 5–15 QTLs can cooperate to ensure the resistance of a variety or breeding line [27,31,47,48]. Such cases have variable results, and the resistance is partial. Additionally, QTLs have different functions and different influences on the most important traits (DI, FDK, and DON) [31] and require a different approach compared to the monogenetically inherited resistance traits. As large and significant differences occur in the breeding process, a simple screening can help to significantly reduce food safety risks [13,14,18,27,49,50,51]. As Fusarium resistance is not an easy subject, it is unsurprising that very few relevant studies on the problem of stability have been found [25,29,52].

What should be measured? Fusarium tests use two basic methodologies and their variants: the first examines the resistance to spreading following point inoculation, and the second is the spray inoculation method suggested by Schroeder and Christensen [12] to measure resistance to invasion. We have demonstrated [30,52] that point inoculation determines the spreading factor of FHB DI resistance (*Fhb1* QTL) by securing only about 50% of the total resistance (this agrees well with the findings of Liu and Wang [10]). Type 1 determines the other 50%; moreover, the results of spray inoculation simultaneously detect Type 1 and Type 2 resistance and, thus, the total resistance capacity of the plants. For this reason, we used only spray inoculation. With this methodology, the whole genetic background can be measured. For breeding, resistance evaluation, and variety registration, this seems to be the most suitable assay for identifying whatever resistance may occur in our plants to FHB. The second trait is FDK (rate of visual estimation of scabby grains), and the yield of the sprayed heads secures enough grain for DON measurements [29,30]. The expression of resistance heavily depends on ecological conditions; therefore, as well as more isolates (inocula), more years and locations are necessary to secure the database [24,35] for stability analyses.

It is known from the work of Wollenweber and Reinking [53] and Booth [54] that the aggressiveness of the different isolates differs. This aggressiveness is not stable and is usually lost over time. A passage may restore this aggressiveness, but a change in the isolate will eventually be necessary [24]. Except for a number of cases, there is no aggressiveness control in experimental praxis; only conidium concentration is applied, but not for the regulation of aggressiveness. This is also valid for the mixing, diluting, and increase in conidium concentration; therefore, this may be a source of false phenotyping [14,24,29,52].

In the international literature, we did not find any studies that presented a larger number of genotypes tested on more than 20 different epidemiologic cases that would be suitable for a comparison of the stability test methodologies. Our three earlier tests [14,25] and an unpublished experiment provided datasets for performing this analysis.

The objectives of this study were (1) to conduct a retrospective analysis of stability, using data from the recently tested Hungarian genotypes for all three traits; (2) to determine which stability calculation method seems to be more reliable and whether the stabilities for DI, FDK, and DON are similar or divergent for the three traits; (3) to conduct principal component analyses for all experiments (all traits and tests were supported by stability analyses of the traits—both separately and together—using two different procedures to help develop a more comprehensive understanding of the background of stability); (4) to perform correlation studies with the aim of determining how the correlations are modified by the epidemic situations; (5) to evaluate how stability analyses may contribute to a significantly better methodology for resistance testing, phenotyping, and variety registration.

## 2. Results

### 2.1. Experiment Conducted from 1990 to 1993 [15]

#### 2.1.1. FHB Traits and Their Relationships

The basic data for the disease index (Table S1) show nearly the same regression lines and correlation coefficients for stability and variance. The highest stability is 0.39, the highest instability is 1.75, and for variance, the values are between 42 and 630. The data achieve nearly full agreement for each genotype (linear r = 9682; polynomial: 0.9592), indicating similar values for the counting stability (Figure S1A). The DI means relate similarly to the variance and stability indices, but the variance shows somewhat lower R^2^ values. In both cases, we observed closer relationships among the polynomial functions, indicating a lower variance for the most sensitive genotypes (Figure S1B,C). The highest unstable response was 1.15 (Figure S1D) and the most stable genotype had a response of 0.3966 (Figure S1E). The difference is approximately threefold. The genotypes were not preselected, and they showed natural variability in an unselected population. In spite of this, the differences are considerable and highly reliable.

Using the data presented in Table S1, we conducted a rank analysis; the results of this are shown in Table 1. The ranking was conducted separately for each variety. All varieties had rankings between 1 and 31. The means varied between 2.8 and 27.3, exhibiting a highly significant tenfold difference. Variance was ranked between 3.5 and 42.8. Ten epidemics had extreme high variance values of 31, and there were cases for which genotypes with the same mean had a fourfold difference in variance. Four isolates differed considerably in terms of mean ranking, while the others were similar to one another. The ranking of some cases varied in one epidemic between 2 and 22 (Fc12551), 2 and 29 (Fg12206), 6 and 25 (Fv12377), and 11 and 31 (Fc12375). This means that none of the isolates can be said to have presented a reliable picture of the resistance level or the rankings of the genotypes. Nevertheless, an observable pattern emerged: the correlations between genotypes with 31 epidemic situations were all significant at *p* = 5% or higher, varying between r = 0.401 and 0.931. The correlation means for a given genotype differed between r = 0.683 and r = 0.816. There were epidemics where the ranking was more uniform: 12216 in the first-year ranking between 1 and 6, in the second year between 3 and 18, and in the third year between 2 and 29. The value of 89.4 in the third year indicated good stability with rank differences between 22 and 31 with a variance of 9.6. For the other traits and experiments, the rankings are not presented in detail, but the findings were the same.

The values in the variance data are low when the aggressiveness values are low or high; the highest values can be seen at the intermediate aggressiveness levels. However, this is not a general rule. The lesson is clear: we need more epidemic levels in order to obtain more reliable data regarding the resistance level and its stability.

The correlation analysis between the variety responses across epidemic situations revealed very close and highly significant relationships. The mean of the 190 correlations was r = 0.6755, and only 20 were not significant. The situation was very different when we compared the epidemic responses to the different isolates and years (*n* = 31) (Table S2). Of 465 correlations, 112 were higher than r = 0.5640 (*p* = 0.001), 44 were significantly higher than r = 0.4570 (*p* = 0.01), and 39 were higher than r = 0.3570 (*p* = 0.05). Altogether, 195 showed significance at different levels, but 270 were not significant. With the different responses of the genotypes to the different ecological conditions, it was determined that the variety rankings exhibit significant variation, and one isolate or even the same isolate in different years can yield different results. On the other hand, we see that the more aggressive isolates present a more reliable variety ranking and the amount of resistance can be measured with more precision.

The basic FDK data (Table S3) represent the original dataset. The comparison between FDK variance and stability data for the 20 genotypes shows a lower correlation than was found for DI, but the difference is not large. Only one genotype shows diverging responses to the two stability traits, Sgv-GT//Pred*Uhr (Figure S2A). The regression between FDK and stability is r = 0.74, and the variances were closer (r = 0.8563) than those registered for stability (Figure S2B,C). The most resistant genotype exhibited very good stability at 0.17, and the highest instability was 1.49. For variance, the data showed values between 0.32 and 1192; here, the variance indicated a closer relationship, and in both stability traits, the deviations were higher in both directions than those for DI (Figure S2D,E).

For the FDK correlations between the variety responses across different epidemic situations (31), the mean of the correlations was r = 0.7173; out of 190, only 5 were not significant. However, the responses were found to be similar to those of the different isolates in different years (epidemics); here, the picture is much more variable. Out of 496 correlations, 94 were significant at *p* = 0.001, 47 were significant at *p* = 0.01, and 42 showed significance at *p* = 0.05 (Table S4). This means that, in 313 cases, no significant relation could be detected, i.e., different epidemic situations influence the rankings of genotypes differently.

**Figure 1:**
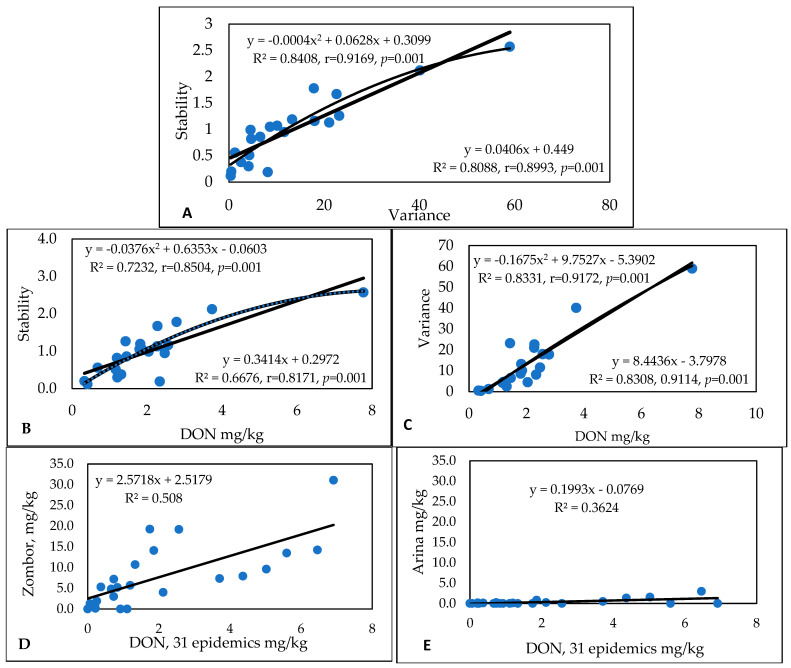
Comparison between the variance and stability for the 20 genotypes and DON contamination for variance and stability indices. (**A**) Comparison of the two stability traits. (**B**) Regression for DON mean and stability. (**C**) Regression between DON means and variance. (**D**) Very high instability, b = 2.57. (**E**) High resistance and stability, with a very low increase in function.

For DON (Table S5), the evaluation of stability and virulence (Figure 1A) provided nearly the same correlations (r = 0.9169 and r = 0.8993), and the two functions overlapped well with each other. The low variance (of about 5) indicated stability, with a “b” value of between 0.3 and 1. It seems that the polynomial function follows data points more precisely and closely than the linear function. It is similar, but not the same. The regression values for DON and stability are lower (Figure 1B), but the polynomial function also provides a more definitively different character (Figure 1C). For variance–DON regression, the linear and polynomial functions overlap with each other to a greater degree. The most unstable and most stable genotypes are present in the set; in Figure 1D,E, the larger font size of the b values indicates differences in stability. The difference is 14-fold. It seems that DON provides the strongest differentiation in relation to stability and instability.

The correlations between the DON data of the genotypes across epidemics showed an unusually low correlation in the mean (r = 0.3726), which is not shown here in detail. The correlation between the epidemic performance across the genotypes had a value of r = 0.3759, which shows that they are practically the same (Table S6). Out of 253 correlations, 42 were significant at *p* = 0.001, 20 were significant at *p* = 0.01, and 28 were significant at *p* = 0.05; in summary, 90 cases were significant and 163 were not significant.

A summary table for the three FHB traits is shown in Table 2; these data were subjected to a correlation analysis. For the correlations between the trait means, the stability means, and the variance values for the disease index, we can see that the numbers are nearly the same; however, for FDK and DON, the difference is larger, and SI exhibits lower values in some cases. Four genotypes (printed in bold) were identified as having good stability and good resistance: the Swiss Arina, two from the Netherlands, and one French line. The trait means show a strong medium status between DI and FDK, but they are lower between DI and DON. For correlation, the data for DI/FDK and DI/DON are not significant, but FDK/DON shows a significant relationship. For SI, the DI/FDK relation is just above the significant limit of *p* = 5%; the DI/DON relationship is not significant, and the closest correlations were found between FDK and DON. The highest correlations within the traits were found for DON. Table S7 shows the ranking data for their traits and means, and their variances and stability. Arina showed the best performance with rankings of only 1–3. These data are important because attention was paid to FDK and DON as well as head symptoms; this source has a very good general resistance against FHB for all of the important traits.

The correlations counted for the ranks (Table S7) were found to be similar to those we found for the original data, as shown in Table 2. The two stability datasets show similarities for DI, but for FDK and DON, there are more differences. The variance had a closer relationship with the data here than with the SI data. The DI/FDK correlation had a value of r = 0.70; the DI/DON correlation had a value of only r = 0.497; and the closest correlation was found to be the FDK/DON relationship. A similar correlation was found for the variance, and the weakest correlation was found for SI. The lowest correlations were found between DI and the other data in the first three columns. In FDK and DON, all correlations had significantly closer relationships, indicating their explicit role in DON determination. We should note here that we assessed genotypes such as Mon-Ar, which is highly susceptible according to the disease index, but it performed significantly better in relation to FDK and DON. Conversely, 85–92 was found to be resistant to disease, but it was much more susceptible to FDK and very sensitive to DON accumulation. Renan had a good resistance to disease, but was much more susceptible to DON accumulation. Zombor ranked 20th for all traits, but this was an exceptional case.

**Figure 2:**
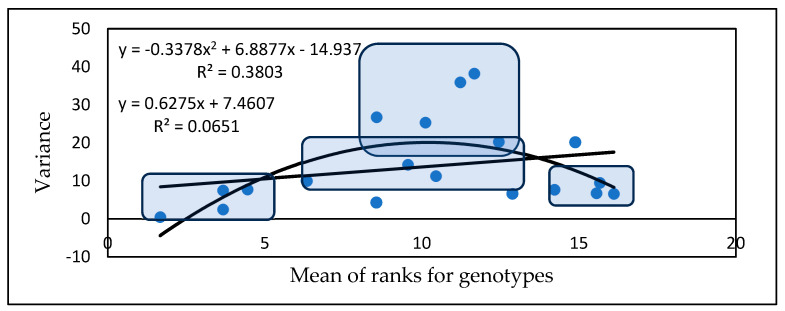
Rankings and their stability in the summary responses of the genotypes (1990–1993).

The genotypes can be grouped based on all traits. As previously mentioned, there are four groups, the most useful of which are those that have low numbers and high stability for all traits. The next group has medium rankings with variance values, and the third group has medium severity, but very high instability (variance). The members of the fourth group exhibited high stability in their susceptibility behavior (Figure 2). One genotype could not be clearly classified.

#### 2.1.2. Influence of Isolates on the Variety Ranking of Genotypes in Experiment 1

**Figure 3:**
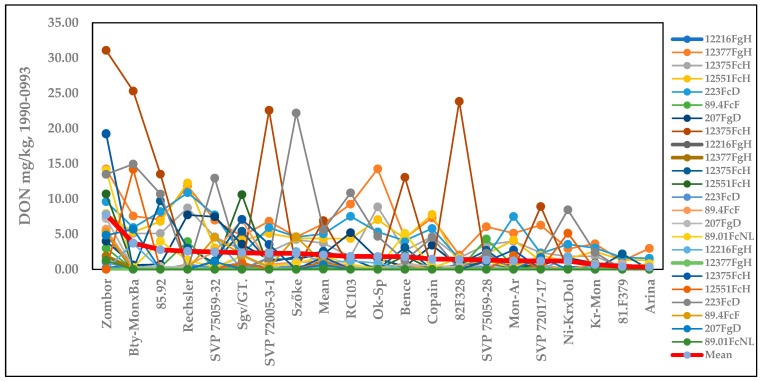
Response of winter wheat genotypes to DON (deoxynivalenol) contaminations in 31 epidemic situations. The thick red line indicates the means of the 31 situations.

It is important to provide visually demonstrative information on the resistance expression of the genotypes under different epidemic conditions. Figure S3 presents the DI data for all 31 epidemic situations; the thick red line shows the averages of the epidemic data. It is very clear that genotypes that are very close to each other in their means reflect very different data for the different isolates. It is also clear that there are epidemics for which the infection severity (DI) is very low, and for which differentiation between the genotypes is poor or not possible. Another important aspect to note is that more resistant genotypes have much lower maximum values in comparison with highly susceptible genotypes. For DI, the most susceptible genotypes performed slightly better. In resistance tests, we should not only determine the rankings, but should also describe the amount of resistance where possible. This is not possible when there is one isolate with a two-year test. Similar findings were obtained for FDK (Figure S4) and DON (Figure 3). However, the means of the isolates better reflect the amount and rankings of resistance.

### 2.2. Experiment Conducted from 1994 to 1996 (Mesterhazy et al., 1999) [15]

#### 2.2.1. FHB Traits and Their Relations

The original data for DI (Table S8) show wide variability in response to different epidemic situations. In this set, genotypes from the Szeged FHB breeding program were tested several times, with very low infection severity. The genotype means for DI vary between 2.61 (Sgv/NB//MM/Sum 3, containing both Sumai 3 and Nobeoka Bozu in the pedigree) and 38.82 (Csaba), reflecting a roughly 15-fold difference. The most stable genotype had a variance value of 4, with the most unstable presenting with a variance of 839, reflecting a nearly 200-fold difference. The SI varied between 0.10 and 1.90, reflecting a 20-fold difference. The variance and SI (Figure S5A) show a very close correlation, and there is almost no difference between the linear and polynomial functions. The disease index correlated more closely with variance (Figure S5B) than with the stability index (Figure S5C); however, the linear and polynomial approaches showed only small differences. In Figure S5D, the instability of the most susceptible genotype, Csaba, is shown to be 1.90 (Figure S5E). The most resistant genotype, the cross Sgv/NB//MM/Sum3, demonstrates minimal differences between the stability and instability indices.

The DI rankings (Table S9) present the data of 24 epidemic situations for 25 genotypes. The situation is similar to that in the previous experiment conducted in the period 1990–1993. In Table S9, we also present the means of the original data for the isolates and the genotypes. The data were ordered by cultivar, ranging from the most susceptible on the left to the most resistant on the right. The reason for this is that we can compare the two genotypes with nearly similar data; however, with the exception of similar values of 10 or higher, ranking differences occur for different isolates in both directions. At lower aggressiveness levels, the rankings are closer and the variances are lower. At high aggressiveness levels, the tendency is similar, although rather large differences can occur. In line 11, differences between the rankings of 7 and 24 can be found. The variances are quite different both horizontally and vertically, allowing for the analysis for genotype and epidemic positions. For example, Jbj50, a susceptible cultivar (cv) for the isolation of 44FcA, has a ranking of 4, while the isolate 207FgD has a ranking of 22. Low and high variances can occur anywhere. It is also important to note that isolates in different years can have rather different performances; for example, 207 FgD exhibits weak performance one year and strong performance the next, but in the third year, the aggressiveness is very strong (line 21). When using such a large dataset, the estimation of resistance risk is much more reliable than it is in cases using small datasets. Of note is that the correlations between genotypes and different isolates vary between r = 0.181 and r = 0.972, with a mean of r = 0.70.

The correlations between the data of individual genotypes and means are between r = 0.507 and r = 0.953; the mean relationship is r = 0.696. Wide variability in resistance was found (0.92–50.2%). However, when the correlations are counted for isolates across both epidemics and genotypes, the outcomes are different (Table S10); here, the mean is r = 0.56, which is lower than expected, but this is still higher than in the first experiment. Of the 276 correlations, 106 were significant at *p* = 0.001, 34 were significant at *p* = 0.01, and 37 were significant at *p* = 0.05; a total of 101 were not significant, but the conclusion is similar. Here, the data on one isolate and 2–3 years of data are certainly insufficient for use in characterizing rankings and determining the amount of resistance.

The FDK data (Table S11) show, again, a high level of variability: the most resistant had values of 0.92 and the most susceptible had values of 50.43% across the 24 epidemic situations. The mean aggressiveness of the isolates was between 2.35% and 52.85%. When evaluating the data of the three inocula of an isolate in different years, we found four isolates—Fc12375 (1), Fg12377 (1), Fc207 (2), and Fg44 (1)—with 1–2 cases with lower means in comparison with half of the general mean. In these cases, the differentiation power of the inocula was less strongly expressed. For the other four isolates, the three inocula of a given isolate were above average. The regression between variance and stability (Figure S6A) was closer according to the polynomial function. The regressions for FDK and variance (Figure S6B) were very close, but in the stability index, the two functions resulted in larger differences, with the linear version being less close (Figure S6C). The polynomial function had a much better fit with the data. The most sensitive genotype, Zugoly (Figure S6D), had an SI of 1.4, but this value was obtained from the linear model. For the polynomial model, the correlation was much closer, with a b value of 3.92 for this function. For this reason, it can be concluded that there is a problem with the values of the nonlinear functions. The most resistant genotype is extremely stable, with an SI of only 0.04 and a 35-fold difference (Figure S6E). A scientific problem that we detected must be addressed, but this requires further research.

Regarding the correlations between the FDK genotype responses under the conditions of different epidemic situations, we counted 300 correlations: 166 were significant at different levels and, in 134 cases, no significant relationship was documented. The mean of the 300 correlations was r = 0.459, and a number of genotypes were found to be significant at *p* = 0.001. The differences in resistance between the means varied between 0.92 and 50.43. For the 276 correlations between the tested epidemics (Table S12), the mean was r = 0.441; here, 138 were significant and 138 were not. The correlation mean was r = 0.441, which is very close to that of the correlations found for the genotypes; there was almost no difference between the two means.

**Figure 4:**
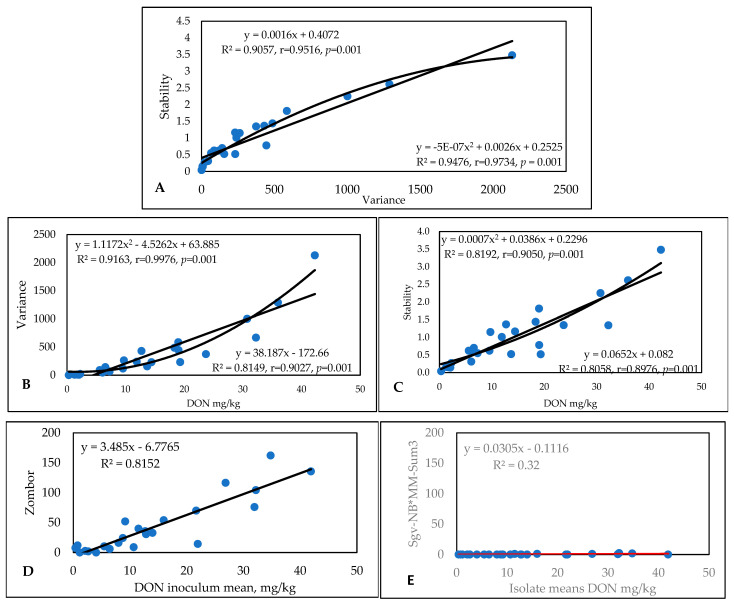
Comparison between the variance and stability for the 20 genotypes and DON contamination for variance and stability indices. (**A**) Comparison of the two stability traits. (**B**) Regression for DON mean and stability. (**C**) Regression between DON means and variance. Unstable responses during different epidemic situations; a forecast for response is not possible (**D**). Highly stable genotype (**E**) containing Sumai 3 and Nobeoka Bozu pedigree; in all epidemic situations, the DON contamination is zero (below the detection limit) or slightly higher.

The DON data (Table S13) again showed large genotype differences, with values between 0.32 and 42.25 mg/kg across the 24 epidemic situations. The lowest concentration was 0 (not detected), and the highest was 162 mg/kg. The variance and stability correlate well (Figure 4A), with both the linear and polynomial functions being very close to each other. The DON/variance regression was much higher in the polynomial function, and the linear function was lower (Figure 4B). The isolate means and stability provide a more diffuse picture, with lower correlations being found in the polynomial functions (Figure 4C). We present the stability linear functions for the most susceptible genotype, Zombor (Figure 4D), and for the most resistant line from our breeding program, Sgv-NB/MM-Sum3 (Figure 4E). The SI for Zombor is 3.48, and for the resistant line, this value is only 0.0305. For the very resistant lines, the chance is much lower that a low–medium level of DON contamination will be found. This is a good reason to select resistant and highly resistant wheat genotypes for commercial production. In this way, superior genotypes can be identified with a much higher probability. The most stable and most resistant genotype was the line Sgv-NB/MM-Sum3, from our breeding program (Figure 4E). There is a difference of more than 100-fold between Zombor and this resistant line.

The correlations for DON production (*n* = 25) between the 20 genotypes (Table S14) showed significant relationships with others observed in other tests, but 5 of the genotypes showed only a few significant relationships with the other 20 cultivars. The general mean of the correlations was r = 0.500. Out of the 300 correlations (mean = 0.500), 111 were significant at *p* = 0.001, 53 were significant at *p* = 0.01, 34 were significant at *p* = 0.05, and 102 were not significant. The correlations with epidemics again show an unusual picture: the correlation mean is r = 0.469, which is only slightly worse than the genotype mean. In Table S15, the correlations with the data from 1995 are shown; here, the number of significant cases is much lower than we expected, but several isolates show very good agreement with the 1995 data. Of the correlations, 81, 32, and 25 were significant at *p* = 0.001, *p* = 0.01, and *p* = 0.05, respectively, and 138 were not significant. The correlations between the data from 1995 and the other years gave much lower significances; most were not significant, but a significant year influence could be demonstrated.

The 1994–1996 data, accounting for the means and the two stability tests, are pooled in a summary including all of the traits. Ranking was conducted according to the DON contamination level (Table 3). As the traits were individually analyzed, here, the general picture is important: 6 of the 25 genotypes were ranked in the group as having all traits and indices below 50% of the median (dark and light green highlighting). In four cases, only the dark green designation was found with low values and high stabilities. Five genotypes were found with all indices above the mean (yellow/orange) and two belonged to the worst (orange) category. The LSD5% showed the high reliability of the data. Here, the correlation matrix is important. For DI, the relationship between stability and variance demonstrated no significant difference. For FDK, the variance was better, but for DON, no significant difference could be found. This means that, in this test, variance and SI are equally good for stability estimation. The variances for the different traits show correlations between r = 0.499 and r = 0.785. In comparing the two stability indices, disagreement can often be detected within and between traits.

The rankings clearly show the stability of the variety responses and provide a good overview of the situation (Table S16). Five genotypes were found with means lower than five; four of them had no higher rankings for any trait than all five—only Ringo Star had two ranks of eight. For these, the variance was lower than 1. Only three genotypes had lower means than 10, but in each, we had one or more cases with higher ranks up to 12, 19, and 14. Góbé, Siouxland, 78.1.4, Kende, and Szőke had very high variances (high instability) in a medium range. Disagreement between variance and SI was identified within traits and between traits. The correlations between variance and stability with the traits were closer than those of the original data. In this experiment, the three traits had several cases, such as 81.60/NB-Kő, that ranked medium in terms of ear symptoms and performed much better for FDK and DON. Siouxland had good resistance to visible symptoms, but it had significantly higher ranks for the other two traits. Sum3-81-60 had good resistance to DI and FDK, but was more susceptible to DON. Hence, good agreement is an exception.

**Figure 5:**
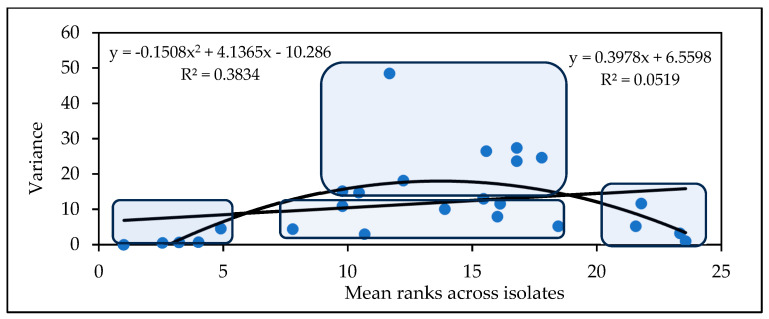
Regression between means of ranks across isolates and stability indices and years for 2009–2012. Genotype groups: lower left—good resistance with high stability; lower middle—medium resistance, good stability; lower right—high susceptibility, good stability; upper middle—medium resistance and high instability.

The variety means and variances (Figure 5) can be used divide the genotypes into four groups. Five genotypes belong to the resistant and low-variance group, and four genotypes belong to the highly susceptible and low-variance group. In the middle, we have a group ranking between 7 and 18 on a linear pattern and below, with the same mean performances as those achieved by genotypes with highly variable performances. These results are about the same as those obtained in the first experiment conducted in the period 1990–1993.

#### 2.2.2. Influence of Isolates on the Variety Ranking of Genotypes for Experiment 2

**Figure 6:**
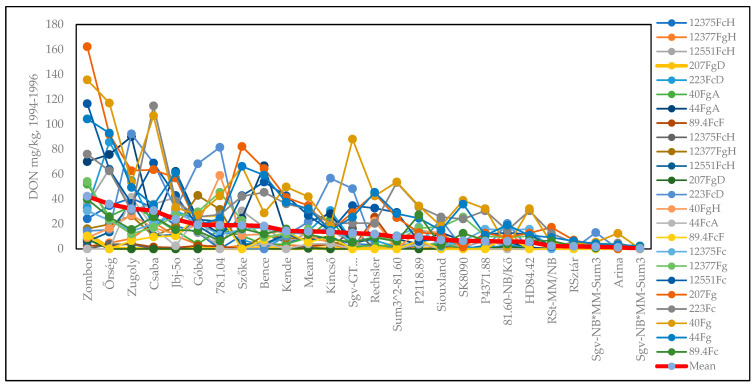
Winter wheat genotype responses to 32 epidemic situations, 1994–1996.

Figure S7 presents the genotypes’ reactions to the different isolates for the disease index (%) of visibly infected spikelets. It is clear that the highly susceptible genotypes show the greatest differences in DI. The variability in the responses is much closer for the high-resistance group. The means show the real variety differences. Figure S8 shows the FDK data, presenting the same picture. Again, the red line is the best indication of the amount of resistance that is present, and the ranking is much more reliable than any of the 24 epidemic situations. For DON, the same result is evident (Figure 6).

### 2.3. Experiment Conducted Between 2009 and 2012 [14]

#### 2.3.1. FHB Traits and Their Relations

The disease index (Table S17) showed high variability in the resistance of 40 wheat genotypes under the conditions of 36 epidemic situations; the level was determined to be between 3.42 and 18.26 percent. The variability in the severity of the 36 epidemics was between 0 and 39.2; this variability is large enough to obtain a picture of the differential responses of the genotypes under different epidemic conditions. Figure S9A shows the regression between the two stability traits: variance and b value. The two regression curves (linear and polynomial) overlap each other well; here, the polynomial was slightly better adapted to the data matrix. The genotype rank mean shows closer correlations with the variance data for both function types (Figure S9B) in comparison with the rank means and stability indices (Figure S9C). The variance here seems more reliable. The most susceptible and resistant genotypes show very unstable (Figure S9D) and very stable (Figure S9E) behaviors under the same ecological conditions.

A correlation analysis of the 40 genotypes during 36 epidemics produced 780 correlations; their mean is r = 0.70 and the LSD5% limit is 0.32. Most were significant at *p* = 0.001, and only nine were not significant. This means that, with this amount of data, the genotype data correlate well and seem reliable, as the means exhibit reliable results across many epidemics. The situation differs for epidemics across genotypes. Out of the 613 correlations, 179 are significant at *p* = 0.001, 80 are significant at *p* = 0.01, and 68 are significant at *p* = 0.05 (Table S18). The correlation mean is only r = 0.35 and 286 are not significant.

For FDK (Table S19), the data reveal high resistance differences between 0.91 and 22.04%, and the epidemic severities differ between 0.2 and 62.1. The stability and variance for the genotype means exhibit very similar correlations, with r = 0.8862 and r = 0.8916 for the linear and polynomial functions, respectively (Figure S10A). These results are very similar to the ones we measured earlier. This observation is also valid for the FDK mean and variance regression, where r = 0.9120 and r = 0.8916 are the corresponding correlations (Figure S10B). However, the FDK mean and stability regression have lower correlations; here, the correlation for linear regression is significantly lower, while the polynomial regression has a better fit with the plotted data (Figure S10C). The most susceptible (Figure S10D) and most resistant (Figure S10E) genotypes here show high instability and stability for the susceptible and resistant genotypes.

The correlations between 40 genotypes across isolates had high values; the mean of the 630 correlations was r = 0.74. Out of 780 correlations, 537 were significant at *p* = 0.001, 48 were significant at *p* = 0.01, and 33 were significant at *p* = 0.05. Only 12 correlations were not significant. This means that the large dataset could provide a reliable measuring system. The number of correlations from the epidemic cases (36) across the cultivars proved the opposite. Out of 630 correlations, 87, 55, and 77 were significant at *p* = 0.001, *p* = 0.001, and *p* = 0.05, respectively, and 411 were not significant (Table S20). This means that the individual epidemic situations had different data matrices, proving the need for a solid database of reliable genotype data.

**Figure 7:**
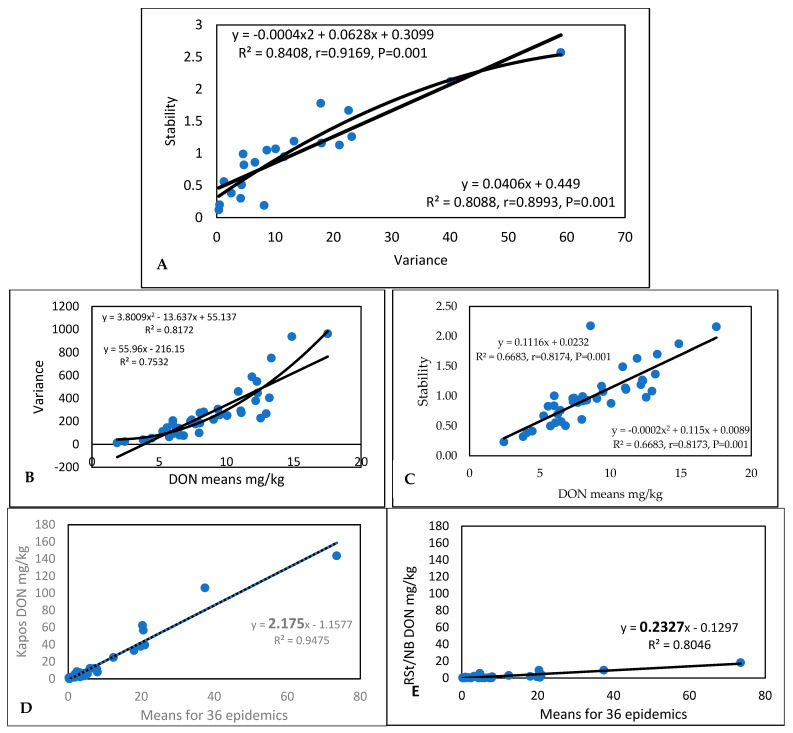
Resistance testing of 40 genotypes in 36 epidemic situations for stability according to variance and stability indices of DON contamination (**A**). Stability data are given for the relationship between genotype resistance and stability traits in (**B**,**C**), but here, the stability data follow a more polynomial trend. The highly susceptible cv genotype (**D**) showed high variability and instability in SI, but the more resistant genotype provided much lower variability and higher stability in its performance (**E**) (NB = Nobeoka Bozu) (2008–2012).

The original DON data (Table S21) show wide variability in resistance to DON accumulation and in the aggressiveness of the different isolates in different years. The most resistant line had a mean DON contamination of 1.83 mg/kg, with the most susceptible having a mean performance of 17.32 mg/kg. The individual data varied between 0.27 and 73.52 mg/kg, with a mean performance of 8.59 mg/kg. Figure 7A presents the two stability traits that give the same correlation coefficients; with one or two exceptions out of a total of 40, these traits exhibit very similar results. In terms of the correlations between mean DON performances and stability traits, we see higher correlations for the variances and r values for the stability index (Figure 7B,C). It is interesting that, for the variance/DON regression, we observe significantly closer r values for the polynomial function than for the linear function (with a tiny difference: DON data regression provided significantly looser correlations, but the two functions resulted in the same number). The most susceptible and unstable genotype was Kapos, with an SI of 2.17 (Figure 7D), presenting very high toxin contamination in most cases. However, this value is much lower than that obtained for the Zombor genotype in the 1994–1996 test, with its value of 3.48 indicating extremely high instability. The most resistant genotype, RSt/Nobeoka Bozu (RSt/Nb) (Figure 7E), has a stability index of 0.23, which is 10-fold lower than that of Kapos (SI = 2.15). This indicates its inability to adopt to different epidemic stresses.

The correlations between the genotypes across the 36 epidemics provided 780 correlations, with a mean value of r = 0.861. All correlations were significant. This means that the isolate means provide a good picture of the resistance of the given cultivar or line. The correlations between epidemics across genotypes show a similar picture to those presented earlier. Out of 630 correlations, 65 were significant at *p* = 0.001, 79 were significant at *p* = 0.01, and 131 were significant at *p* = 0.05, while 355 were not significant (Table S22, from data presented in Table S17).

The summary data for the three traits (Table 4) show mixed genotype patterns. The best genotypes with all traits are shown in a dark green color. Such genotypes for all traits were not found. We identified 9 genotypes out of 40 where all of the traits are under the average, indicated using dark and light green in the genotype designation. In addition to the high resistance to DI, the FDK and DON are similarly low and the stability indices are also low, e.g., the stability is high. For DI and FDK, two have a yellow designation, but their high toxin stability and low DON content make them important. The Balaton genotype is in the green area for DON, but it has a higher susceptibility to DI and FDK. GK Feny and GK Csillag were much more susceptible to DI, but had better performances for FDK and were found to be significantly less susceptible to DON. These are examples for cases where, with higher DI susceptibility, we can identify useful genotypes for production under epidemic conditions. We should stress that the GK Csillag and GK Feny genotypes underwent fungicide testing and their fungicide resistances were excellent; additionally, DON contamination dropped below the limit of 1.25 mg/kg. The GK Elet genotype was the most susceptible to head symptoms, but had only 50% DON susceptibility compared to the moderately susceptible SzD 6515, which had 50% less DI susceptibility and significantly higher DON contamination. On the other hand, some genotypes were identified as having moderate resistance to FHB but much higher susceptibility to DON contamination; these are toxin overproducers, such as GK Hunyad, SzD 18364, and SzD 5205. In addition, genetic regulation diversity can be assumed here as proven. The highest DON producer, GK Kapos, provides only a medium DI, but for FDK, it ranks at the top. The means of the 36 epidemics support the resistance level and resistance ranking well for genetic research to make deductions to test the genetic background of genotypes. Variance and stability often differ based on both the original data and the rankings. Even when similarities occur, we have to decide which will be better for the evaluation of the experiments. We posit that both the original data and the rankings are significant for making a correct evaluation.

For the rankings, seven genotypes were identified that had data below the average (Table S23). The results were similar to those from the original data, but with certain differences. This was a stricter constraint, imposed because low variance and high stability (=low b value) are important. Examining the genotypes shows that the two stability indices often differ (see the color differences), and the different traits often show divergent behavior for the stability indices.

The correlations between DI and stability data differ, while the relationship with the variance data is much closer. The difference is large. For FDK, the two values are closer, but the variance provided evidence for a closer relationship with DI. For DON, the two correlations are nearly identical. This means that the variance seems to be more useful in describing stability in comparison with SI. The DI data exhibited poor correlations (below r = 0.50) with FDK and DON; the FDK/DON relation was much closer, with a value of r = 0.820, thus supporting earlier findings. The variances in different traits were somewhat higher for FDK, but there were no significant differences between the DI/DON and FDK/DON correlations. For stability, a significant correlation was found for FDK/DON only; the others showed no significant correlations. This, again, supports the claim for the higher significance of the FDK/DON connection.

**Figure 8:**
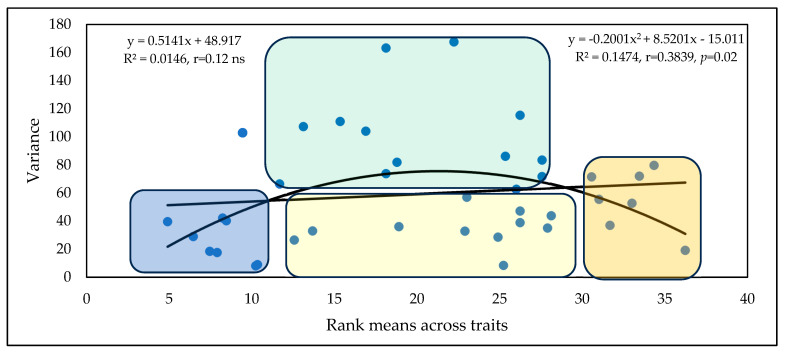
Classification of the 40 genotypes based on mean rank positions for DI, FDK, and DON, for 2009–2012. Blue indicates low variance and excellent ranking position; light yellow indicates medium ranking with low variance; light green indicates medium position with no stability; orange indicates high ranking (susceptibility), with a stable likelihood of the occurrence of epidemics. In the middle categories, such epidemics are less frequent.

Based on the means of the rankings, four genotype groups can be identified (Figure 8). The first group contains eight genotypes that have low rankings and low variance, e.g., high stability. These genotypes are preferred as they are more resistant and stable, and form the group with the greatest potential for successful application. In the second group, we have the medial class, with variances below the regression line and with medium positions in the ranks (11 genotypes). Here, the variance can be low, but genotypes with excellent fungicide affinities could be used when this is tested accordingly. The third group contains genotypes in a medium position with high variance, which should be excluded from any further considerations (15 genotypes). The last group comprises seven genotypes with higher stability, but high susceptibility in all traits. These should also be discarded.

#### 2.3.2. Influence of Isolates on the Variety Ranking of Genotypes in the 3rd Experiment

**Figure 9:**
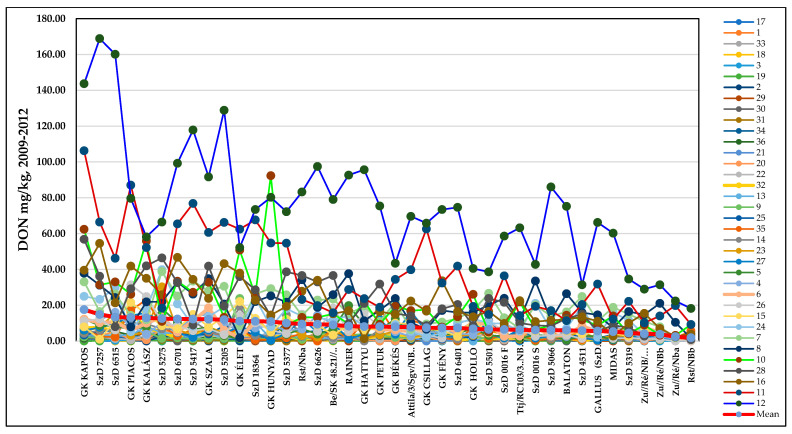
DON mg/kg of 40 winter wheat genotypes in 36 epidemics (four isolates, three methods, and four years) from 2009 to 2012 [24].

Figure S11 presents the genotypes’ reactions to the 36 epidemics (isolates, methods, and years for disease index), showing the percentage of visibly infected spikelets in relation to the total. It is unsurprising that the highly susceptible genotypes show the greatest differences in DI responses. The variability in the responses is much lower for the group with the highest resistance. The means show the real variety and differences in the results. Figure S12 presents the FDK data, reflecting the same picture. Again, the red line is the best representation of the amount of resistance; the ranking is much more reliable here than for any of the 36 epidemic situations. The same is observed for the DON epidemics (Figure 9). As the rankings differ between traits, all three are necessary to evaluate the real values of the genotype and variety.

### 2.4. Experiment 4: 2019–2021

In this experiment, we reached a point in our research that was closer to practical breeding work. This did not influence the inoculation of the genotypes against FDK; the evaluation for this corresponded to the first three experiments. For DON, the grains of the harvested and threshed yield were pooled for the *F. graminearum* isolate and its mixture with another Fg. isolate, and the same was made with the *F. culmorum* isolate, and its mixture with another *F. culmorum* isolate (see Section 4); thus, the pooled grain was the subject of the toxin analyses. In a breeding program, it is possible to take a simplified approach; however, for scientific research and variety registration, which require as much data as possible, this would not be the most appropriate approach.

In this experiment, 15 commercially available varieties were tested over three years. Table S24 presents the DI data. All cultivars originated from a breeding program where Fusarium resistance was not the main breeding task. Among these cultivars, several were identified as having moderate resistance and susceptibility. Most have long production periods (5–15 years); additionally, during the epidemic years, the control variety GK Csillag was used as the one fungicide treatment grain under the EU’s DON limit. GK Pilis and GK Megyer were significantly better, and the control, Genius, was also proven to be significantly better than GK Csillag. This field information is important because the artificial inoculation tests cannot be used alone to determine the limits of resistance. The lowest variance was found for GK Csanád, GK Pilis, and GK Megyer. As we expected, variance showed a stronger correlation with DI, and the polynomial curves fitted better in both cases. The variance and SI had similar correlations (Figure S13A). In terms of the regression between the disease index and the variance, the polynomial function gave a better result (Figure S13B). The regression between DI and SI was much weaker, but the correlations were still significant (Figure S13C). One susceptible genotype showed a strong increase in linear function (GK Békés, Figure S13D); the most resistant genotype achieved an SI value of 0.42, less than the limit of 1, indicating better adaptation (GK Pilis, Figure S13E).

The FDK data (Table S25) of the genotype set presented highly significant differences. Altigo, a control variety, was the most susceptible (22.8%), and GK Bakony was the least infected (5.2%). Three Szeged varieties (including GK Pilis), together with Genius, are also on the list with low FDK severity values of 7.1 and 7.3%. The variances for the most resistant genotypes are low at 59.9 and 73.5. The variance in the most susceptible genotypes shows high instability above values of 300 and 400. The variance also differs between years and depending on the inocula used. The inocula from the same isolates and mixtures also lead to changes in variance. The variance and stability data correlate very well in both the linear and polynomial functions (Figure S14A), and the correlation between the variance and stability data for FDK is very close (Figure S14B,C). The high instability of the susceptible genotype is clear (Figure S14D); however, one special case is noted: Altigo was classified by a stable linear function and the data showed a clear polynomial function. This is proof that an exceptional nonlinear distribution makes the validity of the stability index from the linear function less useful. The characteristic linear distribution of data justifies the reliability of the high stability of the moderately or highly resistant genotype GK Bago (Figure S14E). The correlations are closer in comparison with those we found for DI.

**Figure 10:**
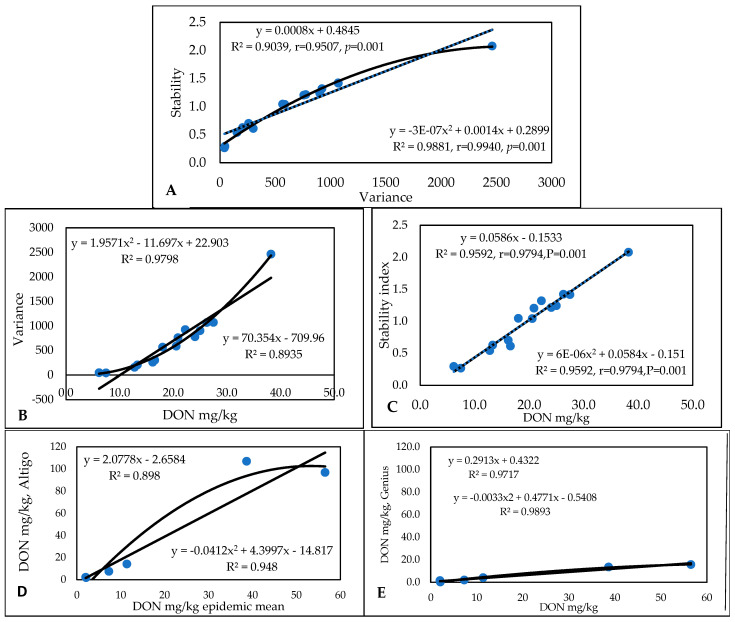
Comparison between variance and stability for the 10 varieties, and DON contamination for variance and stability indices. (**A**) Comparison of the two stability traits. (**B**) Regression for DON mean and stability. (**C**) Regression between DON means and variance. The most unstable and highly susceptible variety Altigo (**D**) and the highly resistant variety Genius (**E**) show highly significant adaptation differences.

For DON (Table S26), sixfold and significant genotype differences were found. Altigo had the highest mean DON contamination (38.2 mg/kg) with values between 1.8 and 107 mg/kg. In the lowest contaminated group, Genius was the best, with 6.2 ppm DON; three Hungarian varieties were close, with 50% less DON contamination than that of GK Csillag, which was successful with good toxin management by fungicides. The toxin values were very high in 2020. The data from 2019 were useful, but in 2021 resulted in poorer differentiation. In spite of the large epidemic variations, the relationships between DON and stability and DON and variance were very close, and the two function types gave very similar results. The stability index showed the same differences as those identified for the other traits in this test and others (Figure 10). The differences between the linear and polynomial functions were moderate, but the variance provided closer correlations. The highest instability (2.07) and highest stability (0.29) are an indicator of large stability differences between the cultivars.

The summary presented in Table 5 shows the three FHB traits, their means, and the two stability traits. GK Pilis and GK Genius have indices that, for all traits, are lower than the mean and are the most stable. Altigo, the susceptible control, produced all of the above-average data. GK Bakony produced similar data to Genius, but here, the head symptoms were above average, indicating additional resistance to DON. GK Börzsöny had rather high DI and FDK values at high instability but, surprisingly, lower DON contamination. GK Csanád was found to have very good results in terms of disease symptoms, but it has a tendency to overproduce toxins and is therefore a high food safety risk. It is remarkable that the correlations between traits and the two stability indices were very close in eight cases, but divergent in six genotypes. For FDK, four cases showed divergence in stability and nine were similar. For DON, seven were divergent and eight showed good agreement. We also found that the DI and FDK data exhibited no significant correlations with DON, and the DI/FDK correlation was also not significant. Variety screening is very useful as, in the best cases, varieties may achieve resistance levels comparable with the genotypes used in FHB background breeding programs. In the correlation analysis, the DI data exhibited no significant correlations with FDK and DON. The FDK /DON correlation is the only exception and supports findings mentioned earlier in this paper.

**Figure 11:**
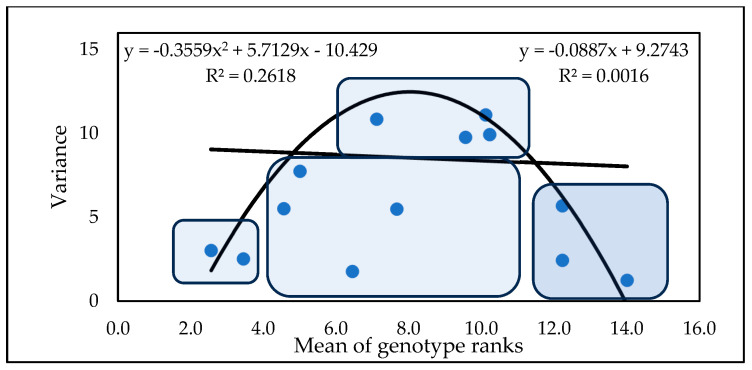
Responses of wheat varieties from Szeged: FHB traits for the variance of the rank means of the 2019–2021 test series.

Figure 11 presents similar results to the previous experiments, showing the variances and the genotype mean ranks of the traits. We see the same distribution of the genotypes as before: two stable varieties with low rankings. Two varieties with low variance have 0–4 rankings (under the regression line), four medium-positioned cultivars have good stability, four genotypes have medium ranks and high instability, and three genotypes have high susceptibility and high stability. As the figures for the epidemic situations are very similar, we have not shown them all in detail. A reasonable regression is shown only by the polynomial function.

**Figure 12:**
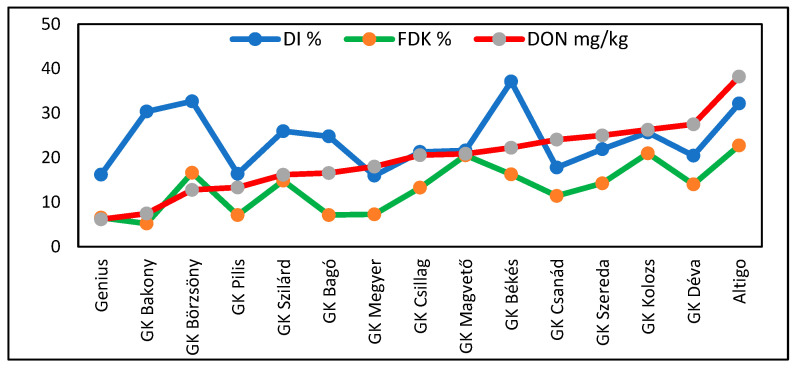
DI, FDK, and DON rankings and their stability in the summary responses of the genotypes (2019–2021).

It is important to visualize the DI, FDK, and DON values (Figure 12) of the variety set presented in Table 5. The data are ranked using the DON data as this is the most important trait governing the trade and utilization of wheat grain. The differences between the genotypes were highly significant for all traits. However, the correlations between DI and the other traits were not. The only significant correlation was the FDK/DON relationship. This is important for forecasting DON contamination and highlights the risk of relying solely on visual head symptoms when, as in this case, breeding for low DON contamination is the main task.

### 2.5. PC Analyses for Experiments 1–4

#### 2.5.1. Experiment 1

The data groups are designated by ellipses or rings. For the disease index, Table S1 served as the database. Figure S15 shows the PC results for the 1990–1993 experiment. For the disease index (Figure S15A), two factors explained 86.55% of the variability, and the genotypes showed continuous variation; three groups could be differentiated. The majority of epidemics (years/isolates, inocula) were close to each other (Figure S15B), but 13 of the 24 gave different responses. For FDK (Table S3), two main factors explain 80.61% of the variability. All genotypes can be classified into one group (Figure S15C). The epidemic situations (Figure S15D) are very similar to those presented in Figure S15B, and 13 epidemic situations significantly differed from the 11 main epidemic situations, producing one compact group. Among these, we observe the same isolates in different positions in different years. Therefore, no genetic relationships between the actual performance for isolates can be determined. For DON (see the data in Table S5), 68% of the variation is explained by factors 1 and 2, which are significantly lower percentages than those for DI or FDK (Figure S15E). The varieties produce two distinct groups (Figure 1D), and genotypes 1 and 10 have very different third groups. The epidemics (Figure S15F) are more compact; with the exception of a major and a smaller group, three epidemics behaved independently. The three traits are close to each other, but equally absent from each other. In terms of the variance, 80% was explained by one factor, while the second factor had an influence of only 13%. The three traits produced one loose group (Figure S15G). The epidemics that occurred in all quarters (Figure S15H) were mostly close to the zero point. Several smaller groups and individual genotypes vary in the field. It seems that most of the genotypes have differing identities.

#### 2.5.2. Experiment 2

The data presented in Table S8 were used for principal component analysis. Variances of 68 and 12% were determined by the two factors. The genotypes were distributed in one group for the disease index at a continuous variation (Figure S16A). A contrary case was found for the DI epidemics; here, a larger and a smaller group were identified, but five genotypes were distributed overall in the graph (Figure S16B). For FDK (original data: Table S11), values of 48% and 16% were determined by the two factors, and the genotypes formed two distinct groups (Figure S16C) with two genotypes positioned differently. For the epidemics, again, three larger distinct groups could be identified, and five had different positions (Figure S16D). The DON (original data presented in Table S13) showed variances of 55 and 14% for the two factors, and the response of the genotypes was used to divide them into two groups. The upper group was less compact (Figure S16E). The epidemics formed three major groups, with four further genotypes not belonging to these groups (Figure S16F). The three traits (Table 3) (90 and 7% from the variance) are rather close together; DON and DI are closer, and FDK is positioned a small distance from them (Figure S16G). The genotypes show wide variation. There are only five genotypes in a group, and the others seem to be independent (Figure S16H).

#### 2.5.3. Experient 3

The original data can be found in Table S17. The DI data (72% and 11% for factors 1 and 2) show genotypes in two groups (Figure S17A). For the epidemics (Figure S17B), two major groups can be identified and 12 genotypes seem to be independent of these groups. For FDK (from Table S19), 74% and 10% explained existing variances (Figure S17C); the density is higher at the x axis, and 2 - 2 genotypes are on the y axis above and below 0.5, which may relate to the main groups (Figure S17C). Most of the epidemics (Figure S17D) are concentrated into one group, and nine have different positions. The DON data (Table S21) exhibit variances of 86% and 4% for factors 1 and 2. The genotypes provide a compact group with similar behaviors (Figure S17E). This compactness can also be seen in the epidemic distribution: there is a large group near the zero point, and eight epidemics are positioned differently (Figure S17F). In the summary figure (Figure S17 G), 74% and 21% of the variance is explained by factors 1 and 2. DON and FDK overlap with each other, and DI is positioned seemingly independently. It is important to note that the genotypes behave as independent units; groups with two or three members are only observed in some cases (Figure S17H). Therefore, there seem to be no common rules for genotype behavior as they appear to have different footprints.

#### 2.5.4. Experiment 4

This experiment (Figure 4) shows very similar PCs to those we observed in the three prior experiments. For DI (Figure S18A, basic data in Table S24), factors 1 and 2 explain 64 and 15% of the variance. The 15 genotypes are distributed loosely, but they are similar to a certain extent as previously observed. The lower number of genotypes can make a difference as the size of the factors is similar, as we found elsewhere. Only genotype 14 seems to stand alone. Again, the epidemics exhibit a wide distribution (basic data in Table S24) without forming any narrow groups (Figure S18B). For FDK (original data in Table S25), 68% and 15% of the variance is explained by factors 1 and 2. Two distinct groups of genotypes were identified (Figure S18C). Here, six epidemics seem to produce a loose group; the other nine form a rather compact group (Figure S18D). The DON responses of the 15 genotypes (Table S25) seem to produce a relatively stable group, with only 1 reacting differently (Figure S18E). Here, 93% and 5% of the variance is determined by factors 1 and 2—the highest rate among the four experiments. The epidemic situations (*n* = 8) show a familiar picture. Three groups have two overlapping members and two stay apart independently. The three traits are rather far apart, and 65% and 28% of the variance is determined by factors 1 and 2. This agrees well with other data from this study (Figure S18G). The genotypes in the summary table show the same picture: every genotype has an independent character, and no definite common features can be detected (Figure S18H).

## 3. Discussion

### 3.1. Stability

The stability of the behavior of a genotype regarding economically important traits is a prerequisite of its successful production. This has long been known for yield, but resistance to biotic and abiotic effects has been much less researched. In this study, we proved that very large stability/instability differences exist between genotypes against FHB, and therefore the selection of varieties with highly stable resistance to FHB is possible. As FHB is not caused by specialized pathogens, the durability of the resistance is not jeopardized and durable resistance is possible [1,23,30]. In heavily epidemic areas, the selection of more aggressive isolates is possible. By segregating populations, the loss of one or more QTLs can weaken resistance. In young varieties, large differences in resistance to FHB can be observed (e.g., Zombor), and if a susceptible line is chosen for propagation, the resistance is lost due to inadequate maintenance. It has been widely reported that Sumai 3 has excellent resistance in all continents where it has been tested, which also supports the idea of stability and durability [55]. The resistance level is connected with higher stability, and higher susceptibility is associated with increasing instability; this is clear from all tests. Another aspect is that stability per se can occur in highly susceptible genotypes, and the rankings show this clearly.

The high variation found in genotypes under different epidemic conditions clearly proves that determining stability or instability requires a much wider testing regime than the present experimental praxis shows. In most countries, there is no reliable information about the resistance level of the cultivars. Therefore, the risk to production is unknown. Resistance tests must be developed in order to determine these risks, and from this, we can identify the stability of the more resistant varieties and breeding lines. By knowing the stability and resistance levels of many Hungarian varieties, we can identify the most appropriate varieties that can withstand FHB with fungicide treatment and other practices. Thus, safe production is possible using currently registered and commercially available varieties.

The formula proposed by Eberhart and Russel [26] indirectly assumes that all regressions are linear; therefore, an increase in the line shows the stability of the traits with precision. The correlations between variance and SI were found to be highly significant in all tests for both the linear and polynomial models; in most cases, the difference was small. Comparing the summary tables of the four experiments shows that the variance in the original trait data had closer correlations with the experimental means than with the stability index, and the differences were often large. Additionally, in many cases, the polynomial function correlated better than the linear model. The conclusion is that both stability counting methods are suitable, but that variance shows better results than the stability index in most cases because when using variance we work with the original data and not from the SI derived from the linear model. Its calculation is also very simple, which is an advantage.

Miedaner et al. [56,57] reported that changes in weather conditions can have an impact on the infection results and that this is a threat to the stability of resistance data. Yan et al. [36] acknowledged the importance of stability across environments through planes and multilocation tests, but only mentioned plans for such studies, and DON tests were not initiated. Sakr [58] found that more resistant and highly resistant varieties were more stable than susceptible varieties, but no special stability analysis was conducted. We have previously found [25] that a small dataset is not sufficient to characterize the stability of a response; therefore, this issue must be addressed. Wheat’s responses to visible symptoms and DON often provide contradictory data; such data seem to be genotype-dependent, and environmental effects can also be responsible for these outcomes. More data have emerged on this effect in recent decades [13,14,24], and more studies are being published with similar DON/symptom disagreements [36]. The conclusion is that higher resistance is often found to be more stable, but in most cases, no experimental material supports these observations as there are simply not enough data to prove this stability [25]. Stability analysis is not possible without a reliable database, and therefore one should be created first. The four experiments conducted in this study provide data that can be used to develop a database suitable for use in variety registration and the breeding of resistant and stable cultivars.

Stability has two meanings. The first is connected to the resistance level. This means that a susceptible genotype exhibits very different DON contamination values during different epidemics: the most resistant ones provide a value close to 0 (SI = 0.03), such as the breeding line from the Szeged FHB program (RSt/NBbZu/Ré/NB/5…, Zu//Ré/NB, Sgv-NN//MM/Sum3), but the most susceptible are higher at SI = 2, or, in exceptional cases, SI = 3. Stability values of 0.3–0.4 are suitable for commercial production under epidemic conditions in Hungary, except under extreme severe epidemic conditions. However, the question arises as to whether this resistance level would be enough in the Yangtze valley. We should mention that the resistance of winter wheat is far less investigated than that of spring wheat. The reason is that in many winter wheat areas the epidemics are less frequent. The second meaning of stability is that susceptibility ranking can also be stable under different epidemic conditions when its position among the most susceptible varieties differs little from the highest rank in a 40-variety set. All four tests found that there are four variety classes based on ranks: (1) Genotypes with a very low ranking and very high stability (low variance and low rank). (2) Genotypes with medium ranking and low-to-medium variance. (3) Genotypes with medium ranking and very high variance. This is the most dangerous group. (4) Genotypes with high and very high ranking and low-to-medium variance. Classes 3 and 4 are not suitable for commercial production.

### 3.2. Isolates, Epidemics, and Environment

The test series proved that the parallel use of more individual isolates significantly increases the usefulness of the data. It also became clear that the correlations between the epidemic situations across genotypes exhibit a rate of non-significant correlations of 50% or higher; that is, the isolates may behave differently not only across experiments, but also in different years, and epidemic severity strongly differs. All four experiments support this conclusion. Fuentes et al. [25] claimed that a small amount of data is not suitable for stability analysis. This experiment series proved that a larger database with at least 24 epidemic cases can give more reliable results regarding stability and the level of resistance.

It is well known that the aggressiveness of *Fusarium* isolates changes over time and varies widely across the population [53,54]. From 50 years of observations, we have found that inocula from the same test tube can also vary in aggressiveness bred in different Erlenmeyer flasks. Not only can the environment, multi-QTL nature of resistance, and year influence the experimental results, but also fungal aspects, which should be kept under control [30]. We know that dilution and mixing influence aggressiveness, but different effects are found for each inoculum [52], and uncontrolled dilution or mixing can lead to unsuccessful test results. For this reason, the assessment of aggressiveness is important to secure the necessary aggressiveness level [29]. We produced almost twice as many inocula and selected only the best. However, even with this approach, an isolate might not work properly and the dataset should be discarded. With more isolates, the danger of unsuccessful results can be reduced significantly. An aggressiveness test just after the inoculation period can advise us as to whether the aggressiveness has changed significantly or not.

### 3.3. Correlations for Traits and Epidemics

The correlation studies revealed two findings. When determining the correlations between genotypes across epidemic situations, the correlations are generally close; this indicates more or less similar rankings across epidemic situations. It was previously posited that one isolate is enough for resistance tests [23,59,60,61]. However, when we count the number of correlations between the different epidemics, this assumption changes: 50% of the correlations are not significant, highlighting the importance of including many epidemics in the test to produce more reliable results. This explains why the one-inoculum model does not work properly. According to present knowledge, specialized races do not exist in the cereal Fusarium population. The finding is that both plant and fungal aspects should be kept under control. In addition, working in one location for 3–4 years is necessary in order to assess different environments beyond the four isolates suggested. In variety registration, two locations are necessary when the decision can be made after two years, but if no solid decision can be made, a third-year test is included.

### 3.4. Relationships Between Traits

Sumai 3 has two major QTLs [12], both originating from the moderately susceptible Taiwanxiaomai from China *(Fhb1*) and the Italian Funo (*Fhb5*). This finding [62] was supported by Mesterhazy [30]: the two QTLs together secure excellent FHB resistance in Sumai 3 as a result of transgressive segregation (the progeny is significantly more resistant than the better parent). Moreover, it has been proven that spray inoculation also detects Type 2 resistance and, therefore, is a good choice for general breeding [27,31]. As regulations often differ, all three traits should be tested. In Sumai 3, the two main QTLs similarly regulate the three main traits, but not all QTL behave this way [31]. The results of this study support the notion of differing regulation in many genotypes—PCA found that the three traits exhibited different fingerprints and no constant groups were identified. Instead, only 2–3 genotypes formed smaller groups. The stability/instability of the genotypes often showed considerable differences between traits, and the correlations between traits followed the pattern observed previously. The correlations between the three traits were closer between DON and FDK than between DI/FDK and DI/DON, but exceptions did occur. The three traits showed similar resistance at a certain rate, but at a larger rate, the differences were striking. The data also support the descriptions of resistance to FDK and DON accumulation provided in 1995 [13]. Beyond this, different genetic regulations can also be causative agents [31,52]. The results clearly support the view that the regulation of resistance and resistance to toxin accumulation can be very different. Beyond this, there are many correlation-breaking genotypes for DI and FDK, where genetic causes may be present alongside other mostly unidentified effects. This is a significant reason why the data on DI, FDK, and DON often disagree; therefore, reliance on visible symptoms alone does not solve the problem. Toxin contamination is the key; therefore, in breeding, a trait that better forecasts mycotoxin contamination is required, which is FDK.

### 3.5. Principal Component Analyses

From the PCAs, we identified different behaviors in relation to different traits. All three traits can have one, two, or, seldomly, three groups for genotype behavior. We did not observe any stable, uniform behavior, and while this may differ between traits, disagreement is more characteristic. In a population where no preliminary information exists or, worse, is not reliable, the only option is to conduct a full screening of every trait to obtain an idea of the risks associated with the genotypes tested. This depends on the composition of the actual genotype set and ecological factors. The epidemic situations for the three traits have similar patterns; one group comprises half of these cases, and the others are located elsewhere in the plotted figures. We also see that the DI, FDK, and DON data are close to each other, but two almost overlap (DON/FDK; DON/DI). Both cases are acceptable, and the correlation and regression analyses support this conclusion. Examining the PC analyses of the mean results from the 3–4-year studies clearly show that the genotypes are more or less uniformly distributed in the four experiments, but at least half of them behave differently. This means that they are not grouped into different units, and all have their own specific character or footprint. This is highly important, because this best describes the value of a given genotype for commercial production. To obtain this knowledge, all traits should be tested. Before testing, we had no idea which behaviors could be identified. These data support the view that the three major resistance types may also have different genetic regulations, and we posit that every genotype has its own special mix of resistance types. We do not have enough proof of this so far, but we expect this to be an important research task in the future. The four tests gave very similar results for the variety rank responses to the PC analyses, indicating that the genotypes generally did not form groups and their distribution was nearly uniform with no determined tendencies. Based on these four tests, we can state that every genotype has its own fingerprint.

### 3.6. The Multi-Toxin Problem

In this study, we analyzed DON, as this is the most important factor. However, recent studies on human and animal blood and urine [19,21,63] teach us that many toxins may remain in these samples, and the DON-free state alone does not automatically guarantee the absence of other toxins in the sample. Recently, its occurrence became clear from naturally infected winter wheat samples, where DON, zearalenone, aflatoxin, and the HT-2 toxin were also identified in amounts higher than the limit value [64]. For two QTLs, common resistance to *Fusarium* spp. was also proven [30,51,52], but this was not conducted for the other QTLs. Studies were also not conducted for the toxins produced by other *Fusarium* spp., such as nivalenol, T-2, and the HT-2 toxin, and their respective resistance. It is important to determine whether the multi-toxin problem can be improved through breeding or through other means, and we must identify the complex methods that may grant a higher level of food safety. The last international and global survey published highly important data on aflatoxin, zearalenone, DON, T-2, fumonisins, and OTA in feedstuffs with very high contamination levels of many toxins [5], with 70% of the positive samples containing three or more toxins. This is an argument for the need to consider a number of other important toxins in addition to DON. There is a better understanding of *F. graminearum* and *F. culmorum*, but the amount of resistance to other *Fusarium* spp. and mycotoxins remains unknown and should be studied further. We suggest that selected lines and varieties should be exposed to a multi-*Fusarium* spp. and toxin test series.

### 3.7. Breeding Aspects

It becomes possible to screen for food safety with a much higher probability, allowing us to simultaneously measure DI, FDK, and DON resistance and the stability of individual traits. As the combinations of these are different depending on the variety, it is clear that, for food safety, it is not possible to conduct these screenings in the absence of toxin data. Of course, there are exceptions, but a breeding program for food safety needs all three traits.

Approximately 10–20% of the genotypes studied exhibited stable responses against individual traits at low infection and toxin levels. In terms of yield, about 90% of the breeding lines are usually discarded. The selection rate is here lower. As the stability of the traits cannot be determined from several types of data, the QTLs identified contain an unknown number of false positives and false negatives. The reliability of the three traits (DI, FDK, and DON) can cause problems in evaluating the genotypes’ responses [31]. As only a small part of this study analyzed DON responses, the contradiction is that we aim to decrease DON mostly by disregarding DON tests [27,65]. Several studies have referred to the different genetic and epidemiologic regulation modes of DON production [66,67,68]. As most QTLs are not validated and their function is not clear, with the exception of a few QTLs (*Fhb1*, *Fhb5*, etc.), the prospect of controlling DON contamination remains poor. The presence of multiple toxins in blood and urine samples is a key factor [21,63,69,70,71,72]. Arce-Lopez et al. [73], in their review, presented a large dataset drawing on global sources surrounding this problem. For us, the key question is as follows: To what extent is the multi-toxin problem a breeding problem? We have data on common resistance to different *Fusarium* spp., but no toxin results mirror such a possibility. A significant amount of research is needed in this area. The most resistant lines prove that high resistance can secure high stability for a long time; the correct measurement of the three traits helps us to identify the best varieties for commercial production and to forecast their adaptation to different conditions. Testing for multiple Fusarium and toxin contaminations may advise us in further research.

Gaire et al. [34] identified good resistance to Fusarium head blight in plants where no known FHB QTL was present. This is also the case in Hungary; for example, the GK Pilis and GK Bakony varieties tested in this study exhibited good resistance but no known QTLs (Toth et al., personal communication). For this reason, spray inoculation is needed as this reflects the total resistance.

By introducing spray inoculation as the main inoculation method, together with the addition of more isolates and more years in experimental praxis, the epidemiological behavior of the genotype and its adaptation can be tested with greater reliability. The consequent identification of FDK and DON has made it possible to analyze the resistance components of a given variety or line which was not possible previously. Gaire et al. [34] contributed significantly to spreading this idea. For this reason, the FHB control of variety registration has become possible and susceptible genotypes can be discarded from production, thus retaining the best variety candidates for commercial production. Breeding methods can be updated, with several highly resistant winter wheat lines showing good potential.

A high level of scrutiny is needed to identify suitable crossing partners; only those that are highly stable for all three traits should be selected for breeding. The FHB control of the F_1_ generation is essential in all breeding programs. Based on our present knowledge, 80–90% of F_1_s can be discarded based on the F_1_ results obtained when FHB resistance was not considered an important trait. When only medium- and higher-level-resistance parents are crossed, the likelihood of producing more-resistant F_1_ plants is significantly higher. In this case, the FHB control of the first yield test on a 5–10 m^2^ plot could be the first step for resistance tests. The careful control of behavior is necessary if we are to obtain the most suitable lines for testing and submission to national plant registration offices. As the suggested methodology shows, this is possible. As all genotypes have their own fingerprints, as found in the PCAs conducted in all experiments, the three traits are needed for the testing process (DI, FDK, and DON). In earlier stages, pooling the samples for DON data can lower the costs. It is important to consider natural multi-toxin data in variety testing using non-inoculated controls; one must also check the trucks at harvest for toxin contamination at harvest to see the important preharvest toxins. Such data can provide feedback for breeders and producers, contribute reliable information on toxin contamination in a given country, and prevent the mixing of healthy and toxin-contaminated lots.

The ability to select medium or good resistance from unknown varieties with an SI of 0.5 or lower is important. This allows resistance to be combined with optimized fungicide technology to yield heathy grain at a much higher probability than in regular cases. As such varieties are in commercial fields, identification is possible. This new methodology is also useful for plant protection and plant production tests, where the influence of agronomic or plant protection practices are tested to improve food safety performance [74]. However, it is also possible to breed significantly more-resistant winter wheat varieties that may not need chemical treatment, as we demonstrated with several genotypes tested. It is also possible to combine resistance with other physical decontamination and cleaning methods to save as much grain as possible as we do not have immunity.

## 4. Material and Methods

All methodical aspects of the three first experimental series can be found in Mesterhazy et al. [14,24]; therefore, in this paper, only their basic tables are used and presented in the Supplementary Materials. Otherwise, we do not address the results and conclusions of their scientific testing. Descriptions and methodological details are provided for the most recent test only. In all tests, we used 4–8 isolates in a year; the tests lasted 3–4 years. In all cases, full FDK and DON analyses were conducted without exception. We considered the disease index as a multiplication of incidence and severity, but it can also be estimated directly as a percentage of the diseased spikelets in a group of heads; we applied this method. In susceptible genotypes, bleaching is a well-known event, where dead spikelets above the infection point are killed by the lack of metabolism. Grain with these genotypes is brown, healthy-looking, and shriveled. Bleaching can lead to overestimation of the level of visible infection. Following Bai [75], only one reading of DI is now made, 21 days after inoculation. This can be suitable for serial breeding work. We took 4–5 readings, using their means or AUDPC counts to better follow the epidemic development. FDK was found to be useful in our breeding praxis—50% of the genotypes selected for good resistance based on visual cues had to be discarded because of their high FDK values. After the last reading, disease development was slow or halted, but sped up when conditions were rainy. At threshing, all diseased and white-, rose-, and brown-colored grains that were shriveled were kept. A high rate of shriveled, brown grains is a reflection of the level of bleaching. This type of estimation can be performed with a yield of 10–15 heads. In addition, the classic white or rose kernels and dull and flat kernels can also be estimated; these are mostly indicators of late and weak FHB infection. When we added this rate to the regular FDK grains, the correlation with DON became closer by about 0.1, indicating that the symptoms were initial FHB infection markers.

### 4.1. Plant Material and Experimental Design

The plant material consisted of Hungarian and foreign breeding lines and genotypes; their names are presented in the tables. A more detailed description is given in Mesterhazy et al. [14,24,51]. In the fourth test, only genotypes from Cereal Research Ltd. were tested. All experiments were conducted in three replicates of a randomized block design combined with factorial analysis because the four different inocula (isolates or mixtures) within plots.

### 4.2. Inoculations and Their Evaluation

#### 4.2.1. Isolates

Eight isolates from 1990 to 1993 were used: 12216 FgH, 12377 FgH, 12375 FcH, 12551 FcH, 223 FcD, 39.01 NL, 89.4 FcF, and 207 FgD (Fg: *F. graminearum*, Fc: *F. culmorum*, H: Hungary, D: Germany, NL: The Netherlands). The same isolates from 1994 to 1996 were used, except for 12216 and 39-01, which were changed to Fg40 and Fg44 from Austria. In the 2019–2023 test period, we used four types of *Fusaria* inocula for artificial inoculation [14]. For the 2009–2012 test, the isolates used were Fg 12377, Fg 46.06, Fc 89.4, Fc 12375, Fg 13.05, and Fc 12551. The 2021–2022 test was established with a single *F*. *graminearum* isolate (No. 19.42). The second isolate was a *F*. *culmorum* strain (No. 12.37). The third inoculum was a mixture of three different *F*. *culmorum* isolates (Nos. 12.37, 12.51, and 52.10). Similarly, the fourth inoculum consisted of a mixture of three different *F*. *graminearum* isolates (Nos. 19.42, 46.06, and GB 78). All isolates were identified using PCR markers in addition to traditional methods [28,29]. The approach to the identification of the isolates followed the mycological methods presented by Booth [54] and later by Leslie and Summerell [76]; however, after the discovery and evaluation of the PCR markers [77,78], molecular identification was also routinely applied. Details are given in György et al. [79] and Tóth et al. [77,78]. Inocula were stored before and after inoculation at 4 °C. At the end of the inoculation, a control for aggressiveness was again developed.

#### 4.2.2. Inoculum Production

**Figure 13:**
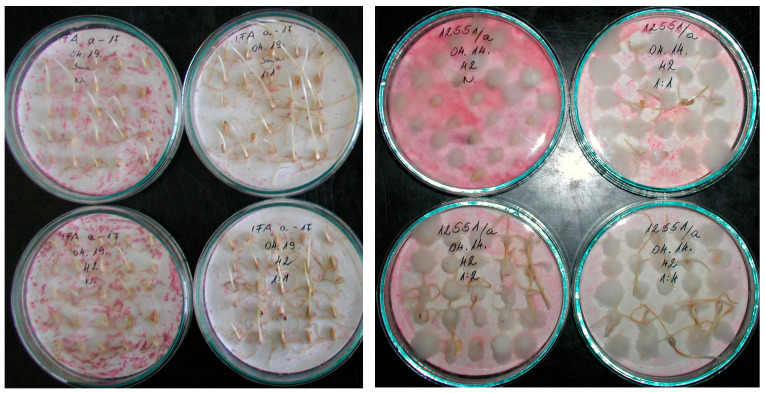
Low-aggression (**left**) and highly aggressive (**right**) inocula with no dilution (N), and 1:1, 1:2, and 1:4 dilutions of the original inocula.

Inoculation was performed using the bubble breeding method [52]. The inoculum was produced in 10 L heat-stable glass balloons filled with 9.5 L ion-changed Czapek–Dox liquid medium and then sterilized in an autoclave (2 h at 1.2 atm pressure). After inoculation, sterile air was passed through a glass tube on the bottom of the flask to keep the medium moving at room temperature. After a week, the inoculum was ready for use. The aggressiveness was tested using the Petri dish method [8,44]. Every inoculum was checked at original concentrations and dilutions with ion-changed water at 1:1, 1:2, and 1:4 for two varieties. The Petri dishes (12 cm diameter) were inserted with double-layer filter paper and 9 mL of the suspension was uniformly distributed on their surface. Figure 13 shows two inocula with the original concentration and three dilution grades. For the inoculations, inocula were chosen that had stable aggressiveness at the four dilution rates or for which only a small decrease was recorded. The unstable and nonaggressive inocula were discarded; thus, 2–3-fold more inocula were produced from which the best ones were chosen.

#### 4.2.3. Inoculation

**Figure 14:**
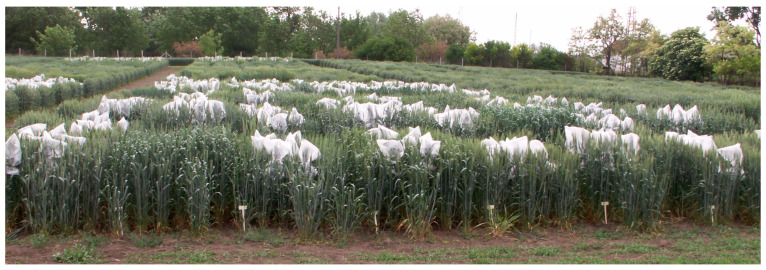
Covering the inoculated bunches in six-row plots using four isolates, with two bunches per isolate. Tests involved two plot replicates, so we had eight datasets for each genotype in a given year/location.

Mixing normally reduces aggressiveness compared to the most aggressive inoculum component, but this is normally higher than the arithmetic means of those of the participating inocula [52]; therefore, mixing was applied only in certain cases. To avoid this, about a double amount of inocula was produced. All inocula were tested separately, and after the last inoculation another test was conducted to check any possible changes. The spray inoculation method was used; approximately 15 heads were sprayed with 10–15 mL suspension (depending on the ear size) from all sides using a hand-held sprayer in the middle flowering stage. Then, the groups of heads were covered with polyethylene bags for 48 h to ensure high humidity, encouraging infection (Figure 14). In cooler temperatures, the glasshouse effect ensured higher temperatures inside, helping the infection process.

As the susceptibility window lasts for a week [79], we were able to group the inoculations for approximately 5 days without risking any influence on disease development. Then, the groups of heads were covered with polyethylene bags for 48 h to ensure high humidity, encouraging infection (Figure 14). In cooler temperatures, the glasshouse effect ensured higher temperatures inside, helping the infection process. This is an advantage over mist irrigation, which cools the heads and inhibits disease development.

#### 4.2.4. Disease Evaluation

The disease index was rated as a percentage of diseased spikelets; this was performed 10, 14, 18, 22, and—in later genotypes—26 days after inoculation [24,46]. For DI, we present the means of the 4–5 readings. The later genotypes needed one reading more, so the last reading is in the same developmental stage in both cases. This is a problem that the literature seems to ignore [75]. After harvest, careful threshing was conducted without losing the infected and shriveled grains. This was performed using an LP 180 laboratory thresher (Wintersteiger Gmbh, Ried, Austria) and a fine cleaning tool made by Ets Plaut-Aubry, an air separator (41290 Conan-Oucques, Conan-Oucques, France). FDK was rated according to the percentage of white- and rose-colored discoloration of the grains [24,35].

In the test for DON, we lost data from 1991 as a result of a computer problem; therefore, here, 24 epidemic cases were considered for evaluation (1990, 1992, and 1993). For the toxin analyses, seed samples from all bunches were analyzed using earlier an HP 1090 M, and later from 2011, an Infinity 1260 HPLC from Agilent (Agilent Technologies Santa Clara, CA, USA). DON analyses were conducted according to Mesterhazy et al. [24]: from each bunch, 6 g of grain was taken (less when the sample was smaller due to highly infected grains and a lower grain number). We received the toxin data from the lab; here, n.d. (not detected) was used to indicate that no detectable amount was found. For all of these data, “0” was used in the statistical evaluations to indicate that toxin contamination below the detection limit was possible (Berenyi et al., 2023) [64]. We used a color system that can be applied for the visualization of genotype ranking in maize [80].

#### 4.2.5. Evaluation of Stability

The stability was calculated using two procedures. The first was described by Eberhart and Russel [26]. The isolates, methodical variants, and years were combined into one table, representing the epidemic variants. First, the means for each epidemic situation were produced; these data were considered for the x axis. Then, a linear regression analysis was conducted between the means of the 36 epidemics (x axis), and the datasets of all of the individual varieties were processed separately. In the function of y = a + bx, “b” is the increase in the line representing the stability index. As we counted hundreds of values for this stability index, we found that not all functions were purely linear; other function types, such polynomial values, fitted better, e.g., the correlations with the other functions were closer. The second procedure was the variance of the epidemic or genotype data calculated using the built-in function in the Excel one-way ANOVA. As this is easy to calculate, we considered whether this variance would be more suitable for estimating stability than SI [26]. Theoretically, it is possible that this would be of equal value or better than the b values of the linear function [26], as it is neutral for linear or other function types of data. For this reason, for all traits, both stability parameters (SI and variance) were calculated and compared.

### 4.3. Statistics

The transformation of data regularly occurs in the scientific literature. The reason for this is that the data must have a normal distribution, and this can be achieved through log transformation or another kind of transformation (but not always) [25]. We did not use transformation because we wanted to know the LSD values of the original data and not the statistics of the transformed data. For all traits (DI, FDK, and DON), the original data are important. The findings showed that with a larger database, the statistical differences are very small, mostly highly significant, and reflect the original data. In our original studies, we concentrated on the yearly means, and the yearly data were not treated separately. In the first experiment, for example, we obtained data from eight isolates over three years, with a total of 24 epidemics. In this way, often-significant interactions could be recorded. For the correlations, regression analyses were conducted using Excel’s built-in programs (Microsoft Office 365 Excel 2021 Professional Plus, Microsoft Inc., Redmond, WA, USA). For the three-, four-, or five-way ANOVAs, the functions of Sváb [81] and Weber [82] were applied, as these analyses are missing from the statistical programs that are available on the market. For the principal component analyses, the free version of the Statistica 13.0 program [83] was used (Informer Technologies Inc., www.informer.com, Tibco, Santa Clara, CA, USA, accessed on 12 February 2022). In the correlation studies, we examined the correlations between the genotypes across the epidemic situations; in these cases, we tended to observe closer correlations. When examining the relationships between the responses among epidemics (isolates*years) across genotypes, we determined similarities or dissimilarities among the responses of varieties to different isolates. Thus, these approaches are important, because they highlight the probability of an agreement between resistance responses to different isolates and may help us to understand the complexity of FHB resistance expression and toxin accumulation.

## 5. Conclusions

The epidemiological aspect, e.g., the stability of FHB resistance and its traits, should be considered in resistance evaluations to identify the adaptation of a given genotype to different ecological conditions. Of the two stability procedures, variance yielded more reliable results; therefore, this can be produced from the simple application of a one-way ANOVA. This approach could also be used for the evaluation of yielding ability in multilocation and multiyear tests. The PC analyses showed that all genotypes have their own fingerprints based on their mean performance, indicating the need to use the DI, FDK, and DON susceptibility data to better describe the adaptation behaviors of the genotypes. We identified the most appropriate stability level in commercial cultivars necessary for successful commercial production. Very high resistance was achieved in the breeding lines, and their high stability was also proven in winter wheat varieties for all three traits. This proves the possibility of consequent breeding. Significant progress was made in terms of updating FHB resistance breeding and testing. These results indicate that FHB resistance could be included as a breeding trait in varieties for which the ability to adapt to different epidemic conditions is crucial, and it will also be important in mitigating other diseases or abiotic stresses [84,85]. Approximately 80% of commercial varieties are susceptible or highly susceptible to the aforementioned infections; therefore, their withdrawal from use and the banning of susceptible varieties will significantly improve food and feed safety within 2–3 years. This rate seems high, but in regular breeding, approximately 90% of the material between breeding steps is regularly discarded. The rates in different programs may significantly differ.

## Figures and Tables

**Table 1:** Ranks of the genotypes in the FHB resistance tests from 1990 to 1993. Disease index for 31 epidemic situations and 20 genotypes [14].

Line No.	Isolate/Genotype	Sgv/GT…	85.92	Bence	Zombor	Szõke	Ok-Sp	Bty-Mo*Kr	Mon-Ar	Ni-Kr*Dol	Kri-Mon	Arina	SVP 75059-28	SVP72059-32	SVP 72017	SVP 72005	Copain	Rechsler	RC103	82F328	81F349	Mean	Variance
1	12216	**1**	**3**	**2**	**6**	**6**	**5**	**4**	**1**	**1**	**1**	**1**	**3**	**1**	**6**	**2**	**1**	**4**	**1**	**2**	**4**	2.8	3.5
2	39.01	**6**	**1**	**1**	**5**	**3**	**7**	**1**	**13**	**3**	**2**	**6**	**7**	**4**	**8**	**10**	**3**	**7**	**2**	**3**	**3**	4.8	10.2
3	223	**12**	**2**	**8**	**8**	**4**	**2**	**3**	**15**	**4**	**4**	**4**	**1**	**6**	**1**	**3**	**13**	**12**	**4**	**7**	**1**	5.7	18.1
4	89.4	**16**	**5**	**7**	**9**	**5**	**3**	**2**	**11**	**5**	**6**	**5**	**8**	**8**	**5**	**7**	**8**	**3**	**3**	**5**	**2**	6.2	10.8
5	207	**15**	**4**	**3**	**2**	**2**	**1**	**5**	**18**	**2**	**3**	**3**	**2**	**2**	**4**	**6**	**7**	**11**	**5**	**1**	**11**	5.4	21.7
6	39.01	**9**	**7**	**4**	**7**	**12**	**6**	**7**	**5**	**6**	**10**	**7**	**5**	**3**	**7**	**1**	**4**	**1**	**8**	**12**	**14**	6.8	11.4
7	12377	**2**	**6**	**6**	**4**	**1**	**8**	**10**	**3**	**14**	**8**	**14**	**6**	**18**	**9**	**4**	**11**	**9**	**6**	**9**	**5**	7.7	17.6
8	12216	**5**	**12**	**17**	**12**	**9**	**4**	**14**	**9**	**11**	**9**	**9**	**15**	**7**	**3**	**18**	**5**	**13**	**11**	**11**	**7**	10.1	16.7
9	207	**14**	**8**	**5**	**3**	**15**	**11**	**9**	**8**	**10**	**12**	**15**	**10**	**9**	**11**	**5**	**6**	**5**	**7**	**10**	**6**	9.0	11.2
10	12551	**3**	**13**	**21**	**17**	**16**	**13**	**6**	**6**	**13**	**5**	**2**	**9**	**5**	**2**	**12**	**18**	**14**	**10**	**4**	**8**	9.9	30.8
11	12551	**7**	**16**	**12**	**11**	**21**	**9**	**13**	**4**	**8**	**11**	**22**	**14**	**10**	**10**	**8**	**14**	**2**	**9**	**18**	**12**	11.6	24.4
12	12375	**13**	**19**	**13**	**1**	**11**	**14**	**17**	**12**	**9**	**13**	**13**	**4**	**15**	**12**	**11**	**2**	**16**	**14**	**16**	**13**	11.9	21.2
13	12216	**11**	**9**	**10**	**16**	**8**	**10**	**8**	**16**	**15**	**22**	**10**	**16**	**11**	**16**	**16**	**12**	**23**	**15**	**20**	**15**	14.0	18.6
14	12377	**4**	**11**	**9**	**15**	**10**	**15**	**15**	**14**	**16**	**16**	**12**	**17**	**17**	**13**	**14**	**9**	**20**	**17**	**19**	**16**	14.0	14.1
15	89.4	**17**	**20**	**11**	**18**	**18**	**19**	**12**	**7**	**7**	**17**	**20**	**11**	**16**	**15**	**9**	**10**	**6**	**12**	**14**	**9**	13.4	20.1
16	223	**19**	**14**	**15**	**13**	**13**	**12**	**11**	**19**	**21**	**18**	**17**	**13**	**13**	**14**	**13**	**15**	**17**	**20**	**17**	**10**	15.2	9.3
17	12216	**8**	**18**	**19**	**10**	**19**	**24**	**22**	**2**	**17**	**15**	**29**	**21**	**12**	**18**	**17**	**20**	**8**	**13**	**13**	**19**	16.2	37.1
18	12377	**10**	**21**	**24**	**24**	**22**	**18**	**23**	**10**	**20**	**7**	**8**	**24**	**19**	**22**	**15**	**23**	**10**	**16**	**6**	**26**	17.4	41.6
19	12551	**28**	**15**	**14**	**14**	**7**	**17**	**16**	**26**	**18**	**27**	**16**	**18**	**14**	**17**	**20**	**16**	**28**	**21**	**21**	**18**	18.6	27.7
20	89.4	**20**	**17**	**16**	**19**	**17**	**16**	**18**	**23**	**22**	**28**	**19**	**12**	**21**	**21**	**24**	**17**	**27**	**22**	**22**	**25**	20.3	15.2
21	223	**21**	**24**	**20**	**22**	**24**	**31**	**25**	**17**	**12**	**21**	**31**	**22**	**23**	**29**	**26**	**24**	**15**	**29**	**15**	**22**	22.7	26.1
22	12375	**18**	**31**	**25**	**25**	**26**	**30**	**24**	**20**	**19**	**14**	**11**	**26**	**31**	**23**	**27**	**31**	**22**	**26**	**8**	**31**	23.4	42.8
23	207	**30**	**22**	**18**	**21**	**20**	**22**	**21**	**29**	**23**	**29**	**23**	**19**	**25**	**19**	**29**	**19**	**29**	**28**	**25**	**27**	23.9	15.9
24	12375	**22**	**26**	**27**	**28**	**28**	**21**	**27**	**21**	**25**	**20**	**18**	**28**	**20**	**20**	**19**	**29**	**18**	**19**	**28**	**20**	23.2	15.4
25	207	**23**	**10**	**28**	**27**	**27**	**20**	**31**	**27**	**29**	**26**	**25**	**27**	**28**	**25**	**21**	**25**	**19**	**18**	**24**	**17**	23.9	24.1
26	12375	**31**	**23**	**22**	**20**	**14**	**27**	**19**	**31**	**24**	**31**	**24**	**23**	**22**	**26**	**30**	**21**	**31**	**31**	**23**	**24**	24.9	22.1
27	12551	**24**	**29**	**26**	**26**	**25**	**26**	**28**	**24**	**27**	**19**	**26**	**25**	**24**	**31**	**25**	**26**	**25**	**27**	**27**	**21**	25.6	6.4
28	39.01	**27**	**25**	**23**	**23**	**23**	**25**	**20**	**30**	**26**	**30**	**21**	**20**	**27**	**27**	**31**	**22**	**30**	**30**	**26**	**30**	25.8	12.7
29	12377	**29**	**27**	**30**	**31**	**29**	**28**	**26**	**22**	**28**	**25**	**30**	**30**	**26**	**30**	**28**	**30**	**26**	**24**	**31**	**23**	27.7	6.9
30	223	**26**	**30**	**29**	**29**	**30**	**29**	**30**	**25**	**30**	**23**	**28**	**29**	**30**	**28**	**23**	**28**	**21**	**25**	**30**	**28**	27.6	7.5
31	89.4	**25**	**28**	**31**	**30**	**31**	**23**	**29**	**28**	**31**	**24**	**27**	**31**	**29**	**24**	**22**	**27**	**24**	**23**	**29**	**29**	27.3	9.6
Mean		16	16	16	16	16	16	16	16	16	16	16	16	16	16	16	16	16	16	16	16	16	18.4
DI Mean		28.1	26.2	23.4	23.0	23.0	20.5	20.1	18.8	18.0	17.9	16.6	16.0	14.8	12.8	12.8	12.0	11.5	10.7	8.5	7.3	16.0	17.1

Dark green: less than 50% of the column mean; light green: 51–100% of the mean; yellow: up to 15% above the mean; orange: all above this limit.

**Table:** 

Genotypes	Disease Index			FDK			DON			Mean
	Mean	Variance	Stability	Mean	Variance	Stability	Mean	Variance	Stability	
**Arina**	8.51	77.35	0.612	5.9	66.2	0.320	0.33	0.497	0.200	17.77
**SVP 75059-28**	10.67	97.91	0.625	10.9	224.8	0.660	1.31	2.509	0.380	38.87
**SVP 72017**	7.26	42.21	0.395	13.0	359.4	0.810	1.19	4.673	0.820	47.75
**81F349**	11.46	219.82	0.679	10.9	252.9	0.660	0.41	0.382	0.120	55.27
82F328	11.97	97.56	0.664	16.0	409.6	0.910	1.43	23.152	1.260	62.50
Kri-Mon	16.03	208.90	1.016	12.8	340.5	0.730	0.68	1.257	0.560	64.72
Szõke	16.65	192.54	0.834	14.8	379.6	0.710	2.27	21.061	1.130	69.96
Copain	14.75	104.22	0.728	18.7	595.1	0.820	1.45	6.564	0.860	82.57
RC103	12.83	111.26	0.722	18.3	628.7	1.050	1.84	10.107	1.070	87.32
85.92	12.78	131.34	0.733	19.0	612.7	1.170	2.79	17.842	1.780	88.91
Bty-Mo*Kr	22.99	429.32	1.380	17.1	476.8	0.990	3.73	40.113	2.120	110.51
Sgv/GT...	17.91	336.44	0.932	21.4	613.3	0.590	2.34	8.143	0.190	111.25
SVP72059-32	20.46	238.02	1.016	25.9	735.7	1.330	2.47	11.604	0.950	115.28
Bence	18.82	329.63	0.947	24.3	701.0	1.170	1.80	8.605	1.050	120.81
SVP 72005	26.20	422.11	1.388	34.8	611.4	0.928	2.28	22.611	1.670	124.81
Ni-Kr*Dol	23.03	614.99	1.750	17.2	478.4	0.970	1.16	4.271	0.510	126.93
Mon-Ar	23.37	629.52	1.715	17.6	478.6	1.010	1.21	4.110	0.300	128.60
**Rechsler**	18.00	337.01	1.329	28.7	1048.5	1.600	2.56	17.980	1.160	161.88
**Ok-Sp**	20.07	440.55	1.425	21.9	1022.4	1.390	1.82	13.268	1.190	169.34
**Zombor**	28.14	325.17	1.126	39.4	1192.1	1.490	7.77	58.999	2.570	184.08
Mean	17.09	269.29	1.001	19.4	561.4	0.965	2.04	13.887	0.994	98.45
LSD 5%	3.09			2.38			0.54			

Dark green: less than 50% of the column mean; light green: 51–100% of the mean; yellow: up to 15% above the mean; orange: all above this limit; **bold**: good resistance and good stability for all traits; **red bold**: high sensitivity in all traits.

**Table:** 

FHB Trait	Trait	Disease Index			FDK			DON	
		Mean	Variance	Stability	Mean	Variance	Stability	Mean	Variance
DI	Variance	0.815 ***							
	Stability	0.837 ***	0.959 ***						
FDK	Mean	0.757 ***	0.393	0.436					
	Variance	0.608 **	0.355	0.420	0.858 ***				
	Stability	0.540	0.363	0.456 *	0.740 ***	0.895 ***			
DON	Mean	0.602 **	0.178	0.209	0.746 ***	0.695 ***	0.582 **		
	Variance	0.572 **	0.164	0.220	0.655 **	0.570 **	0.512 *	0.920 ***	
	Stability	0.487 *	0.074	0.171	0.658 **	0.596 **	0.615 **	0.817 ***	0.908 ***

*** *p* = 0–0.001, ** *p* = 0.01, * *p* = 0.05. Yellow highlight: between trait and stability traits, dark green: between traits, light green: Between stability index for traits, blue: between variances to traits.

**Table:** 

	Disease Index			FDK			DON		
Genotypes	Mean	Variance	Stability	Mean	Variance	Stability	Mean	Variance	Stability
**Sgv-NB/MM-Sum3**	2.61	4.3	0.10	0.92	2.33	0.04	0.32	0.4	0.04
**Arina**	5.28	46.6	0.36	3.88	24.02	0.17	1.28	7.8	0.16
**Sgv-NB/MM-Sum3 .252**	3.45	10.1	0.13	2.03	25.75	0.07	1.70	9.2	0.15
**Ringo Sztár**	10.61	132.6	0.68	7.08	112.48	0.46	2.06	6.0	0.14
**RSt-MM/NB**	7.95	37.8	0.23	5.02	110.42	0.12	2.26	21.2	0.27
DH 84.42	20.58	501.0	1.39	17.26	424.72	0.99	5.59	89.1	0.62
**81.60-NB/Kő**	20.83	234.2	0.76	11.97	201.99	0.77	6.07	45.1	0.31
P4371.88	19.09	241.0	0.92	17.19	425.69	1.00	6.15	86.3	0.63
SK8090	14.82	101.0	0.57	15.54	434.45	1.21	6.55	141.7	0.70
Siouxland	13.10	119.3	0.48	25.00	714.71	1.58	7.28	65.7	0.55
P2118.89	16.92	95.7	0.61	30.42	335.40	0.77	9.54	116.9	0.62
Sum3^2-81.60	15.07	238.3	0.93	11.59	310.00	0.74	9.72	262.0	1.15
Rechsler	23.33	343.0	1.18	28.09	527.06	1.48	11.90	239.0	1.01
Sgv-GT...	28.31	402.0	1.31	15.73	393.76	1.22	12.71	430.3	1.37
Kincső	24.10	314.9	1.10	18.43	536.37	1.29	13.67	155.9	0.52
Kende	27.90	585.1	1.63	22.32	259.66	0.90	14.41	230.4	1.17
Bence	20.91	364.9	1.25	16.06	444.99	1.30	18.34	486.7	1.44
Szőke	26.15	535.8	1.55	18.49	355.44	0.80	18.99	585.2	1.81
78.1.04	31.72	479.3	0.70	40.10	877.13	1.16	19.03	444.4	0.78
Góbé	31.51	335.3	0.98	32.09	801.40	1.47	19.31	230.9	0.52
Jbj-5o	30.78	463.6	1.25	42.34	487.36	1.28	23.69	374.3	1.35
Csaba	38.92	838.5	1.90	26.45	445.01	1.39	30.72	1001.1	2.25
Zugoly	38.37	577.2	1.48	50.43	774.84	1.37	32.23	666.9	1.34
Őrség	34.52	580.0	1.64	43.17	808.92	1.74	35.97	1288.4	2.61
Zombor	35.74	820.6	1.86	35.43	648.78	1.53	42.25	2130.2	3.48
Mean	21.70	336.1	1.00	21.48	419.31	0.99	14.07	364.60	1.00
LSD 5%	0.98			2,70			8.58		

Bold low DI, FDK and DON as well as SI and variance presenting genotypes. Dark green: less than 50% of the column mean; light green: 51–100% of the mean; yellow: up to 150% above the mean; orange: all above the 150% limit.

**Table:** 

Correlations/Traits	Triplets	Disease Index			FDK			DON	
		Mean	Variance	Stability	Mean	Variance	Stability	Mean	Variance
DI	Variance	0.91 ***							
	Stability	0.86 ***	0.944 ***						
FDK	Mean	0.83 ***	0.66 ***	0.59					
	Variance	0.75 ***	0.569 *	0.50	0.86 ***				
	Stability	0.79 ***	0.65 ***	0.686 ***	0.777 ***	0.889 ***			
DON	Mean	0.88 ***	0.86 ***	0.80 ***	0.808 ***	0.698 ***	0.717 ***		
	Variance	0.68 ***	0.78 ***	0.71 ***	0.56 **	0.49 *	0.55 **	0.90 ***	
	Stability	0.73 ***	0.84 ***	0.83 ***	0.576 **	0.49 *	0.61 **	0.89 ***	0.95 ***

*** *p* =0–0.001, ** *p* = 0.01, * *p* = 0.05. Yellow: difference in correlation to stability and variance. Blue: relationships between variances of the three traits. Orange: relationships between stabilities of the three traits. Dark green: correlations between the traits.

**Table:** 

Genotype	DI %			FDK %			DON mg/kg		
	Mean	Variance	Stability	Mean	Variance	Stability	Mean	Variance	Stability
Rst/NBb	8.71 *	76.9	0.75	0.91	4.77	0.09	1.87	13	0.23
Zu//Ré/NB/5/DH …	5.30	51.4	0.85	7.87	312.54	0.91	2.44	24	0.39
Zu//Ré/NBb	8.84	99.5	1.30	7.23	298.03	0.88	3.80	40	0.41
Zu//Ré/NBa	10.00	122.3	0.94	5.71	150.99	0.59	4.07	35	0.32
SzD 5319	4.53	28.0	0.98	7.61	155.39	0.73	4.45	54	0.50
MIDAS	3.78	31.4	1.02	7.41	151.49	0.77	5.26	112	0.70
GALLUS (SzD 8583)	5.31	58.3	0.72	5.30	131.51	0.64	5.58	145	0.84
SzD 4511	4.77	33.0	0.86	11.82	308.51	1.04	5.74	64	0.50
BALATON	10.66	121.2	1.00	14.45	428.05	1.32	6.00	175	0.89
SzD 5066	7.19	68.9	1.40	9.90	269.69	1.06	6.02	207	0.97
SzD 0016 S	3.72	37.2	0.91	8.25	175.82	0.70	6.10	95	0.61
Ttj/RC103/3/Sgv/NB//MM/Sum3	8.78	91.9	0.45	9.11	262.45	1.00	6.26	126	0.76
SzD 0016 F	6.80	59.0	0.54	7.77	192.21	0.89	6.45	141	0.83
SzD 5501	6.38	74.8	0.71	9.70	204.00	0.83	6.51	79	0.55
GK HOLLÓ	9.31	99.9	0.93	9.44	184.08	0.80	6.81	76	0.57
SzD 6401	4.75	58.2	1.11	10.79	376.29	1.19	7.33	201	1.01
GK FÉNY	15.10	235.7	1.45	9.66	366.43	1.16	7.35	194	0.96
GK CSILLAG	15.64	170.7	1.09	11.71	402.86	1.06	7.42	213	0.96
Attila/3/Sgv/NB//MM/Sum2	12.22	165.9	1.21	10.65	317.77	1.04	7.69	178	0.92
GK BÉKÉS	13.80	187.0	1.35	9.90	127.36	0.67	7.97	99	0.67
GK PETUR	8.25	96.7	1.27	11.00	286.36	0.82	8.03	185	0.91
GK HATTYU	8.79	99.4	0.90	14.73	467.86	1.35	8.03	273	1.11
RAINER	8.94	92.4	1.12	13.78	444.51	1.26	8.32	282	1.14
Be/SK 48.21// FHB 142	10.17	113.5	1.06	12.45	427.35	1.21	9.04	216	0.98
SzD 6626	11.06	182.7	1.32	11.61	254.52	1.01	9.37	307	1.19
Rst/Nba	7.16	150.6	0.50	8.51	178.35	0.58	9.48	254	1.08
SzD 5377	7.93	96.8	1.64	14.92	460.58	1.32	10.05	250	1.08
GK HUNYAD	8.09	120.5	1.07	13.48	438.10	1.34	10.87	461	1.27
SzD 18364 (B-18364)	6.88	84.7	0.52	9.92	193.30	0.77	11.06	292	1.17
GK ÉLET	19.82	264.3	1.53	18.03	427.79	0.98	11.11	275	0.93
SzD 5205	11.25	203.5	0.81	11.51	310.45	1.13	11.90	588	1.70
GK SZALA	7.64	108.1	0.53	14.95	439.84	1.33	12.17	379	1.37
SzD 5417	15.89	353.3	0.82	18.29	519.56	1.37	12.27	547	1.63
SzD 6701	9.50	175.1	1.22	11.53	309.41	1.06	12.31	447	1.49
SzD 5275	8.12	80.9	0.50	19.45	557.92	1.01	12.54	228	0.88
GK KALÁSZ	16.86	193.0	1.57	15.37	426.83	1.13	12.96	267	0.99
GK PIACOS	16.39	196.4	0.99	16.53	442.22	0.98	13.19	405	1.26
SzD 6515	10.66	211.1	1.19	22.04	621.11	1.47	13.33	751	1.87
SzD 7257	10.10	154.0	0.98	16.41	480.71	1.34	14.86	937	2.16
GK KAPOS	12.50	270.5	0.89	17.64	434.28	1.17	17.52	962	2.18
Mean	9.54	127.97	1.00	11.68	323.53	1.00	8.59	264.46	1.00
LSD 5%	3.72			1.41			2.14		

Dark green: lower than 50% of mean; light green: 51–100% of mean; yellow: 101–150% of mean; orange: all above 150%.

**Table:** 

Correlations		Disease Index (DI)			FDK			DON		
Trait		Mean	Variance	Stability	Mean	Variance	Stability	Mean	Variance	Stability
DI	Variance	0.861 ***								
	Stability	0.432 **	0.297							
FDK	Mean	0.478 **	0.557 **	0.194						
	Variance	0.414 *	0.458 **	0.226	0.911 ***					
	Stability	0.264	0.368 *	0.258	0.786 ***	0.886 ***				
DON	Mean	0.493 **	0.637 ***	0.074	0.820 ***	0.673 ***	0.562 ***			
	Variance	0.340 *	0.609 ***	0.028	0.693 ***	0.607 ***	0.588 ***	0.848 ***		
	Stability	0.330 *	0.606 ***	0.037	0.701 ***	0.627 ***	0.659 ***	0.854 ***	0.970 ***	

*** *p* = 0–0.001, ** *p* = 0.01, * *p* = 0.05. Blue: relationships between the variances of the three traits. Yellow: show relationships between the stability and variance for the three traits. Light green: correlations between the stability of the three traits. Dark green: presents the correlation for the data.

**Table:** 

Genotype	DI %			FDK %			DON mg/kg		
	Mean	Variance	Stability	Mean	Variance	Stability	Mean	Variance	Stability
Genius	16.19	215.97	0.80	6.53	72.97	0.53	6.16	44.71	0.29
GK Bakony	30.38	529.55	1.36	5.23	147.96	0.67	7.46	40.32	0.26
GK Börzsöny	32.67	845.62	1.69	16.63	407.81	1.04	12.77	153.60	0.54
GK Pilis	16.38	105.90	0.43	7.10	162.51	0.76	13.31	204.57	0.63
GK Szilárd	25.96	324.39	1.08	14.83	187.45	0.84	16.16	258.51	0.70
GK Bagó	24.79	220.95	0.89	7.13	59.85	0.38	16.55	300.82	0.61
GK Megyer	15.98	165.97	0.62	7.28	125.16	0.69	18.01	566.25	1.04
GK Csillag	21.29	215.95	0.73	13.27	198.06	0.81	20.56	586.32	1.04
GK Magvető	21.63	223.90	0.87	20.54	690.43	1.75	20.87	758.07	1.20
GK Békés	37.13	898.64	1.78	16.25	634.89	1.01	22.24	922.53	1.32
GK Csanád	17.79	133.39	0.71	11.43	205.10	0.89	24.05	776.31	1.21
GK Szereda	21.92	451.80	0.47	14.25	382.20	1.13	24.99	901.94	1.24
GK Kolozs	25.67	331.45	1.12	20.98	595.66	1.58	26.28	1068.50	1.42
GK Déva	20.46	285.74	0.99	14.00	347.65	1.22	27.49	1070.21	1.41
Altigo	32.17	499.97	1.48	22.75	672.80	1.72	38.22	2461.90	2.08
Mean	24.03	363.28	1.00	13.21	326.03	1.00	19.68	674.30	1.00
LSD 5%	7.28			6.69			7.45		

Dark green: lower than 50% of mean; Light green: 51–100% of mean; Yellow: 101–150% of mean; Orange: All above 150%.

**Table:** 

Trait	Sub-Trait	DI %			FDK %			DON mg/kg	
Correlations		Mean	Variance	Stability	Mean	Variance	Stability	Mean	Variance
DI	Variance	0.894 ***							
	Stability	0.919 ***	0.856 ***						
FDK	Mean	0.461	0.360	0.430					
	Variance	0.539 *	0.489	0.503	0.906 ***				
	Stability	0.278	0.196	0.266	0.91 ***	0.912 ***			
DON	Mean	0.191	0.052	0.106	0.726 **	0.652 **	0.719 **		
	Variance	0.277	0.134	0.229	0.707 ***	0.690 **	0.736 **	0.945 ***	
	Stability	0.181	0.075	0.128	0.744 ***	0.719 **	0.763 ***	0.979 ***	0.951 ***

*** *p* = 0–0.001, ** *p* = 0.01, * *p* = 0.05. Yellow: Correlations between trait means and variance and stability for the same trait; Blue: relation between the variances to the three traits; Light green: Relations between stability of the three traits, and Dark green: correlations between the three traits.

## Data Availability

The original contributions presented in this study are included in the article/Supplementary Materials. Further inquiries can be directed to the corresponding author(s).

## Supplementary Materials

The following supporting information can be downloaded at https://www.mdpi.com/article/10.3390/toxins17060288/s1, Table S1. Disease index, % of the infected spikelets relative to the total number of spikelets. Basic data of the genotype reactions to FHB resistance tests against four *Fusarium graminearum* and *F. culmorum* isolates, 1990–1993 [14]; Table S2. Correlation analysis of the data in Table 1 showing the genotype correlations between epidemics, 1990–1993 [14]; Table S3. Basic data of the genotype reactions to FHB resistance tests against four *F. graminearum* and *F. culmorum* isolates, 1990–1993. FDK, % of the visually infected grains from the inoculated heads [14]; Table S4. Correlation analysis of the FDK data in Table 3 showing the genotype correlations between epidemic situations, 1990–1993 [14]; Table S5. Basic data of the genotype reactions to FHB resistance tests against four F. graminearum and F. culmorum isolates. DON contamination, mg/kg, in the grains from the inoculated heads, 1990–1993 [14]; Table S6. Correlation analysis of the DON data of Table 5 showing the genotype correlations between epidemic situations, 1990–1993 [14]; Table S7. Wheat FHB resistance tests and summary of ranks of means, variances, and stability indices for FHB traits DI, FDK, and DON, 1990–1993 [14]; Table S8. FHB resistance tests in winter wheat genotypes, 1994–1996. Disease index in 24 epidemic situations to isolates of *F. graminearum* and *F. culmorum* [14]; Table S9. Ranks of the genotypes in the FHB resistance tests, 1990–1993. Disease index in 24 epidemic situations and 25 genotypes [14]; Table S10. Correlation between responses of genotypes in different epidemic situations based on Table S8, 1990–1993 [14]; Table S11. FDK, % of the visually infected grains from the inoculated heads. Basic data of the genotype reactions to FHB resistance tests against four *F. graminearum* and *F. culmorum* isolates, 1994–1996 [14]; Table S12. Correlation analysis of the FDK data in Table 3 showing the genotype correlations between epidemic situations, 1994–1996 [14]; Table S13. DON, mg/kg, in the infected grains from the inoculated heads. Basic data of the genotype reactions to FHB resistance tests of winter wheat genotypes against four *F. graminearum* and *F. culmorum* isolates, 1994–1996 [14]); Table S14. Correlation analysis of the DON data of Table 5 showing the genotype correlations between epidemic situations, 1994–1996 [14]; Table S15. Correlation analysis of the DON data of Table 5 showing the correlations between epidemic situations across genotypes, 1994–1996 [14]; Table S16. Ranks of the FHB resistance tests for DI, FDK, and DON contamination with variance and SI data and their correlations, 1994–1996 [14]; Table S17. Winter wheat resistance test of 40 genotypes to FHB in 36 epidemic situations, 2009–2012. Disease index (%) [24]; Table S18. Wheat resistance test for resistance to FHB. Correlation analysis of DI data of Table S17 showing the epidemic correlations across genotypes, 1994–1996 [24]; Table S19. Winter wheat resistance test of 40 genotypes to FHB resistance in 36 epidemic situations, FDK %, 2009–2012 [24]; Table S20. Wheat resistance test for resistance to FHB. Correlation analysis of FDK data of Table S19 showing the epidemic correlations across genotypes, 2009–2012 [24]; Table S21. Winter wheat resistance test of 40 genotypes to FHB in 36 epidemic situations, 2009–2012. Deoxynivalenol (DON) contamination, mg/kg [24]; Table S22. Wheat resistance test for resistance to FHB. Correlation analysis of DON data of Table S21 showing the epidemic correlations across genotypes, 2009–2012 [24]; Table S23. Summary of the 2009–2012 FHB resistance tests for DI, FDK, and DON. FHB resistance rankings based on Table 4 [24]; Table S24. Winter wheat resistance tests against FHB. Disease index data for genotypes and years and inocula used, 2019–2021; Table S25. Winter wheat resistance tests against FHB. FDK data (%) for genotypes, and years and inocula used, 2019–2021; Table S26. Winter wheat resistance tests against FHB. DON data, mg/kg, for genotypes and years and inocula used, 2019–2021. Figure S1. Stability analysis for DI (A–E), 1990–1993; Figure S2. Stability analysis for FDK (A–E), 1990–1993; Figure S3. Genotype responses to DI in 32 epidemics, 1990–1993; Figure S4. Genotype responses to FDK in 32 epidemics, 1990–1993; Figure S5. Stability analysis for DI (A–E), 1990–1993; Figure S6. Stability analysis for FDK (A–E), 1990–1993; Figure S7. Genotype responses to DI in 24 epidemics, 1990–1993; Figure S8. Genotype responses to FDK in 242 epidemics, 1990–1993; Figure S9. Stability analysis for DI (A–E), 1990–1993; Figure S10. Stability analysis for FDK (A–E), 1990–1993; Figure S11. Genotype responses to DI in 36 epidemics, 1990–1993; Figure S12. Genotype responses to FDK in 36 epidemics, 1990–1993; Figure S13. Stability analysis for DI (A–E), 1990–1993; Figure S14. Stability analysis for FDK (A–E), 1990–1993; Figure S15. PCA of the winter wheat FHB resistance tests for DI, FDK, and DON contamination for genotypes and epidemics (A–H), 1990–1993; Figure S16. PCA of the winter wheat FHB resistance tests for DI, FDK, and DON contamination for genotypes and epidemics (A–H), 2009–2012; Figure S17. PCA of the winter wheat FHB resistance tests for DI, FDK, and DON contamination for genotypes and epidemics (A–H), 2009–2012; Figure S18. PCA of the winter wheat FHB resistance tests for DI, FDK, and DON contamination for genotypes and epidemics (A–H), 2019–2021.

## Abbreviations

ANOVA	Analysis of variance
AUDPC	Area under disease progress curve
DH	Doubled haploid
DI	Disease index
DON	Deoxynivalenol
FDK	Fusarium-damaged kernels
FHB	Fusarium head blight
GK	Gabona Kutató (Cereal Res. Ltd.)
LSD	Limit of smallest difference
PCA	Principal component analysis
PCR	Polymerase chain reaction
QTL	Quantitative trait locus
RD	Resistance to DON accumulation
SI	Stability index; b value in the linear function y = a + bx
