# Supplementary material for: Significance of the Stability of Fusarium Head Blight Resistance in the Variety Registration, Breeding, and Genetic Research of Winter Wheat Using Disease Index, Fusarium-Damaged Kernels, and Deoxynivalenol Contamination
Source: Toxins (Basel). 2025 Jun 6;17(6):288. doi: 10.3390/toxins17060288 (PMC12197506; doi:10.3390/toxins17060288)
Supplement: Supplementary file 1 [file toxins-17-00288-s001.zip › toxins-3532949-supplementary.pdf]

Supplementary Materials: Significance of the Stability of Fusarium Head Blight resistance in the Variety Registration, Breeding, and Genetic Research of Winter Wheat Using Disease Index, Fusarium-Damaged Kernels, and Deoxynivalenol Contamination

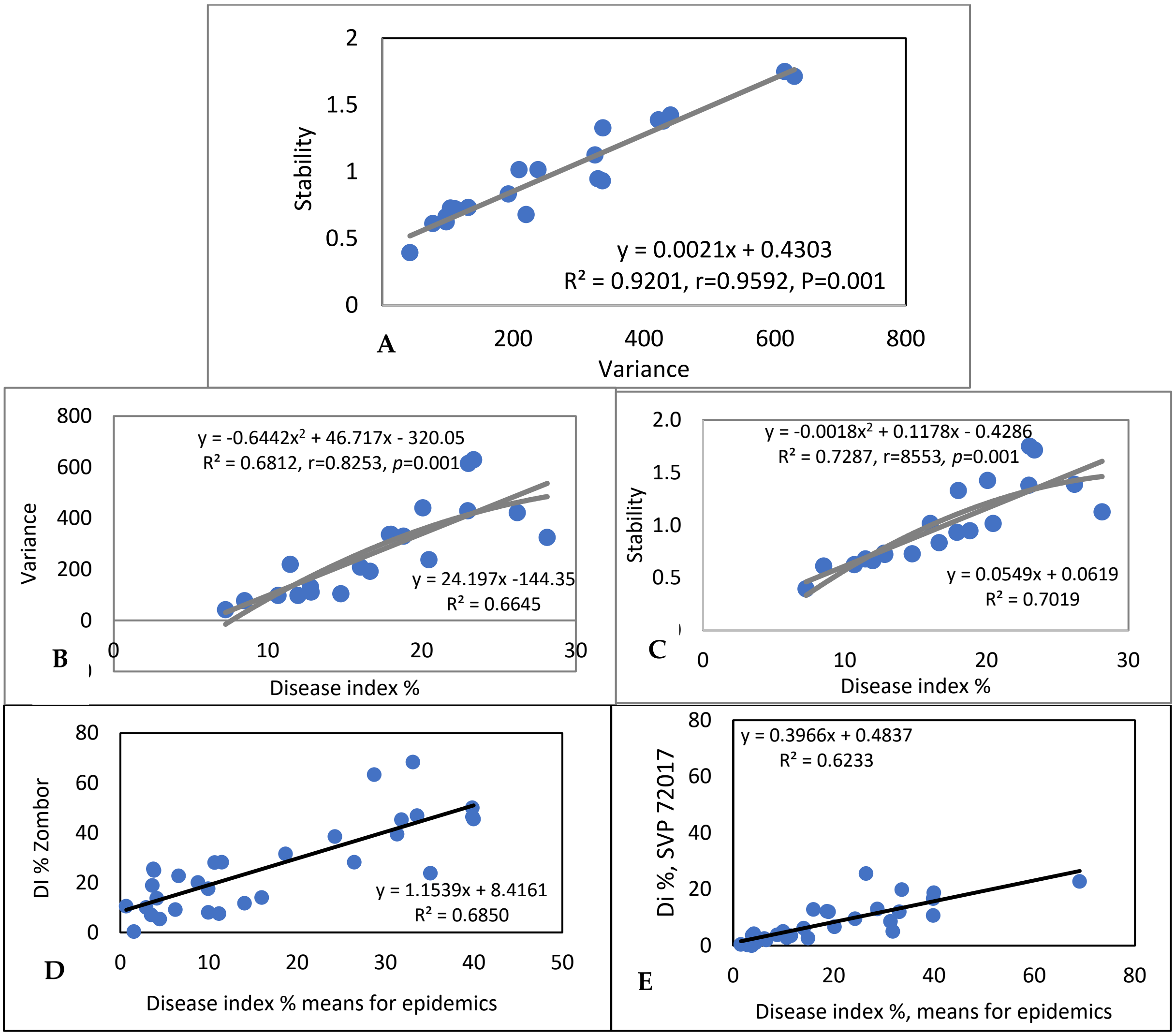

Figure S1. Comparison between the variance and stability for the 20 winter wheat genotypes and the disease index (DI) and variance and stability indices (SI) using linear and polynomial functions. (**A**) Regression between the variance and stability index. **(B**) Regression between the DI of wheat genotypes and variance. (**C**) Regression between DI and SI. (**D**) The DI and SI of the most susceptible genotype; the high "b" value (1.15) shows a high and very variable performance. **(E)** the most resistant genotype shows a low b value, b=0.3966, indicating a much better adaptation to different conditions.

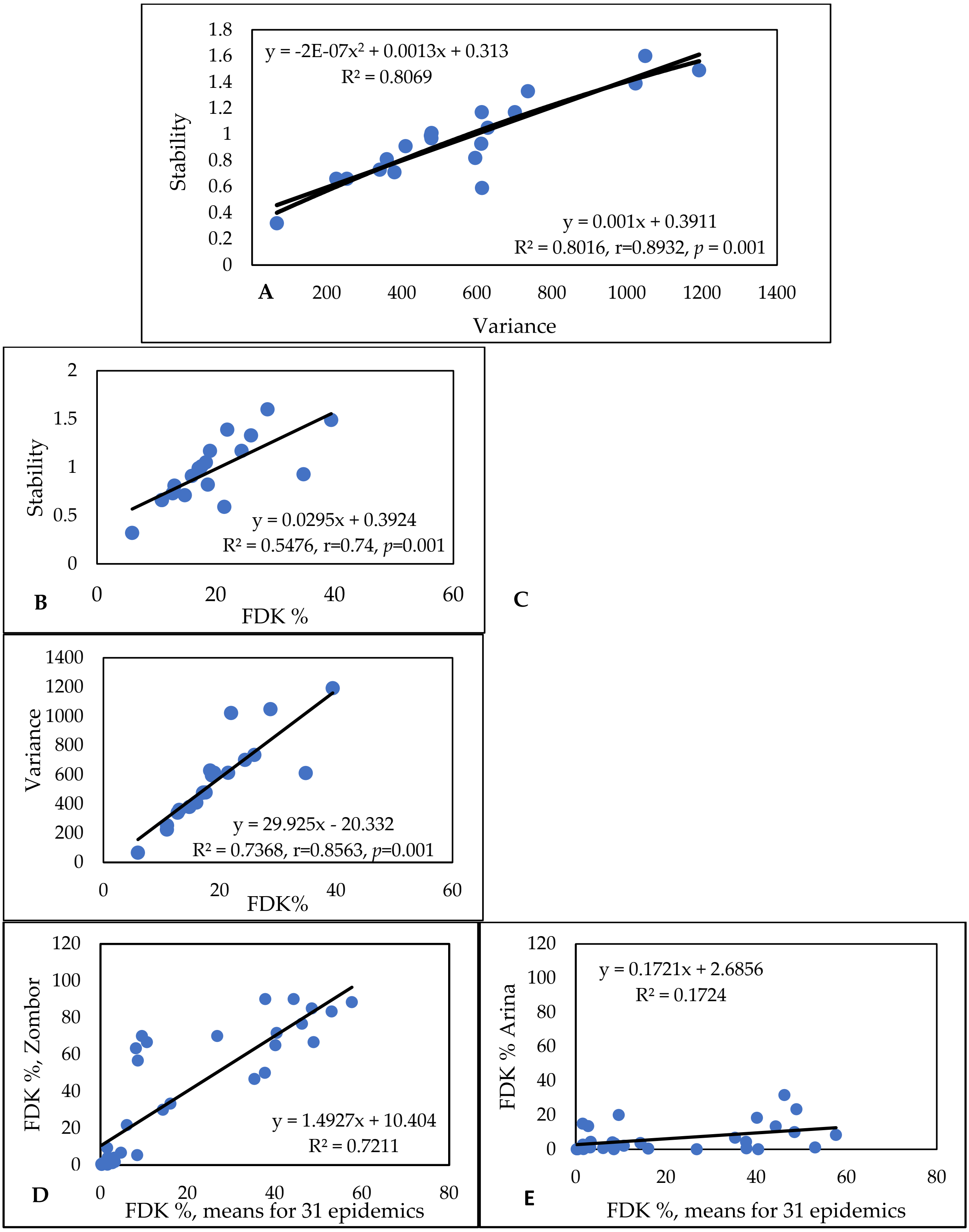

Figure S2. Comparison between FDK data for variance and stability indices for the 20 genotypes. (A) Comparison of the two stability traits. (B) Regression for FDK mean and SI (b value). (C) Regression between FDK means and variance. (D) Unstable, highly susceptible cultivar (b=1.49). (E) Good stability and resistance (b=0.1721).

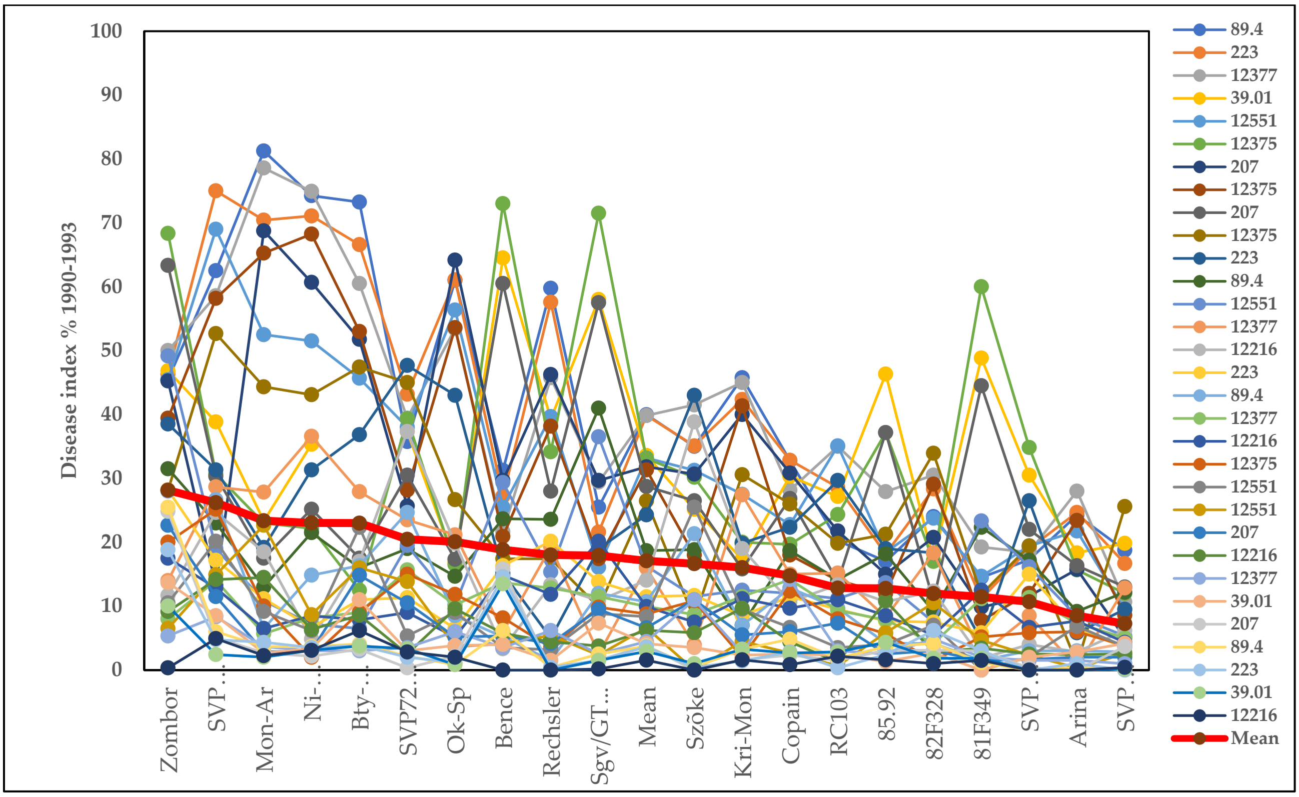

Figure S3. Response of winter wheat genotypes to FHB, visible ear infection, and disease index in 31 epidemics. The thick red line indicates the means of the 31 epidemics.

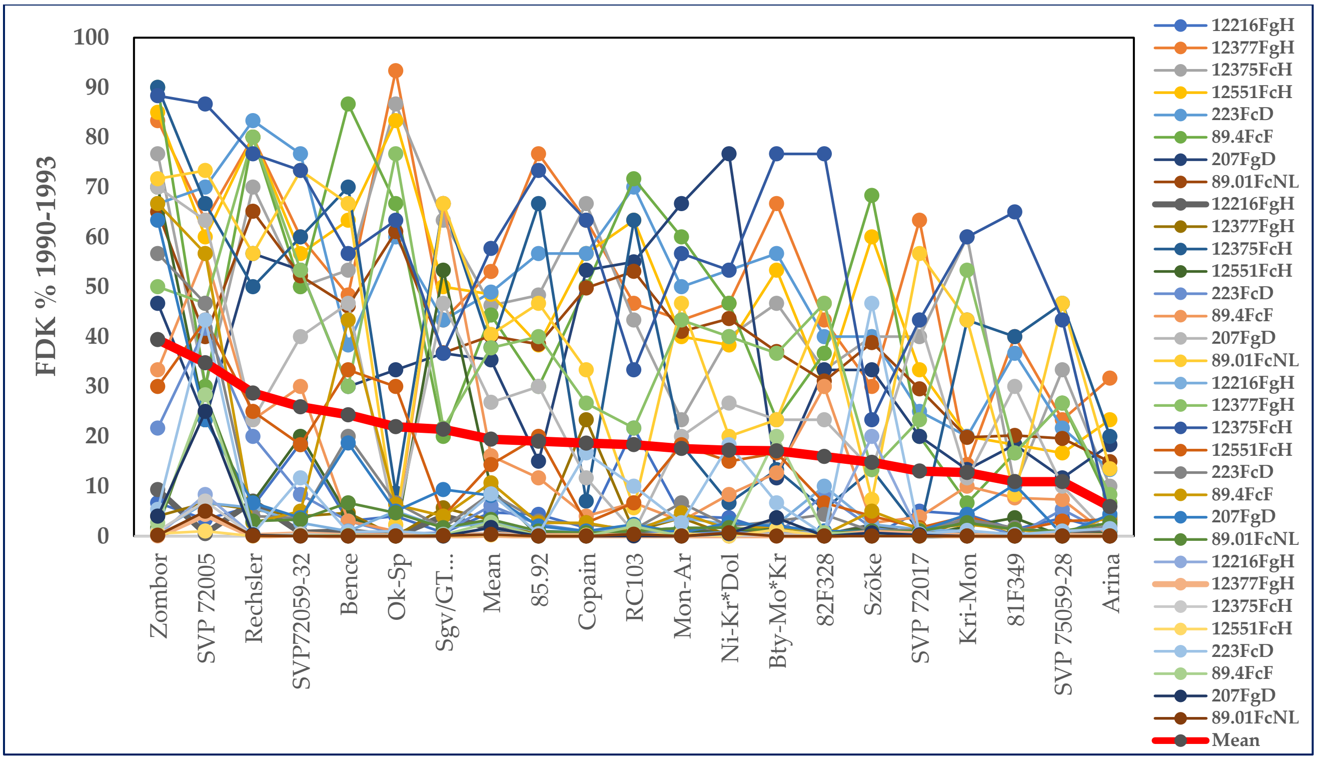

Figure S4. Response of winter wheat genotypes to FDK and visible grain infection in 31 epidemics. The thick red line indicates the means of the 31 epidemics.

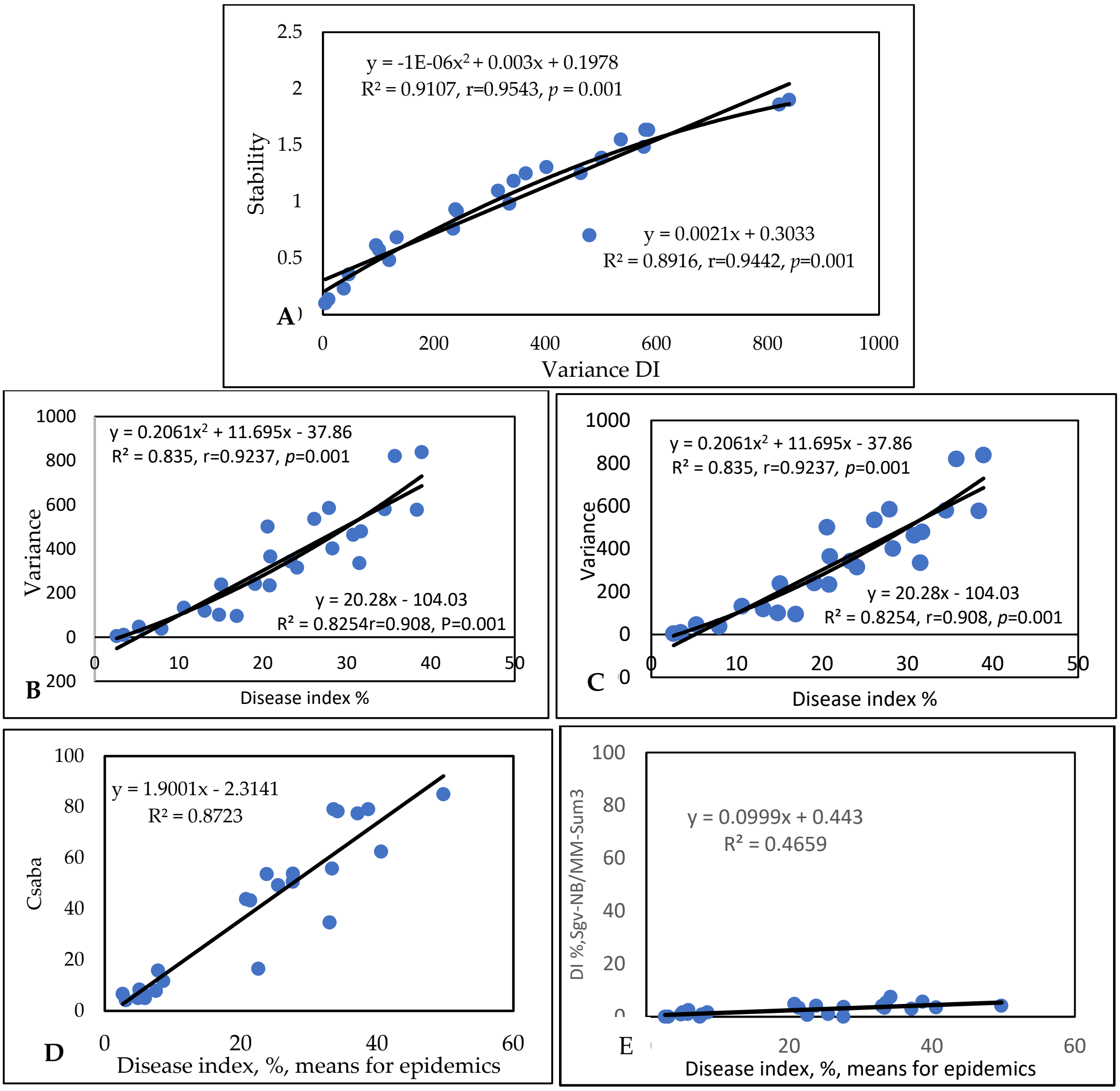

Figure S5. Comparison between the variance and stability for the 20 genotypes and the disease, variance, and stability indices. (**A**) Comparison of the two stability traits**. (B)** Regression for DI means and stability. (**C**) Regression between DI means and stability. Data from study conducted in 1994–1996. (**D**) SI values of the most susceptible genotypes. **(E**) The most resistant genotype is from the combination Sgv-NB/MM-Sum3 containing both Nobeoka Bozu and Sumai3 in its pedigree.

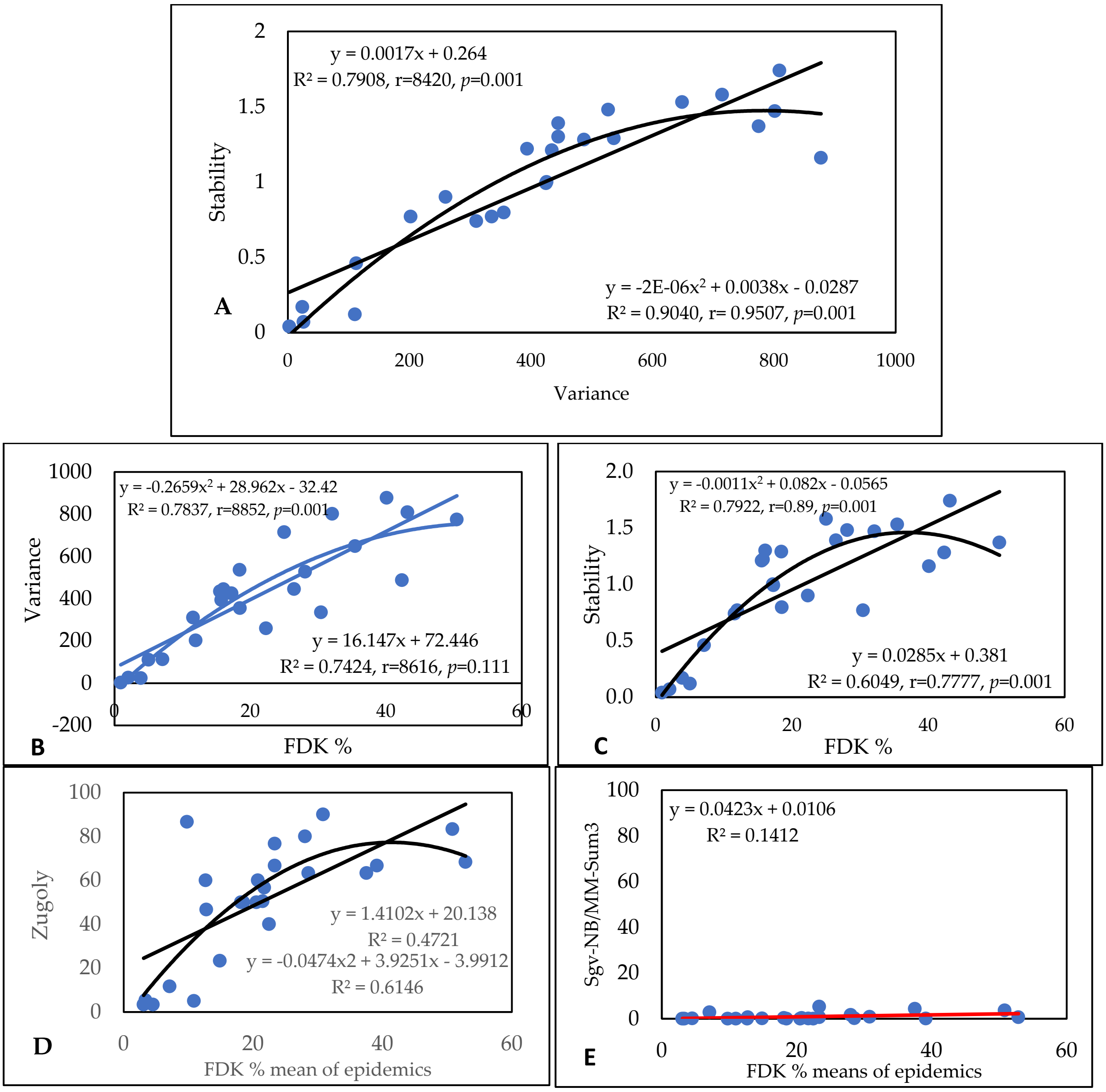

Figure S6. Comparison between the variance and stability for the 25 genotypes for the FDK. (**A**) Comparison of the two stability traits. (B) Regression for FDK means and variance. (**C**) Regression between FDK means and stability. (**D**) Highly susceptible, unstable performance; the b value is very high at 1.9001. (**E**) Highly resistant, with a very low infection rate under all disease conditions; the b value is extremely low (0.04), therefore, the regression line is red to differentiate it from the x axis. Data from study conducted in 1994–1996.

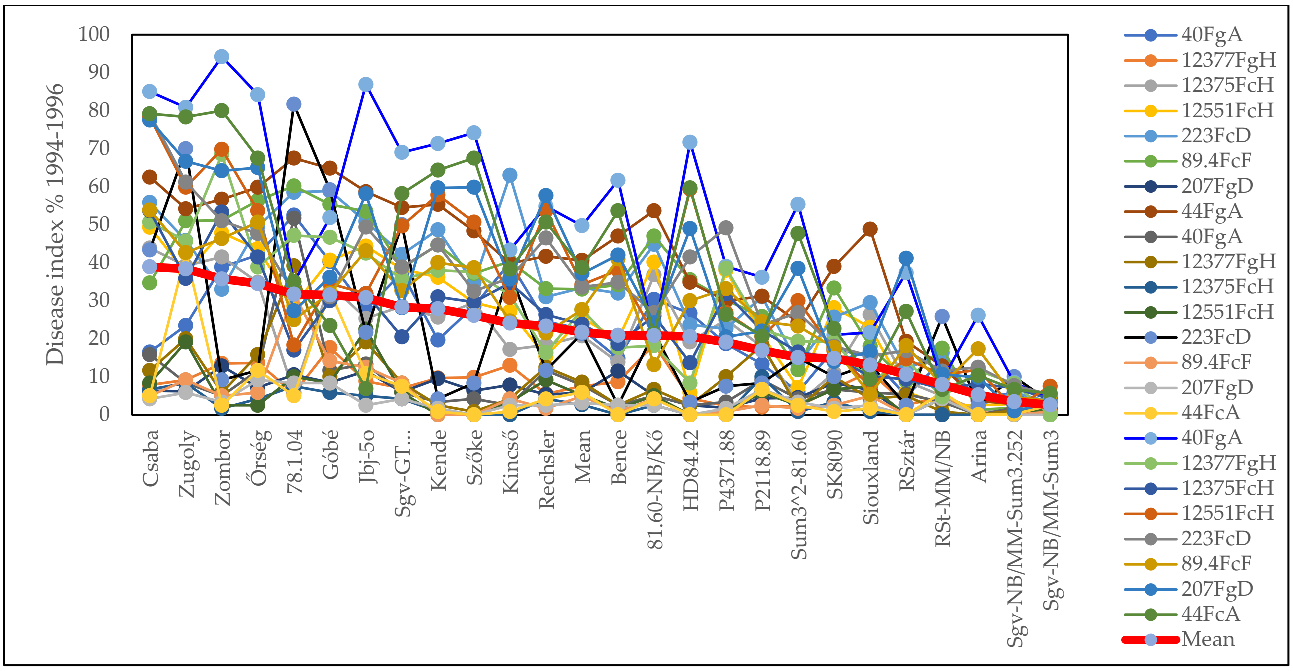

Figure S7. Responses of 25 winter wheat genotypes to FHB; visible infection (DI) in 24 epidemics. The thick red line indicates the means of the 24 situations (1994-1996).

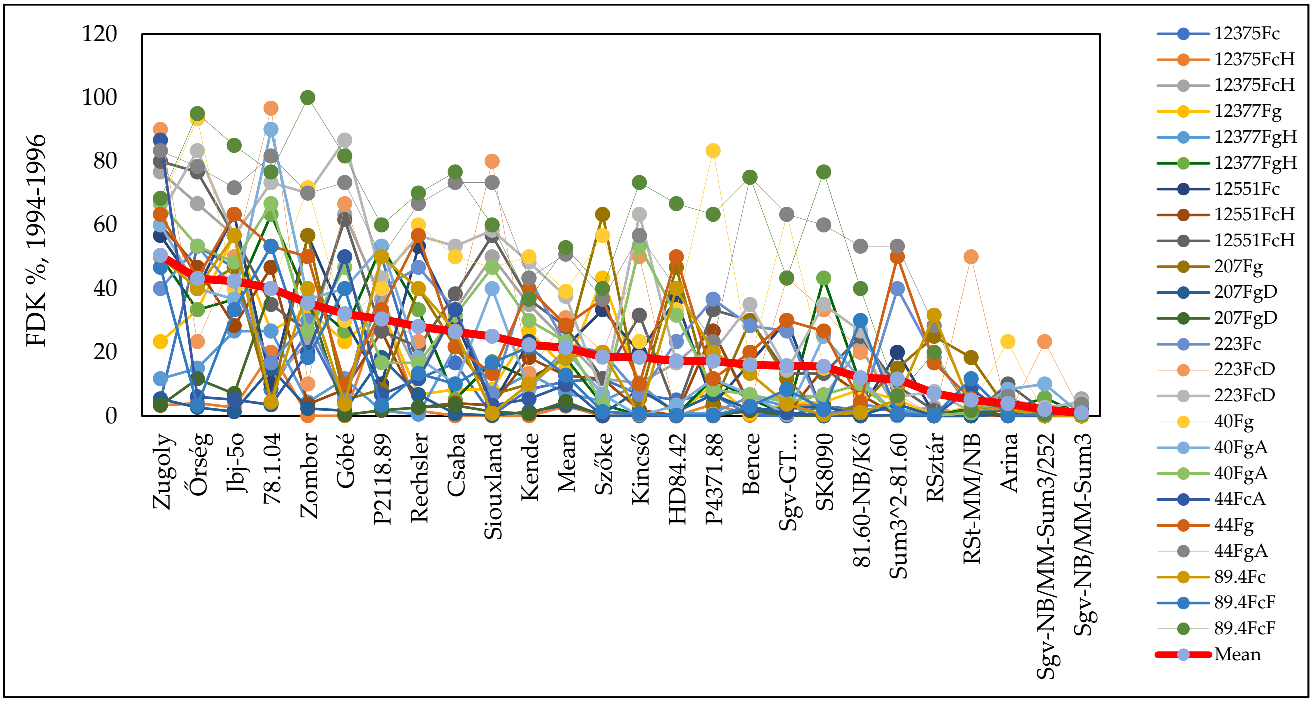

Figure S8. Responses of 25 winter wheat genotypes to FDK; visible infection (DI) in 24 epidemic situations. The thick red line indicates the means of the 24 epidemics (1994-1996).

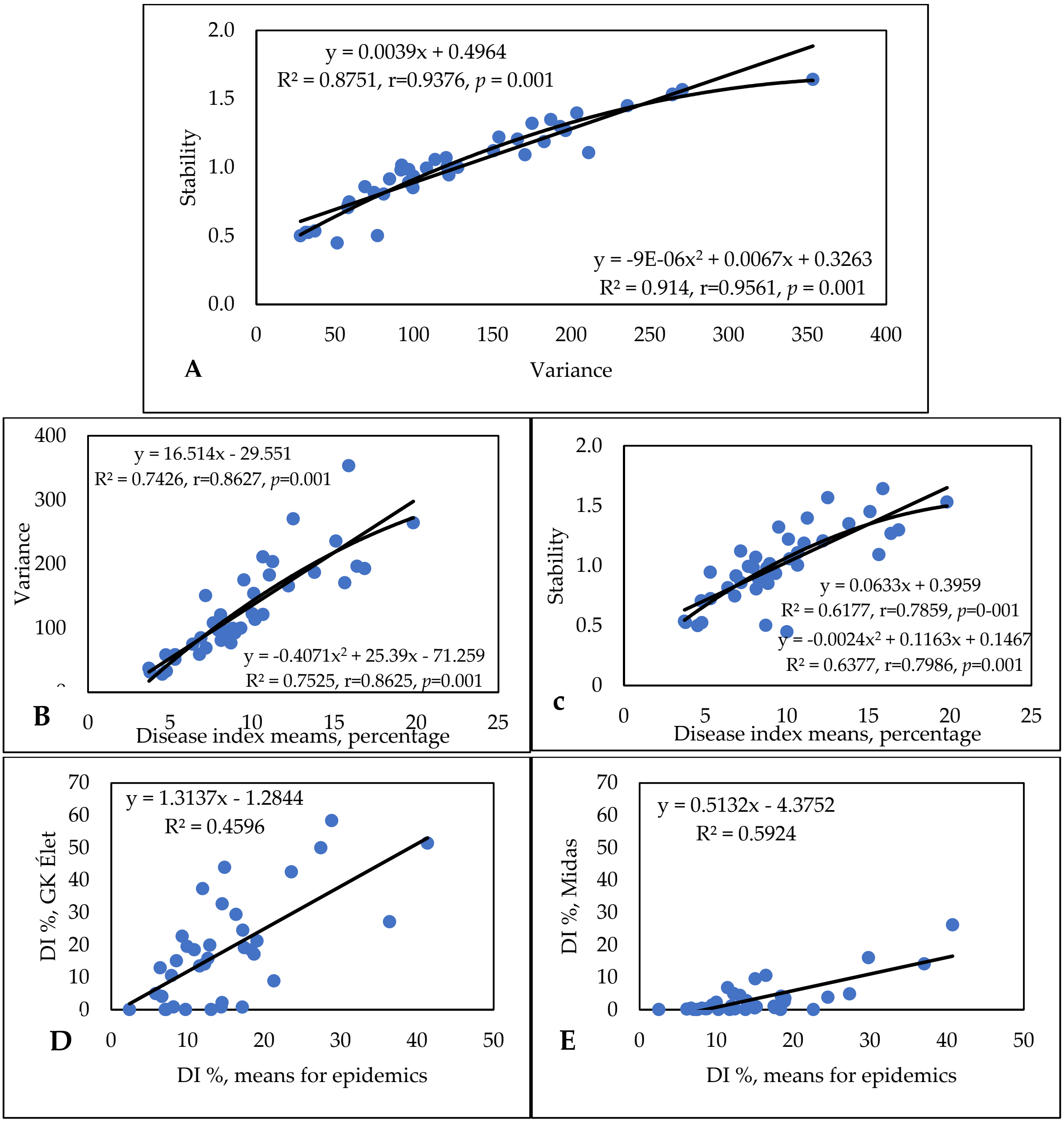

Figure S9. Resistance testing of 40 genotypes in 36 epidemic situations, determining stability according to the variance and stability indices [26] (**A**). Disease index data. Stability data are given between genotype resistance **(B)** and stability traits (**C**). Stability test of the susceptible genotype, GK Elet (**D**), shows a high rise in the line with large variations in the data (b=1.3137). The more resistant genotype, Midas, is more stable (b=0.5112) (**E**); the data are closer to the x axis than they are for the GK Elet genotype (2009-2012).

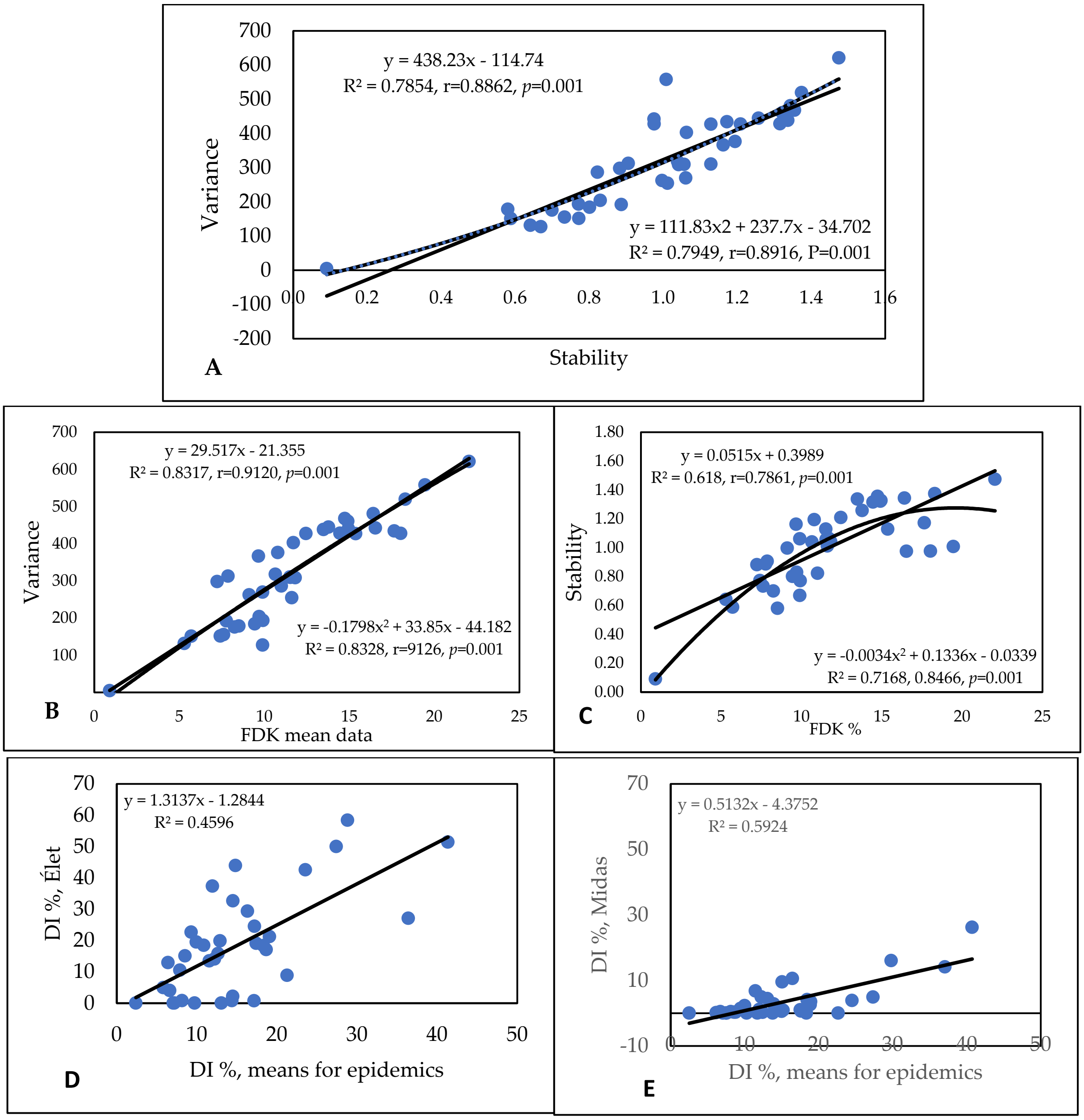

Figure 10. Resistance testing of 40 genotypes in 36 epidemic situations for stability of FDK according to variance and stability indices (**A**). Stability data are given between genotype resistance and stability traits (**B**) and (**C**). However, the stability data here follow a more polynomial trend. The highly susceptible cv genotype (**D**) showed high variability and high upper data, but the more resistant genotype provided much lower variability and more followable behavior (**E**) (2009-2012).

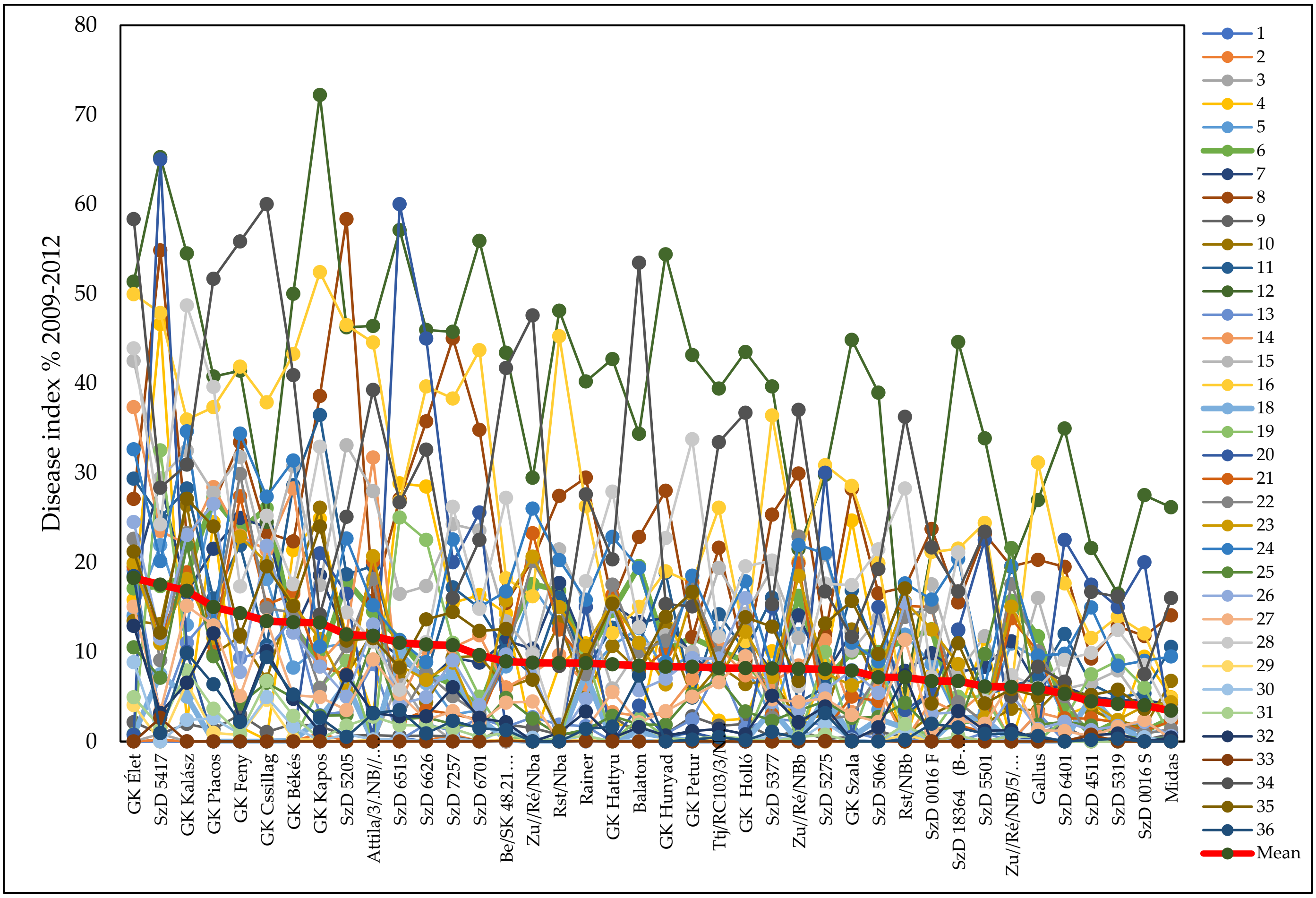

Figure S11. Disease index (%) of 40 winter wheat genotypes in 36 epidemics (four isolates, three methods, and four years) over 2009–2012 [24]. The red thick line shows the means across 36 epidemic means

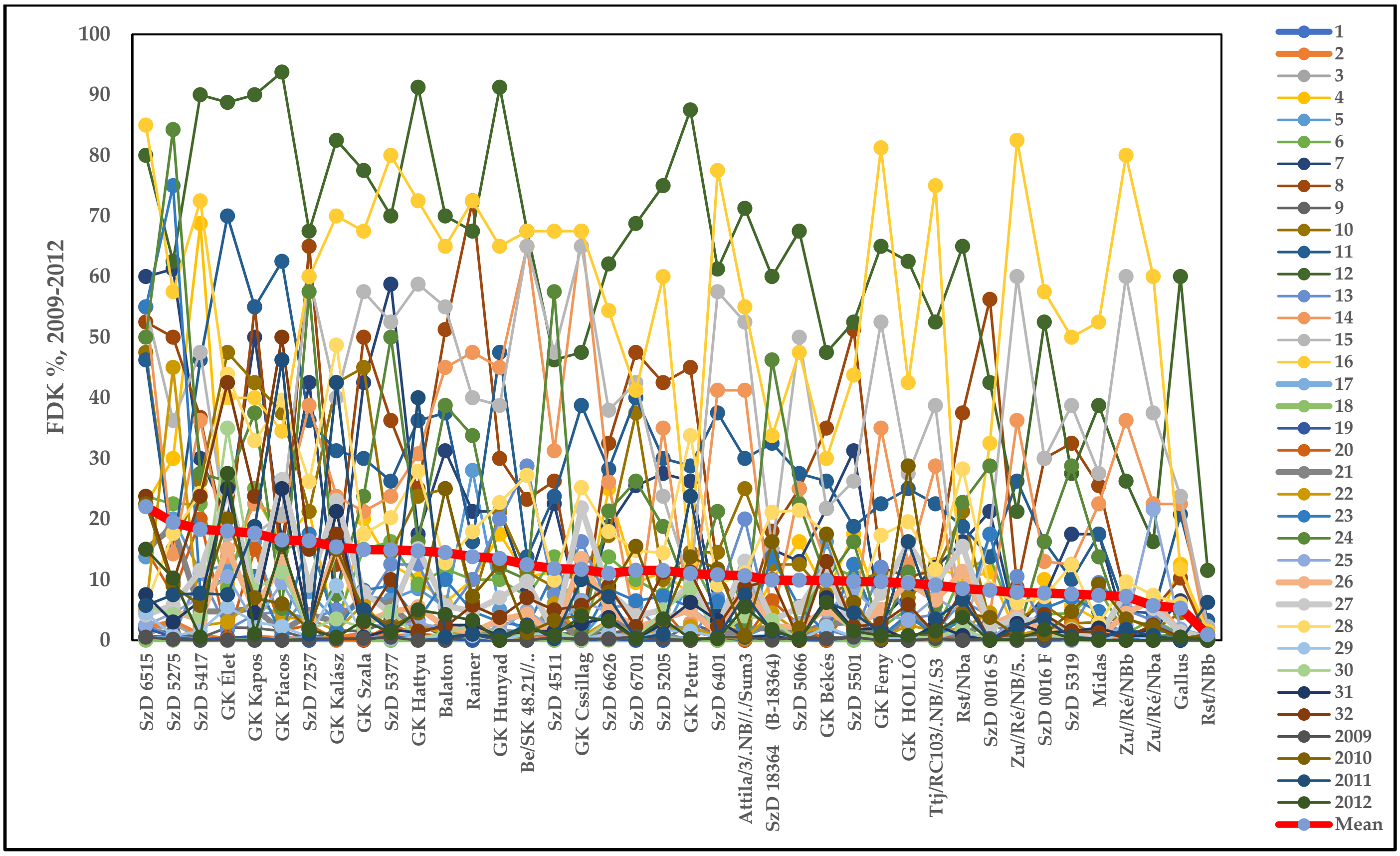

Figure S12. FDK % of 40 winter wheat genotypes in 36 epidemics (four isolates, three methods, and four years) over 2009–2012. Original paper [24].

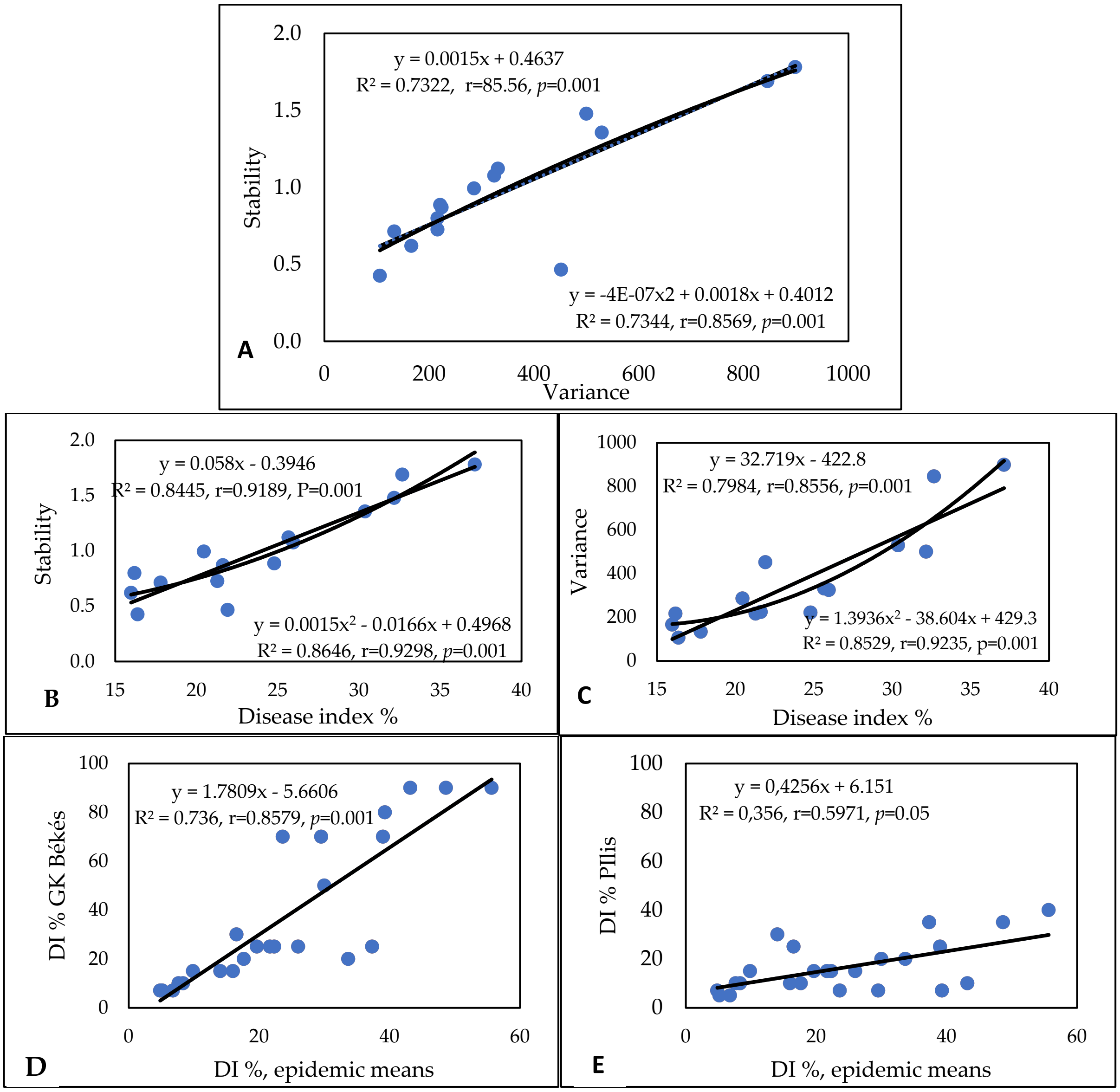

Figure S13. Comparison between variance and stability for the 15 varieties; the disease index and the variance and stability indices, using data obtained in 2019–2022. (**A**) Regression between variance and stability indices. (**B**). Regression between DI and SI (**C**). Stability indices for susceptible (**D**) and moderately resistant genotypes (**E**), respectively.

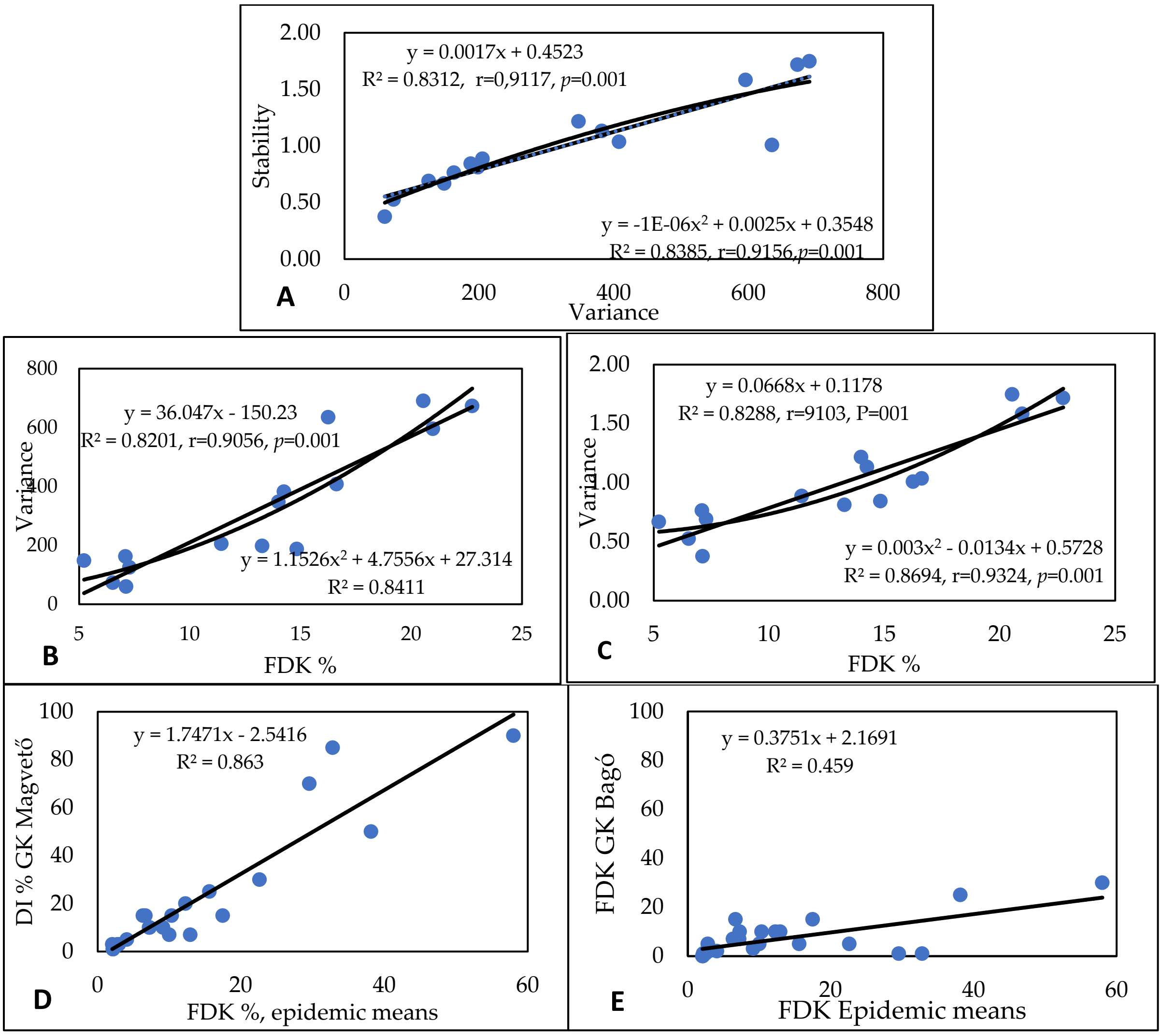

Figure S14. Comparison between variance and stability for the 10 varieties and the FDK, for data obtained in 2019–2022. (**A**) Regression between variance and stability indices. (**B**) Regression between DI and variance data. (**C**) Regression between DI and SI. Stability index for susceptible (**D**) moderately resistant **(E)** genotypes, respectively.

Figure S15 (A-G). PCA analyses of the winter wheat FHB resistance tests for DI, FDK and DON contamination at genotypes and epidemics, 1990-1993.

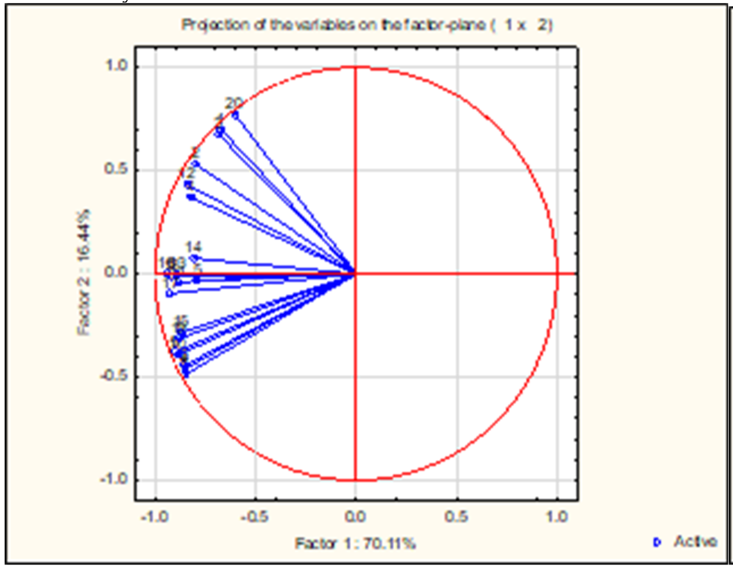

15 A. Disease index and genotypes (n=20)

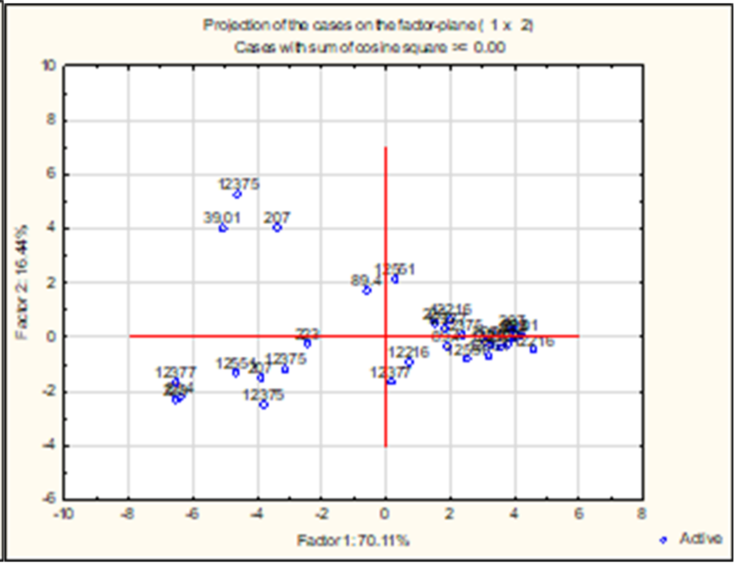

15 B. DI and 24 epidemics (isolates)

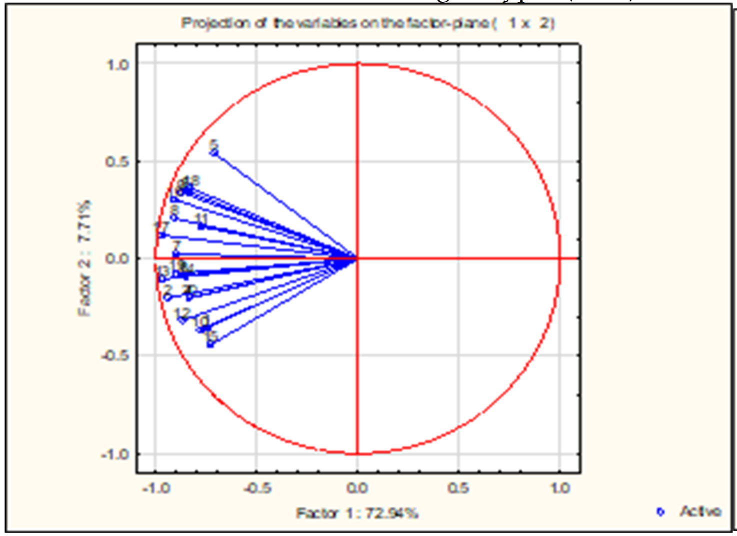

15C. FDK and genotypes

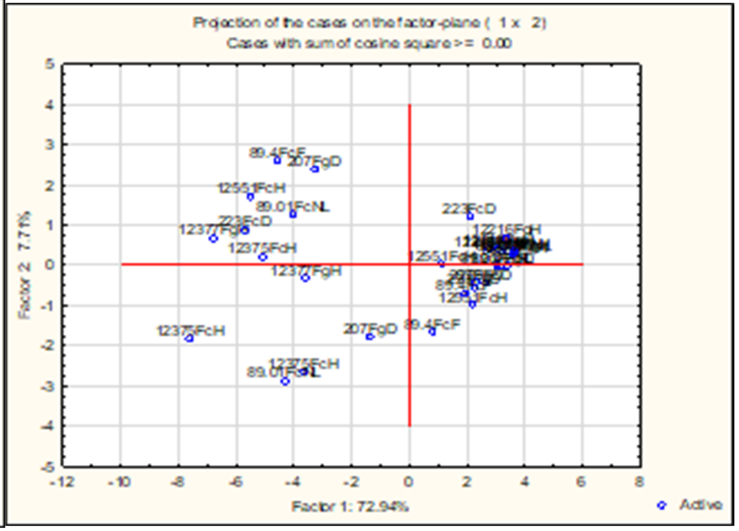

15D. FDK and 24 epidemics (isolates)

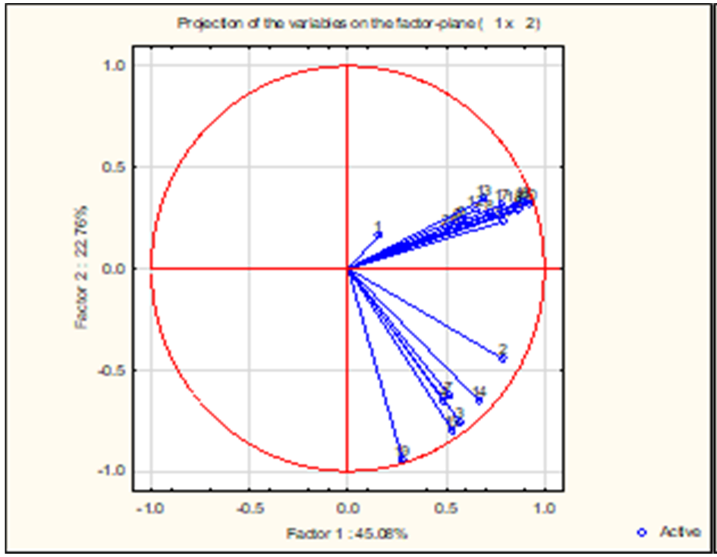

15E. DON and genotypes

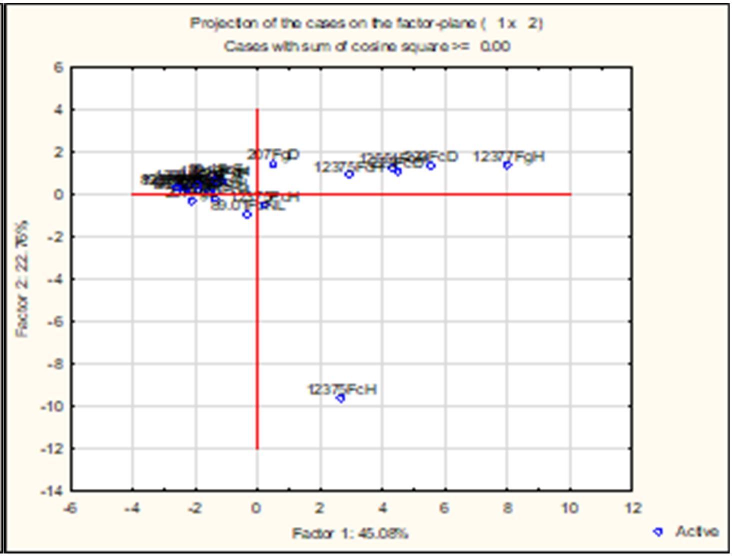

15F. DON and 24 epidemics

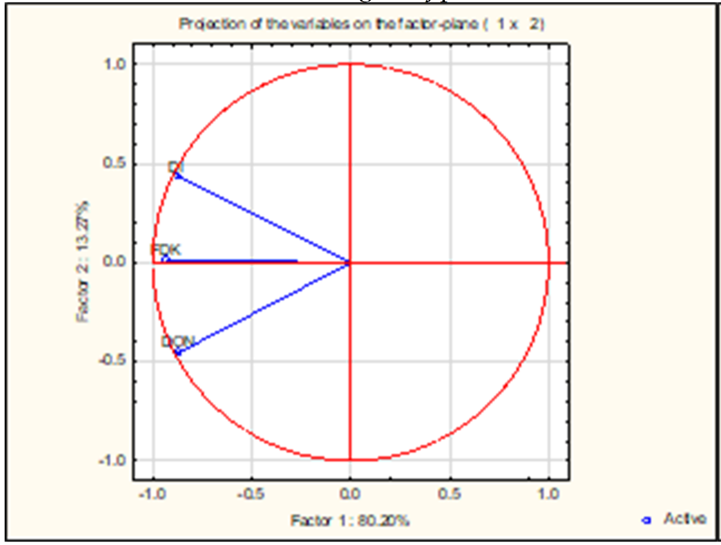

15G DI, FDK and DON together

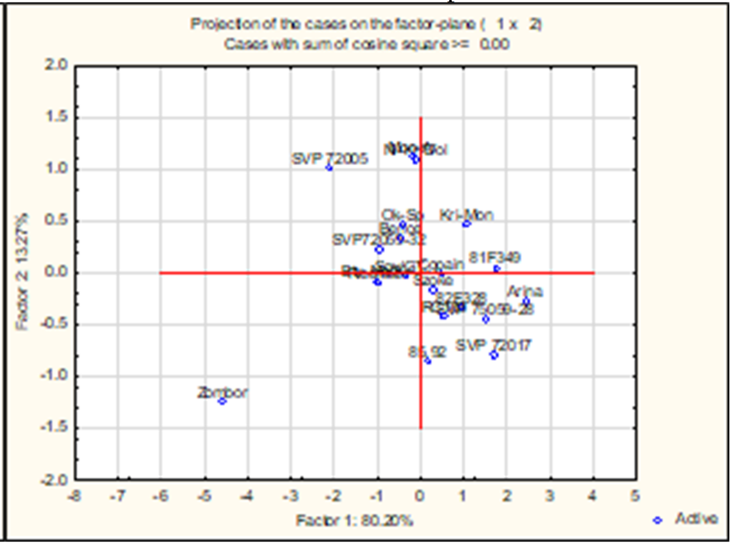

15H. Genotype positions across the three traits

Figure S16 (A-G). PCA analyses of the winter wheat FHB resistance tests for DI, FDK and DON contamination for genotypes and epidemics, 1994-1996.

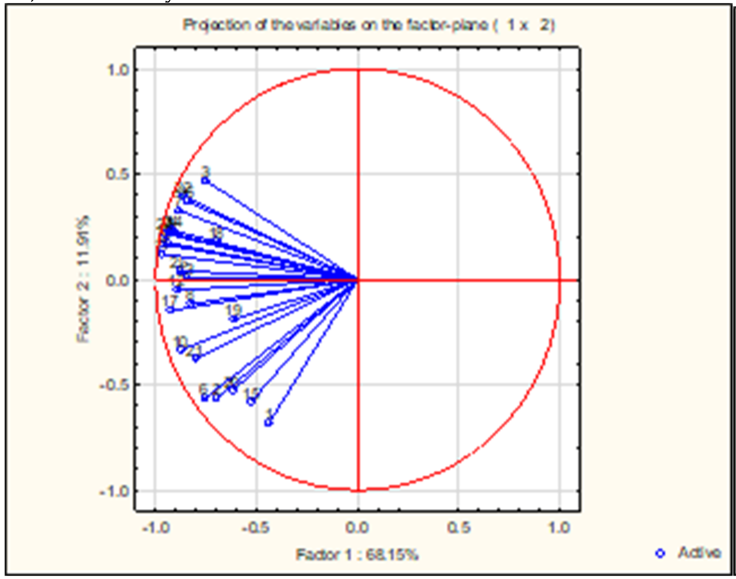

16A. Disease index genotype distributions (n=25)

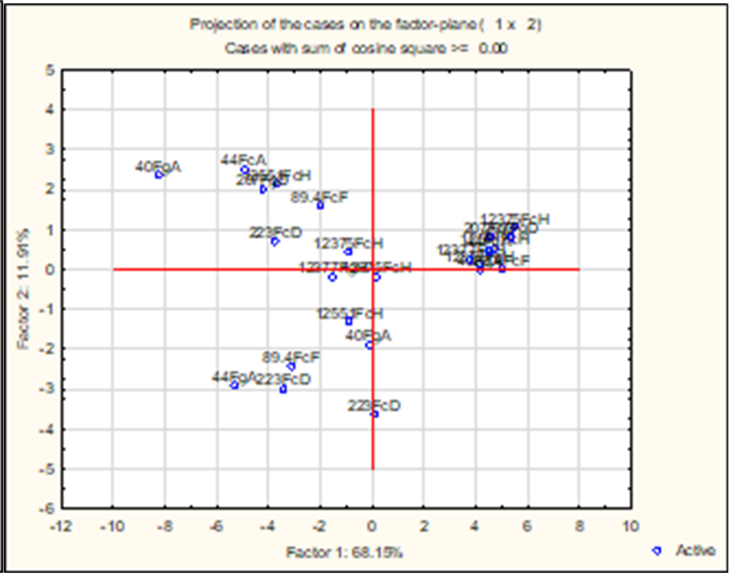

16B. DI and epidemics (n=24)

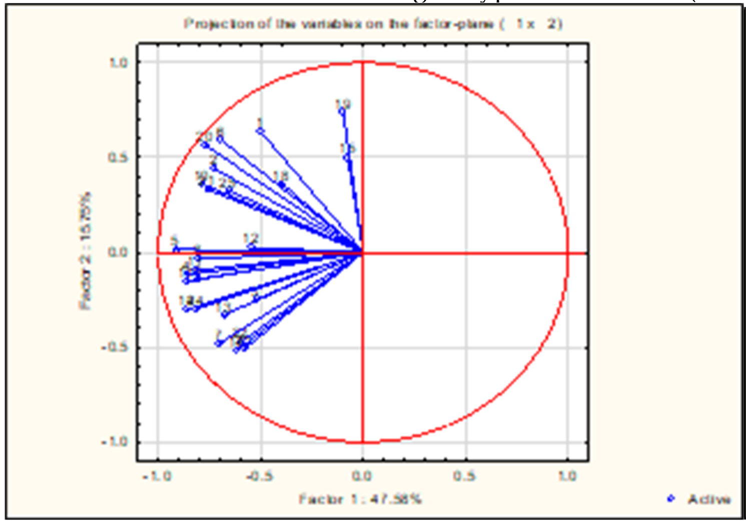

16C. FDK, genotype distributions (n=25)

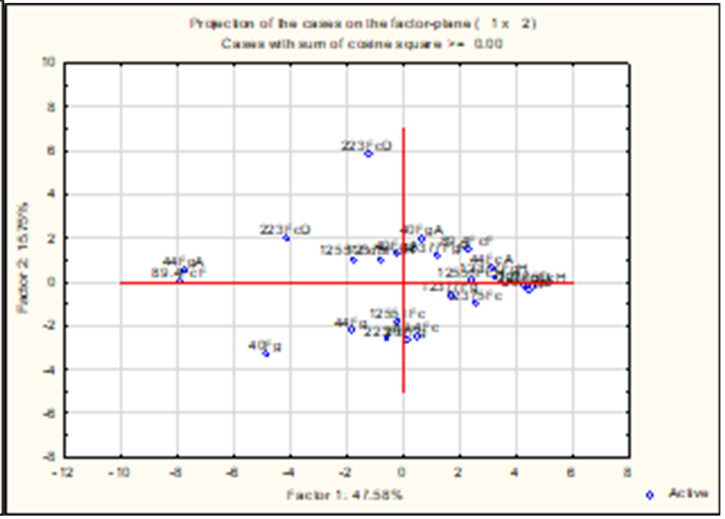

16D. FDK and epidemic distribution (n=24)

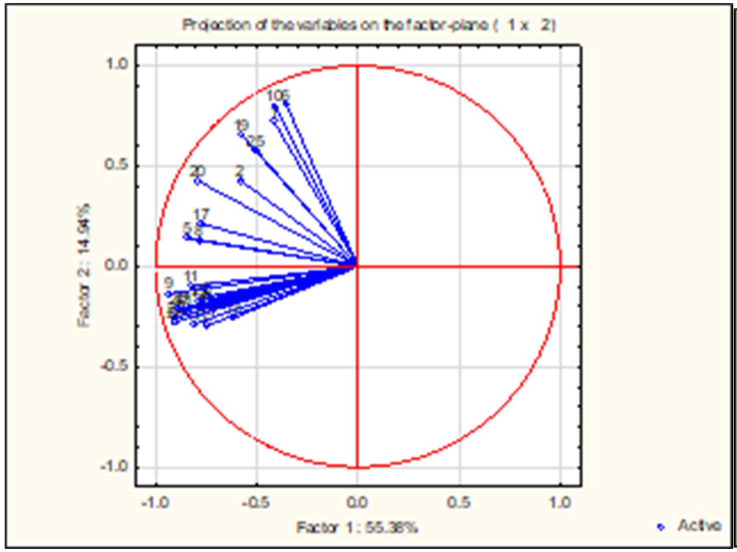

16E. DON and genotype distributions (n=25)

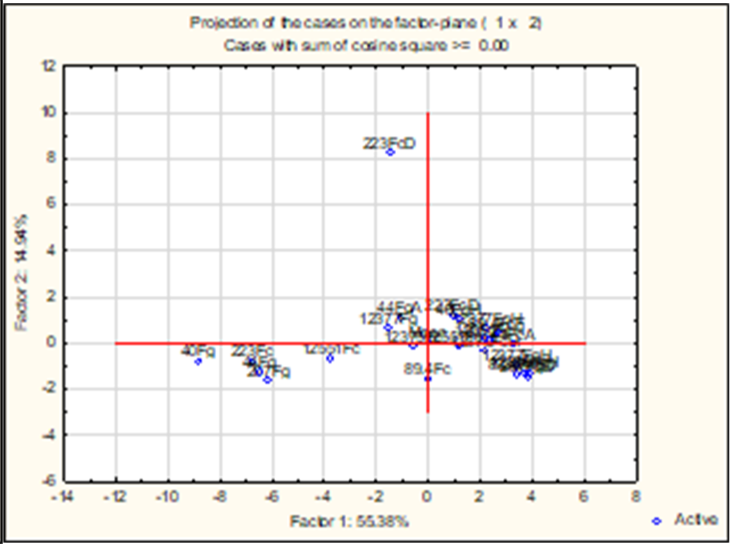

16F. DON and 24 epidemics distribution

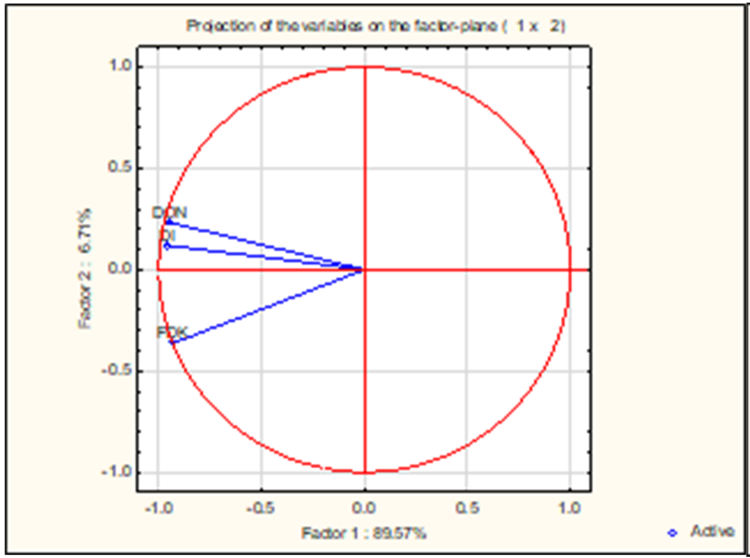

126G. Traits across genotypes DI, FDK and DON pooled

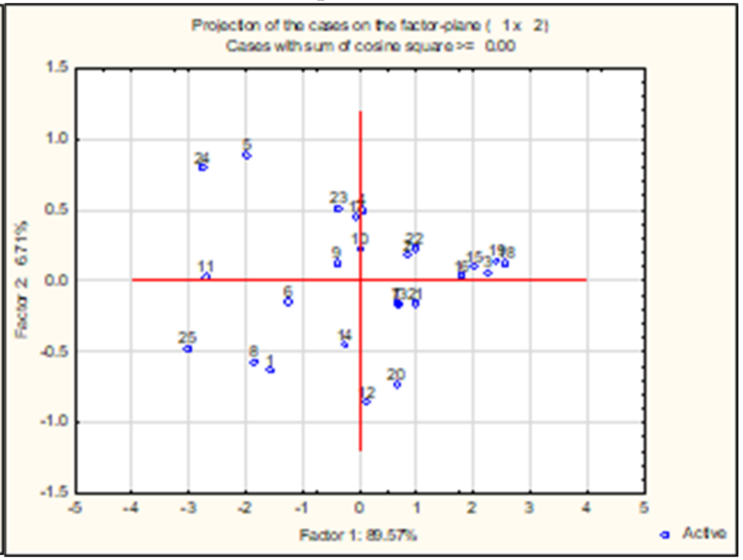

16H. Genotype positions across the three traits

Figure S17. PC analyses of the winter wheat FHB resistance tests for DI, FDK and DON contamination for genotypes and epidemics, 2009-2012.

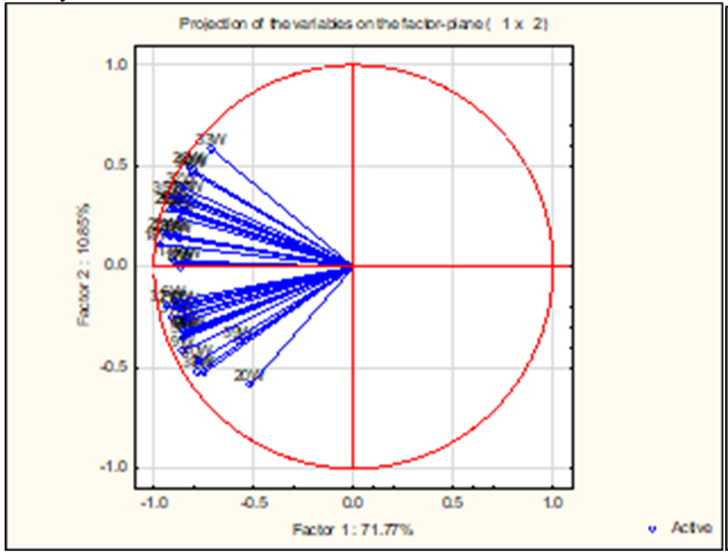

17A. Disease index, genotype distribution (n=40)

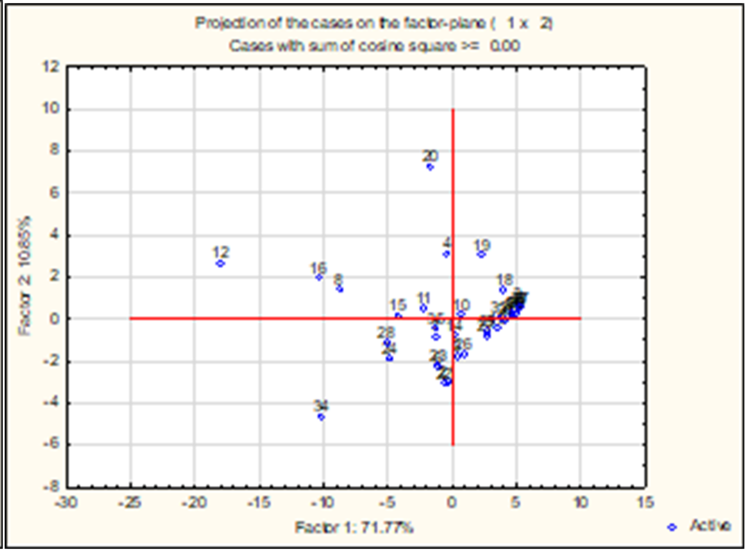

17B. Disease index, epidemic distribution, n=36)

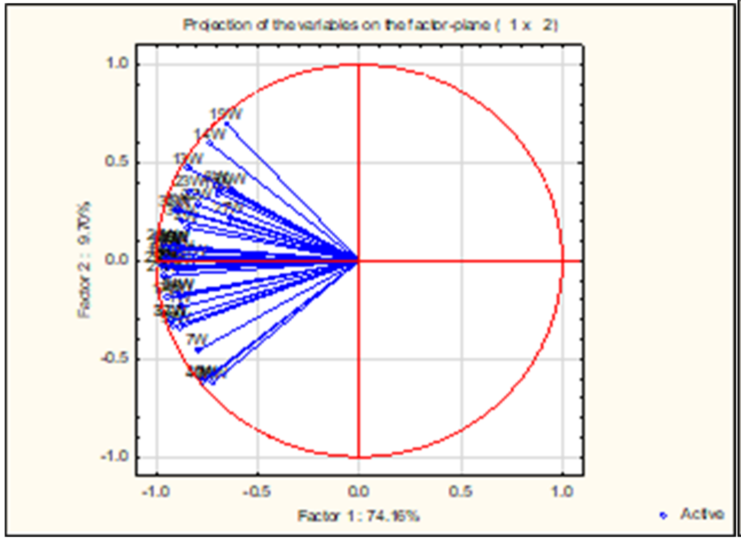

17C. FDK genotype distribution (n=40)

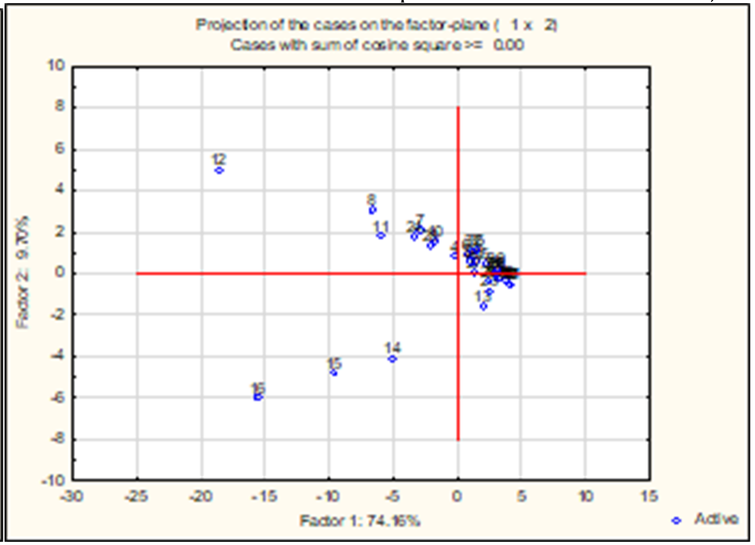

17D. FDK epidemic genotype positions (n=36)

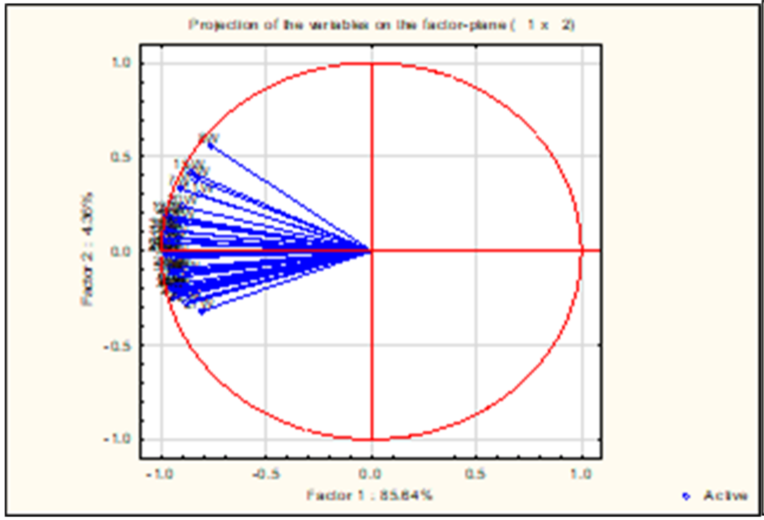

17E. DON and genotype distribution n=40

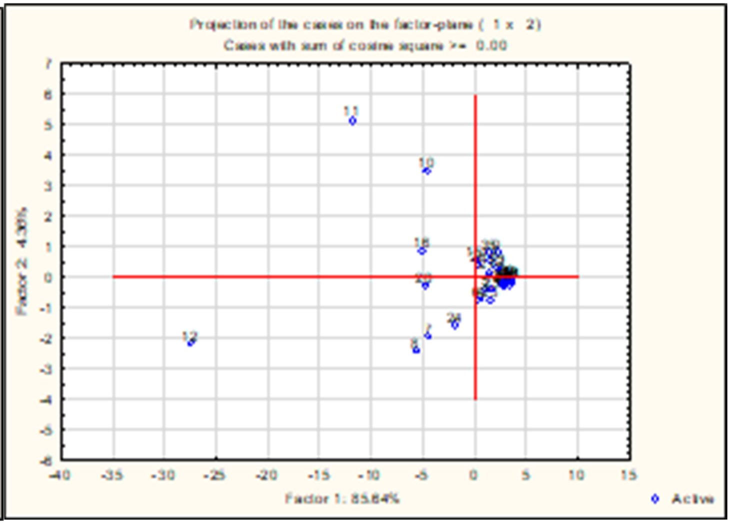

17F. DON and epidemic positions (n=36)

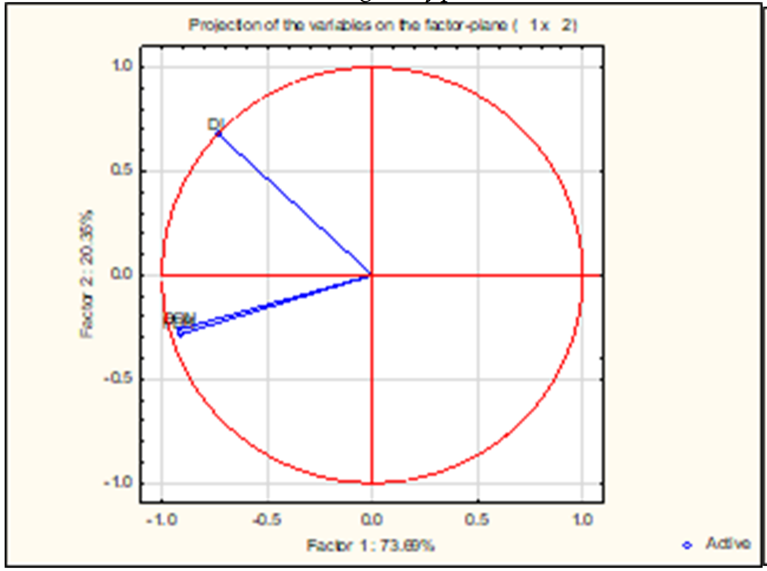

17G. Positions for the three pooled traits

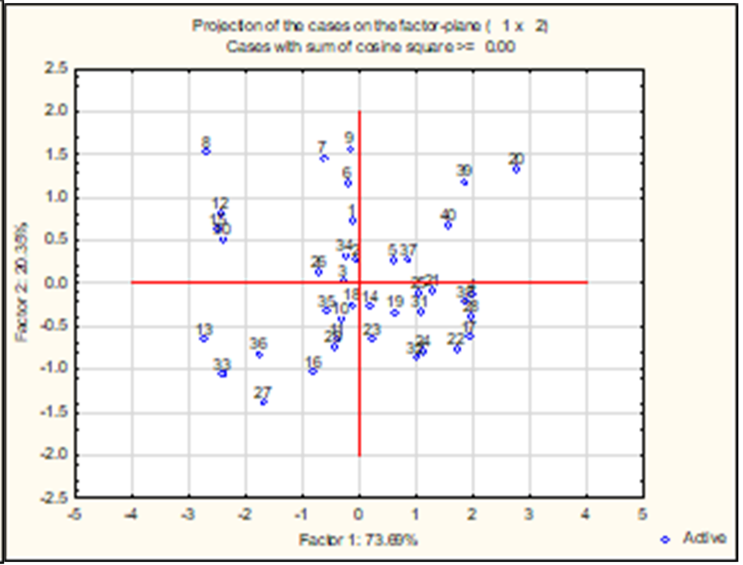

17H. Genotypes according to epidemics across traits

Figure 18. PCA analyses of the winter wheat FHB resistance tests for DI, FDK and DON contamination for genotypes and epidemics, 2019-2022.

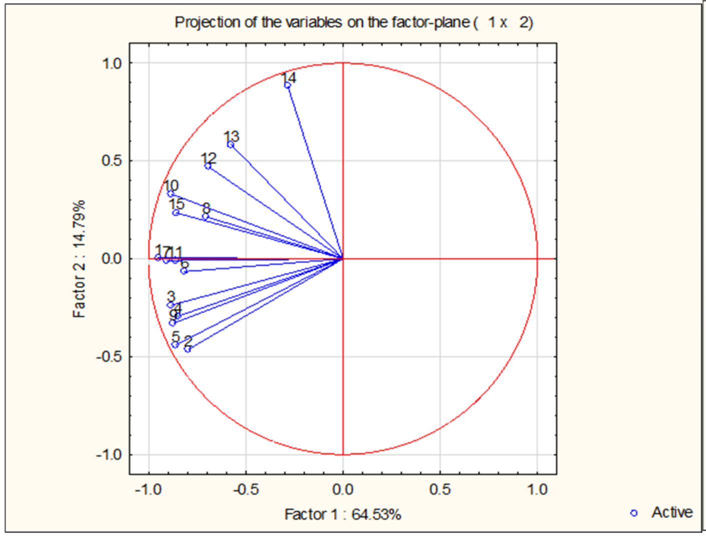

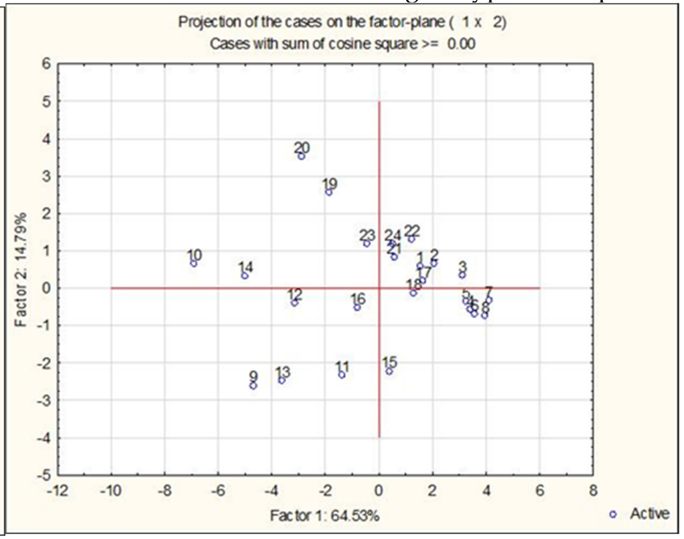

18A. Disease index in 15 genotype distributions

18B. Disease index in 24 epidemic situations

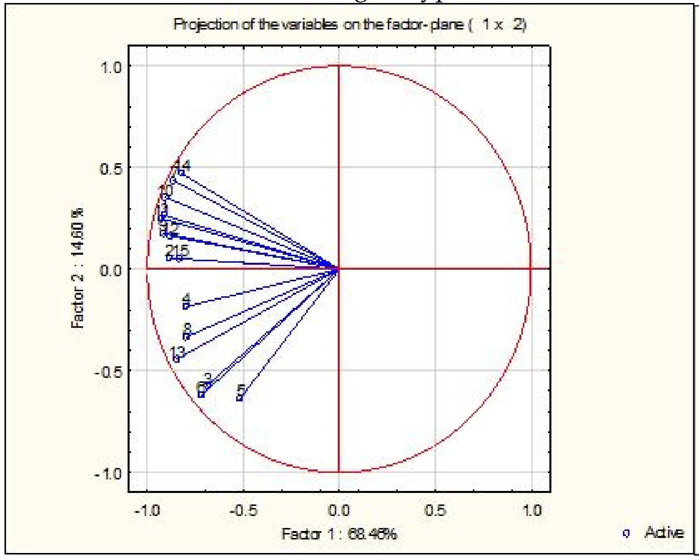

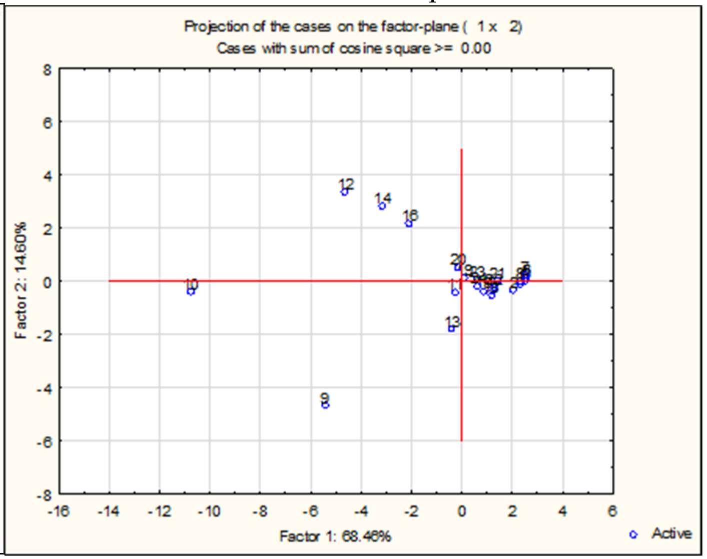

18C. FDK distribution in genotypes

18D. FDK distribution of epidemics

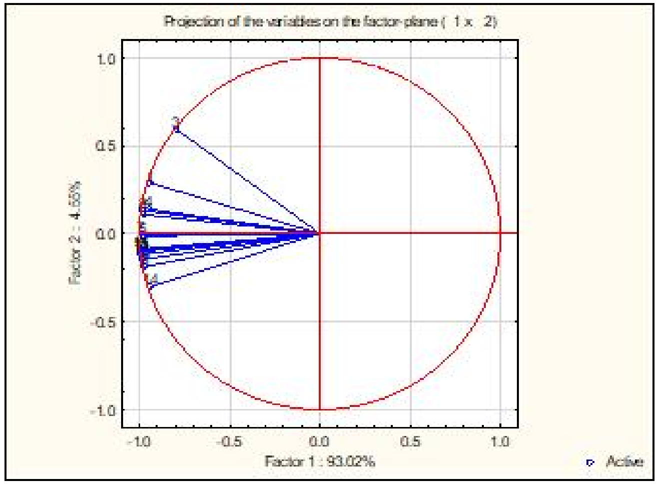

18E. DON distribution for genotypes (n=15)

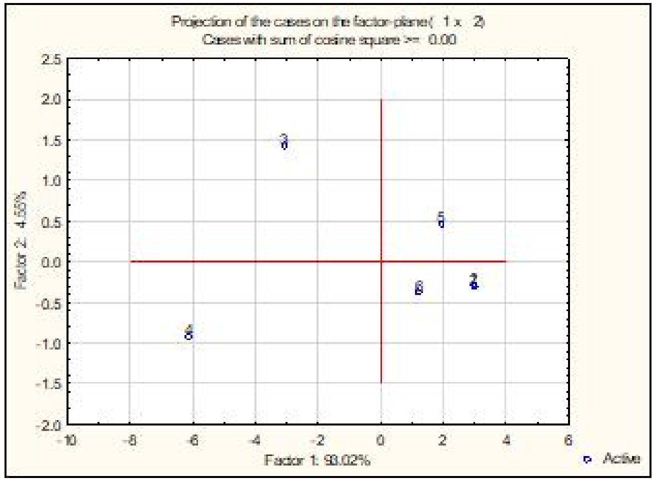

18F. DON distributions for epidemics

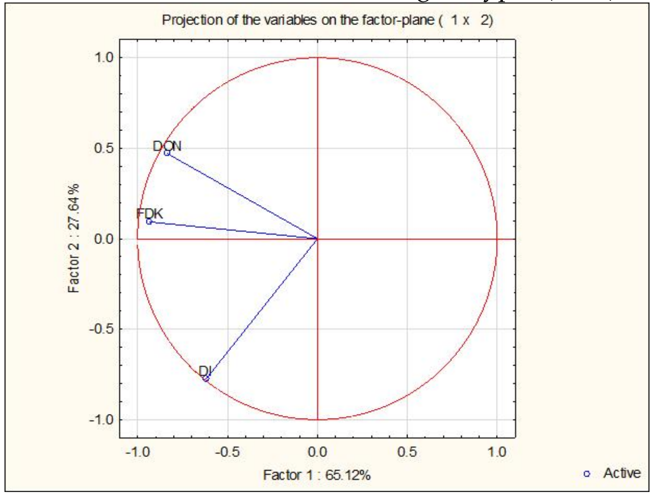

18G. Positions of the three traits

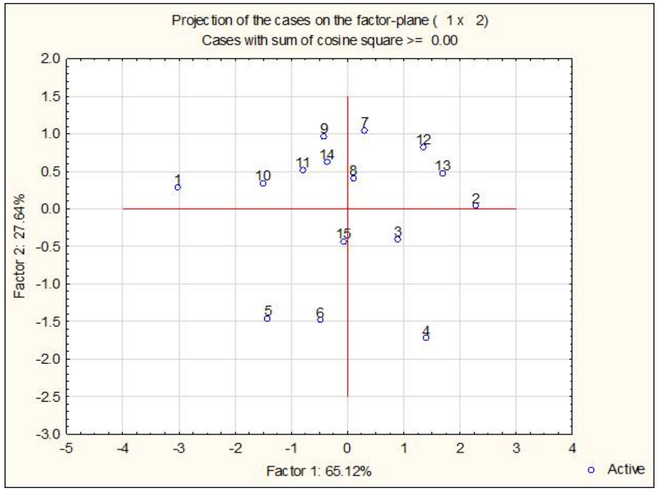

18H. Genotype positions across traits
